# Gut microbiota-mediated bile acid transformations regulate the transport of aflatoxin B1 from the intestine to the liver in piglets

**DOI:** 10.1186/s40104-025-01169-x

**Published:** 2025-03-08

**Authors:** Jiangdi Mao, Yusen Wei, Zhixiang Ni, Jinzhi Zhang, Junli Zhu, Haifeng Wang

**Affiliations:** 1 College of Animal Science, Zhejiang University, The Key Laboratory of Molecular Animal Nutrition, Ministry of Education, Hangzhou, 310000 China; 2 College of Food Science and Biotechnology, Food Safety Key Laboratory of Zhejiang Province, Zhejiang Gongshang University, Hangzhou, 310018 China

**Keywords:** Aflatoxin, Bile acid, CYP8B1, FXR, Microbiota, Piglet

## Abstract

**Background:**

Aflatoxins have been reported as a significant pollutant in feed, capable of causing harm to the liver, gastrointestinal tract and kidneys of piglets. However, research on the interactions among aflatoxin B1 (AFB1), bile acid (BA) metabolism and gut microbiota is limited.

**Methods:**

In this study, piglets were treated with AFB1 and antibiotics (ABX) to evaluate the interaction between AFB1 and gut microbiota. Subsequently, the roles of the farnesoid X receptor (FXR) and sterol 12α-hydroxylase (*CYP8B1*) in AFB1 absorption were studied by using FXR agonists obeticholic acid (OCA) and *Cyp8b1*-knockout (KO) mice, respectively.

**Result:**

AFB1 inhibited bile salt hydrolase (BSH) activity in ileal microbiota, downregulated ileal FXR expression, and upregulated *CYP8B1* expression in liver, increasing the proportion of 12α-OH BAs and potentially enhancing AFB1 absorption. ABX treatment reduced AFB1 absorption and liver damage, and unexpectedly increased BSH activity, counteracting the AFB1-induced downregulation of FXR and upregulation of *CYP8B1*. OCA reactivated ileal FXR, reduced AFB1 absorption, and alleviated liver damage. Furthermore, *Cyp8b1*-KO mice showed increased resistance to AFB1-induced liver damage by lowering AFB1 absorption.

**Conclusions:**

These results underscore the significance of gut microbiota and BAs in AFB1 absorption, suggesting new strategies to mitigate health risks from AFB1 in piglets.

**Supplementary Information:**

The online version contains supplementary material available at 10.1186/s40104-025-01169-x.

## Introduction

Mycotoxins are metabolic byproducts produced by fungi that grow on feed, posing significant health risks to both animals and humans. Aflatoxins are produced by *Aspergillus flavus* or *Aspergillus parasiticus*, with Aflatoxin B1 (AFB1) being the most harmful [1]. AFB1 has been demonstrated to be highly toxic, mutagenic, and carcinogenic in various animals [2, 3]. The liver is the primary target organ for AFB1 accumulations, leading to significant damage [4, 5]. Pigs, particularly piglets, are highly sensitive to aflatoxins. Feeding piglets a diet containing 280 ppb of AFB1 significantly reduces their feed intake and growth performance, while a diet with 140 ppb of AFB1 suppresses their immune function [6]. The ingestion of aflatoxins can lead to damage in the liver, gastrointestinal tract, and kidneys of pigs, as well as immune suppression, which subsequently impacts their health and growth performance [7]. Furthermore, aflatoxins can pose food safety concerns by accumulating in the organs and muscles of pigs [8].

Aflatoxins have been reported to interact with gut microbiota, exhibiting certain antimicrobial properties [9]. Additionally, aflatoxins can alter gut microbiota by exerting an impact on the host immune barrier or affecting goblet cells and the intestinal mucosal layer [10]. In turn, gut microbiota can mitigate the toxicity of aflatoxins through biotransformation [11–13] or adsorption [14, 15]. However, aflatoxins are primarily absorbed in the duodenum and jejunum of animals [16], where the microbial abundance is only one-thousandth or even one-millionth of that found in the colon [17]. Additionally, food residues or chyme have a longer residence time in the colon. Consequently, although the gut microbiota of monogastric animals such as pigs may possess the ability to degrade aflatoxins, it often lacks the optimal conditions and sufficient time for degradation before aflatoxins are absorbed by the small intestine [10, 18–20]. Ruminant animals generally exhibit greater tolerance to aflatoxins compared to monogastric animals, primarily due to the significant microbial population present in their rumen [10, 21, 22]. This anatomical characteristic facilitates an extended fermentation period for ingested food before it moves into the small intestine. Additionally, due to the low microbial abundance in the small intestine, we speculate that the gut microbiota of pigs has limited effectiveness in adsorbing mycotoxins within the small intestine. Therefore, without externally supplemented probiotics capable of adsorption, the intrinsic adsorption capacity of intestinal microbiota for AFB1 is minimal.

Bile acids (BAs) are natural surfactants derived from cholesterol, produced by mammalian hepatocytes and secreted into the duodenum. They play a crucial role in glucose metabolism, lipid digestion and regulation of gut microbiota. Cholesterol is converted into primary BAs by enzymes such as cholesterol 7α-hydroxylase (CYP7A1), sterol 12α-hydroxylase (CYP8B1), sterol 27-hydroxylase (CYP27A1), and oxysterol 7α-hydroxylase (CYP7B1) in the liver [23]. These primary BAs are then conjugated with glycine or taurine to form conjugated BAs, which are released into the duodenum to help digestion of food [24]. The bile salt hydrolase (BSH) activity of gut microbiota can convert conjugated BAs into unconjugated BAs and further transform unconjugated primary BAs into secondary BAs [24]. During this process, the gut microbiota alters the ratio of conjugated to unconjugated BAs, which affects the expression of ileal FXR [25, 26]. FGF15/19 transmits signals through the portal vein to the liver, regulating the expression of bile acid synthases such as *CYP7A1*, *CYP8B1* and *CYP7B1*, thereby altering the synthesis of bile acids in the host liver [27, 28].

While some research has suggested that aflatoxins can cause abnormalities in BA metabolism in animals, there is still limited research exploring the interaction between BAs and aflatoxins in depth. An earlier study reported that interrupting BA secretion into the intestine through bile duct cannulation in rats significantly reduced the absorption rate of mycotoxins [29]. Therefore, we hypothesize that gut microbiota of piglets may mediate the transformation and metabolism of BAs, playing a crucial role in the transport of the mycotoxin AFB1 from the intestine to the liver.

## Methods

### Animals

This research was specifically approved by the Animal Care and Use Committee (IACUC) of Zhejiang University (Ethics Code Permit ZJU20240795). All 48 piglets were raised under the same conditions on a farm located in Zhejiang Province, China. AFB1 was orally administered to piglets at a dosage of 30 μg/kg body weight (BW) once daily from d 4 to 24 (the day the piglets were born was considered as d 0), combined antibiotic (ampicillin, 1 g/L; vancomycin, 500 mg/L; neomycin sulfate, 1 g/L; and metronidazole, 1 g/L) was orally given to piglets at a dosage of 5 mL/kg BW once daily from d 1 to 24, and obeticholic acid (OCA) was orally administered to piglets at 6 mg/kg BW once daily from d 1 to 24.

For the AFB1 × antibiotics (ABX) study, 24 male piglets were randomly divided into 4 groups: Control group (CON), phosphate buffer saline (PBS) was orally administered to piglets from d 1 to 24; AFB1 group, PBS was orally administered to piglets from d 1 to 3, and AFB1 was orally administered to piglets from d 4 to 24; ABX group, combined antibiotic was orally given to piglets once daily from d 1 to 24; ABX + AFB1 group, combined antibiotic was orally given to piglets from d 1 to 24, and AFB1 was orally administered to piglets from d 4 to 24.

For the AFB1 × OCA study, 24 newborn male piglets were randomly divided into 4 groups: CON and AFB1, treatments same as above; OCA group, OCA was orally administered to piglets from d 1 to 24; OCA + AFB1 group, OCA was orally administered to piglets from d 1 to 24, and AFB1 was orally administered to piglets d 4 to 24.

Rectal swabs were collected on d 3, and blood samples were collected on d 24. Serum was obtained by centrifugation at 3,000 × *g* for 15 min at 4 °C, and stored at −80 °C until further analysis. On d 25, all piglets were euthanized via an intravenous injection of sodium pentobarbital solution (25 mg/kg BW). Liver segments, duodenal tissues, jejunal tissues and ileal tissues were fixed in 4% paraformaldehyde (PFA) for paraffin embedding. Liver samples, as well as tissues from ileum and colon, along with the contents of each intestinal segment, were frozen in liquid nitrogen separately and stored at −80 °C for extracting DNA, RNA, protein, AFB1 or lipids.

Specific pathogen-free (SPF) C57BL/6 J mice (male, 8-week-old) were utilized in this study. Both wild-type (WT) mice and *Cyp8b1*^*−*^KO mice were procured from GemPharmatech Co., Ltd. (Nanjing, China), and housed in a facility at Zhejiang University under controlled conditions of 22 ± 1 °C, 70% ± 5% humidity, and 12-h light/dark cycle. The primer sequences used for genotyping are available in Table S1. AFB1 was orally gavaged to mice at a dosage of 1 mg/kg BW once daily from 8 to 12-week-old. Twenty-four male mice were divided into 4 groups: CON (WT mice), AFB1 (WT mice treated with AFB1), *Cyp8b1*-KO (*Cyp8b1*-KO mice) and *Cyp8b1*-KO + AFB1 (*Cyp8b1*-KO mice treated with AFB1). After 4 weeks of AFB1 treatment, all mice were euthanized via an intravenous injection of sodium pentobarbital solution (50 mg/kg BW). Blood samples were collected, and serum was obtained by centrifugation at 3,000 × *g* for 15 min at 4 °C, then stored at −80 °C for later analysis. Liver segments were fixed in 4% PFA for paraffin embedding and stored in 2.5% glutaraldehyde for evaluation by transmission electron microscopy (TEM). Liver samples were individually frozen in liquid nitrogen and stored at −80 °C for RNA extraction.

Additionally, another 6 WT mice and 6 *Cyp8b1*-KO mice were euthanized 6 h after initial AFB1 gavage (20 μg AFB1 per mouse), and serum as well as contents from duodenum, jejunum, ileum, cecum and colon were collected. All feces and urine were collected together after initial AFB1 gavage. All samples were used to detect AFB1 concentration.

### Serum biochemistry

Serum levels of aspartate transaminase (AST), alanine transaminase (ALT), alkaline phosphatase (ALP), total bile acids (TBA), total cholesterol (TC), triglycerides (TG), high-density lipoprotein cholesterol (HDL-C) and low-density lipoprotein cholesterol (LDL-C) were measured using an automatic blood cell analyzer (XN-2000-A1, SYSMEX, Kobe, Japan).

### Enzyme-linked immunosorbent assay

The concentrations of lipopolysaccharide (LPS), diamine oxidase (DAO) and fibroblast growth factor 19 (FGF19) were measured using specific ELISA kits for LPS, DAO and porcine FGF19 (Ruixin Biological Technology Co., Ltd., Quanzhou, China).

AFB1 in the liver and intestinal contents was extracted using 70% methanol. The AFB1 concentrations were determined using an AFB1 ELISA kit (Pribolab, Qingdao, China). Add varying concentrations of AFB1 standards to liver and intestinal content samples that do not contain AFB1. Measure the AFB1 concentration to determine the recovery rate of AFB1 in the samples. The average recovery rates of AFB1 were shown in Table S2.

### Histopathological analysis and immunohistochemistry

Tissues, including liver, duodenum, jejunum and ileum, were fixed in 4% paraformaldehyde, dehydrated with ethanol, cleared with xylene, embedded in paraffin, sectioned into 4-μm-thick serial sections, and stained with hematoxylin and eosin (H&E) following deparaffinization.

For immunohistochemistry staining, samples underwent antigen retrieval using AR9 buffer (pH 6.0) at 96 °C, followed by permeabilization with normal goat serum for 20 min at 25 °C. After incubating with the primary (Aflatoxin Monoclonal Antibody, Thermo Fisher Scientific, Waltham, MA, USA) and secondary antibodies (Goat Anti-Mouse IgG, ZSGB-BIO, Beijing, China), the samples were visualized with diaminobenzidine (DAB). Finally, nuclei were counterstained with hematoxylin for 2 min.

### Transmission electron microscopy

Liver specimens were first fixed in 2.5% glutaraldehyde for 6 h, followed by postfixation in 1% OsO4 for 2 h. Afterward, they were rinsed three times with PBS buffer (0.1 mol/L, pH 7.0) for 15 min each. The specimens were then dehydrated through a graded ethanol series (30%, 50%, 70%, 80%) and acetone (90%, 95%, 100%). They were placed in a mixture of acetone and Spurr resin (1:1 v/v for 1 h, 1:3 v/v for 3 h), followed by pure Spurr resin overnight. The samples were then embedded and heated for 8 h at 65 °C. A Leica EM UC7 (Leica, Wetzlar, Germany) was used to section the samples, which were stained with uranyl acetate and alkaline lead citrate for 5–10 min each, and then observed using a H-7650 TEM (Hitachi, Tokyo, Japan).

### RNA extraction and RT-qPCR analyses

Livers were homogenized, and total RNA was extracted and purified using the SteadyPure Universal RNA Extraction Kit (Accurate Biotechnology Co., Ltd., Changsha, China). Reverse transcription (RT) was performed using the Evo M-MLV RT Kit (Accurate Biotechnology Co., Ltd., Changsha, China). Quantitative PCR (qPCR) was performed with the SYBR Green Premix Pro Taq HS qPCR Kit (Accurate Biotechnology Co., Ltd., Changsha, China) and the CFX96 Touch Real-Time PCR Detection System (Bio-Rad, Hercules, CA, USA). Relative quantities were calculated using GAPDH as the reference gene. Primer sequences are available in Table S3.

### Western blotting

Tissues were lysed with lysis buffer (Beyotime, Shanghai, China) following the manufacturer’s instructions. Protein concentrations in the samples were determined using BCA protein assay kit (Beyotime, Shanghai, China). Total protein samples were loaded onto SDS-PAGE gels for electrophoretic separation and subsequently transferred to polyvinylidene fluoride membranes, which were blocked and incubated with primary antibodies at 4 °C overnight. The primary antibodies included rabbit anti-FXR (Proteintech, Wuhan, China), anti-small heterodimer partner (SHP) (ABclonal, Wuhan, China), anti-ZO-1, anti-claudin-3, and anti-occludin (CST, Danvers, MA, USA). Then the membranes were washed four times for 10 min each in TBS-Tween-20, followed by incubation with secondary antibodies (goat anti-rabbit IgG, CST, Danvers, MA, USA). Specific proteins were detected using BeyoECL Star (Beyotime, Shanghai, China). Protein bands were visualized with a gel-documentation system (Thermo Fisher Scientific, Waltham, MA, USA) and analyzed with the Image J Software (National Institutes of Health, Bethesda, MD, USA). In all instances, the density values of bands were adjusted by subtracting background values. GAPDH was employed as the internal reference protein.

### BSH activity

Bacterial BSH activity was determined by quantifying the conversion of glycocholic acid (GCA) to cholic acid (CA). First, protein was extracted from 100 mg of ileal contents using 200 μL of PBS (pH 7.4) and sonication. The mixture was centrifuged, and 100 μL of the supernatant was collected. This was incubated with 10 μL of 0.1 mol/L GCA and 180 μL of PBS (pH 7.4) at 37 °C for 30 min. The reaction was stopped by adding 10 μL of trichloroacetic acid for 1 min, followed by centrifugation to collect the supernatant. Next, 1.9 mL of ninhydrin reagent (0.5 mL of 1% ninhydrin in 0.5 mol/L citrate buffer pH 5.5, 0.2 mL of 0.5 mol/L citrate buffer, and 1.2 mL of 30% glycerol) was added to the supernatant, vortexed, and boiled for 15 min. After cooling, absorbance was measured at 570 nm using taurine as the standard. One unit of BSH activity was defined as the enzyme amount that releases 1 mmol of amino acid per minute from the substrate.

### Statistical analysis

For the comparison of two groups, Student’s *t*-test was used. For the comparison of four groups, one-way ANOVA and Tukey’s post-test were used. *P* values < 0.05 were considered to be significant. All analyses were performed with the Graphpad Prism (v8.0).

Please see additional Methods in Additional file 1.

## Results

### Combination of antibiotics mitigated AFB1-induced liver damage in piglets by reducing AFB1 absorption

**Fig. 1:**
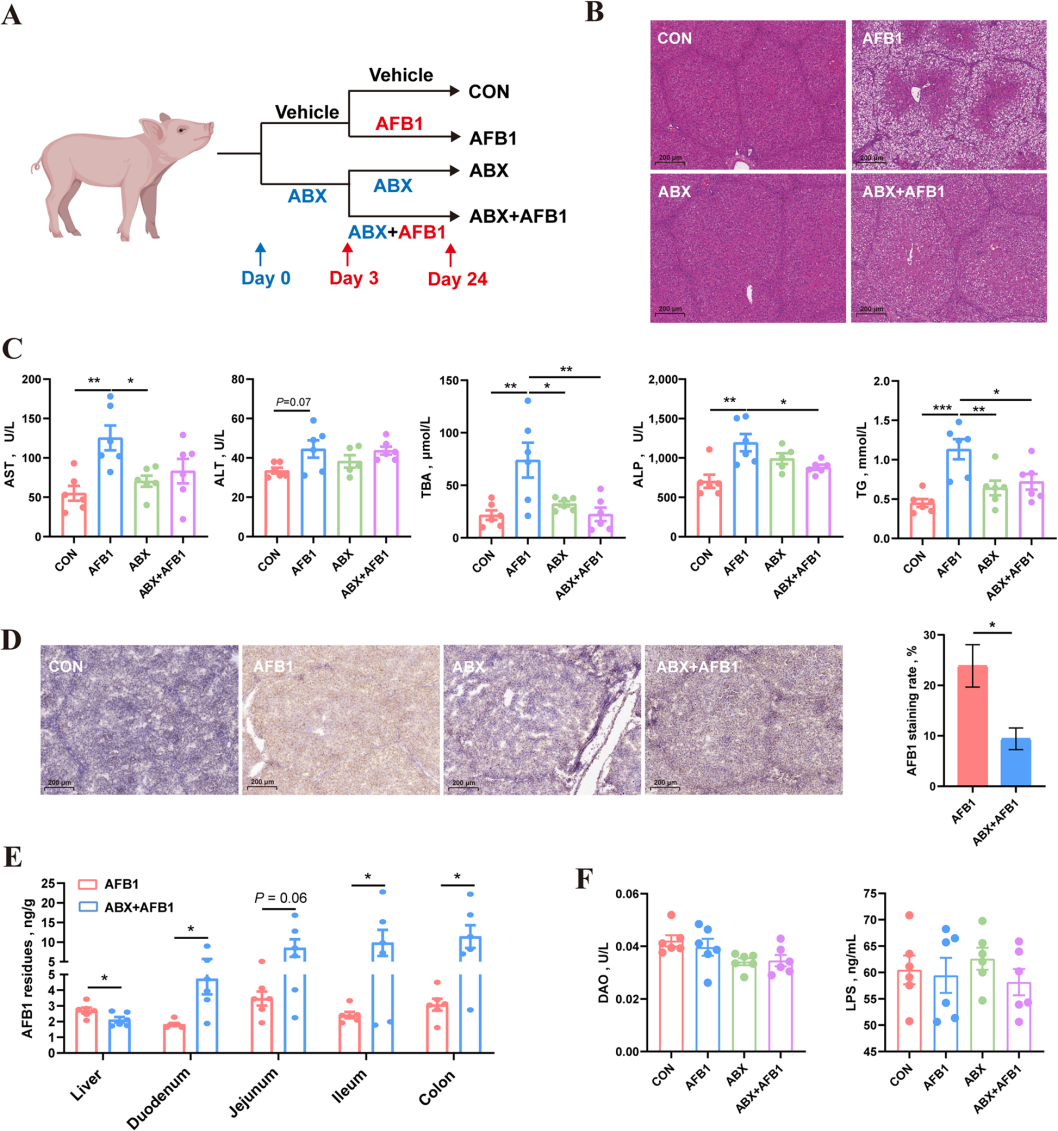
ABX mitigated AFB1-induced liver damage in piglets by reducing AFB1 absorption. **A** Experimental design. **B** Representative images of liver sections stained with H&E. **C** Levels of AST, ALT, TBA, ALP and TG in the serum of piglets (*n* = 6). **D** Representative images of liver sections with AFB1 immunohistochemical staining and the statistic of AFB1 staining rate (*n* = 3). **E** AFB1 residues in the liver and contents of each intestinal segment (*n* = 6). **F** Levels of DAO and LPS in the serum of piglets (*n* = 6). Statistical analysis: unpaired two-tailed Student’s *t-* test for the comparison of two groups, one-way ANOVA and Tukey’s post-test for the comparison of four groups (^***^P < 0.001; ^**^P < 0.01; ^*^P < 0.05)

Experimental design of the AFB1 × ABX study was shown in Fig. 1A. The 16S rRNA sequencing results of rectal swabs from piglets showed that after three days of ABX treatment, the α-diversity of rectal microbiota, including the ACE, Chao1, Shannon, and Simpson indexes, was significantly reduced (Fig. S1A). Additionally, the β-diversity of rectal microbiota in antibiotic-treated piglets exhibited a significant difference from the untreated piglets (Fig. S1B). The results from the Venn diagram (Fig. S1C), along with the relative abundance of microbiota at the phylum and genus levels (Fig. S1D), indicated that the composition of intestinal microbiota in piglets became considerably simpler, with *Escherichia-Shigella* and *Lactobacillus* becoming the dominant bacteria. These findings suggest that the gut microbiota in piglets was effectively cleared by ABX treatment before AFB1 treatment. Following three weeks of uninterrupted AFB1 ingestion, piglets displayed notable liver damage. Histopathological analysis of liver tissues unveiled pronounced hepatocyte vacuolization, a characteristic sign of hepatic steatosis (Fig. 1B). In piglets treated with AFB1, serum levels of biochemical markers such as AST, ALP, TBA, and TG as indicative of liver damage, significantly increased, while ABX treatment mitigated the rise in these serum markers caused by AFB1 (Fig. 1C). Additionally, there were no significant differences in serum TC levels among the four groups of piglets. Notably, the HDL-C and LDL-C levels in the serum of piglets treated with both ABX and AFB1 were significantly higher than those in untreated piglets (Fig. S2A). Immunohistochemical findings revealed a reduction in hepatic accumulation of AFB1 with ABX treatment (Fig. 1D). Additionally, ELISA analysis showed a decreased hepatic AFB1 accumulation post-ABX treatment, while AFB1 levels retained in the intestinal lumen (including the duodenum, jejunum, ileum, and colon) notably increased (Fig. 1E). This suggests that ABX treatment may alleviate AFB1-induced liver damage in piglets by reducing intestinal AFB1 absorption.

### AFB1 inhibits the ileal FXR pathway in piglets by altering the abundance of gut microbiota

**Fig. 2:**
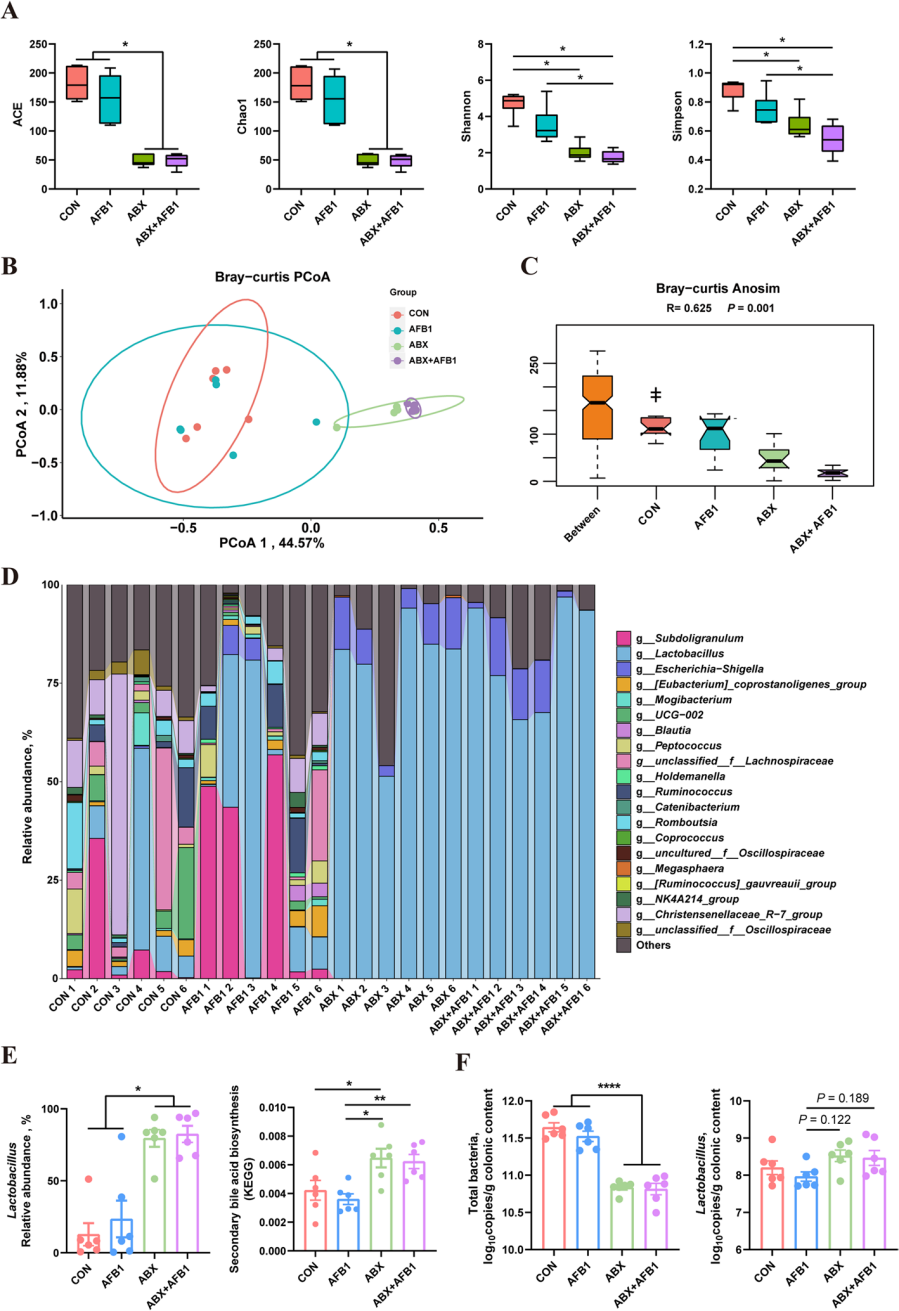
ABX treatment resulted in the enrichment of *Lactobacillus* in the colonic microbiota of the piglets. **A** ACE, Chao1, Shannon and Simpson indices in α-diversity analysis (*n* = 6). **B** Principal Coordinates Analysis (PCoA) of β diversity. **C** ANOSIM based on Bray-Curtis distance (*n* = 6). **D** Relative abundance of microbiota at genus level. **E** The relative abundance of *Lactobacillus* and levels of secondary bile acid biosynthesis (KEGG enrichment analysis) in colonic microbiota (*n* = 6). **F** The copy number of total bacteria and *Lactobacillus* in colonic contents (*n* = 6). Statistical analysis: one-way ANOVA and Tukey’s post-test for the comparison of four groups (^****^P < 0.0001; ^**^P < 0.01; ^*^P < 0.05)

**Fig. 3:**
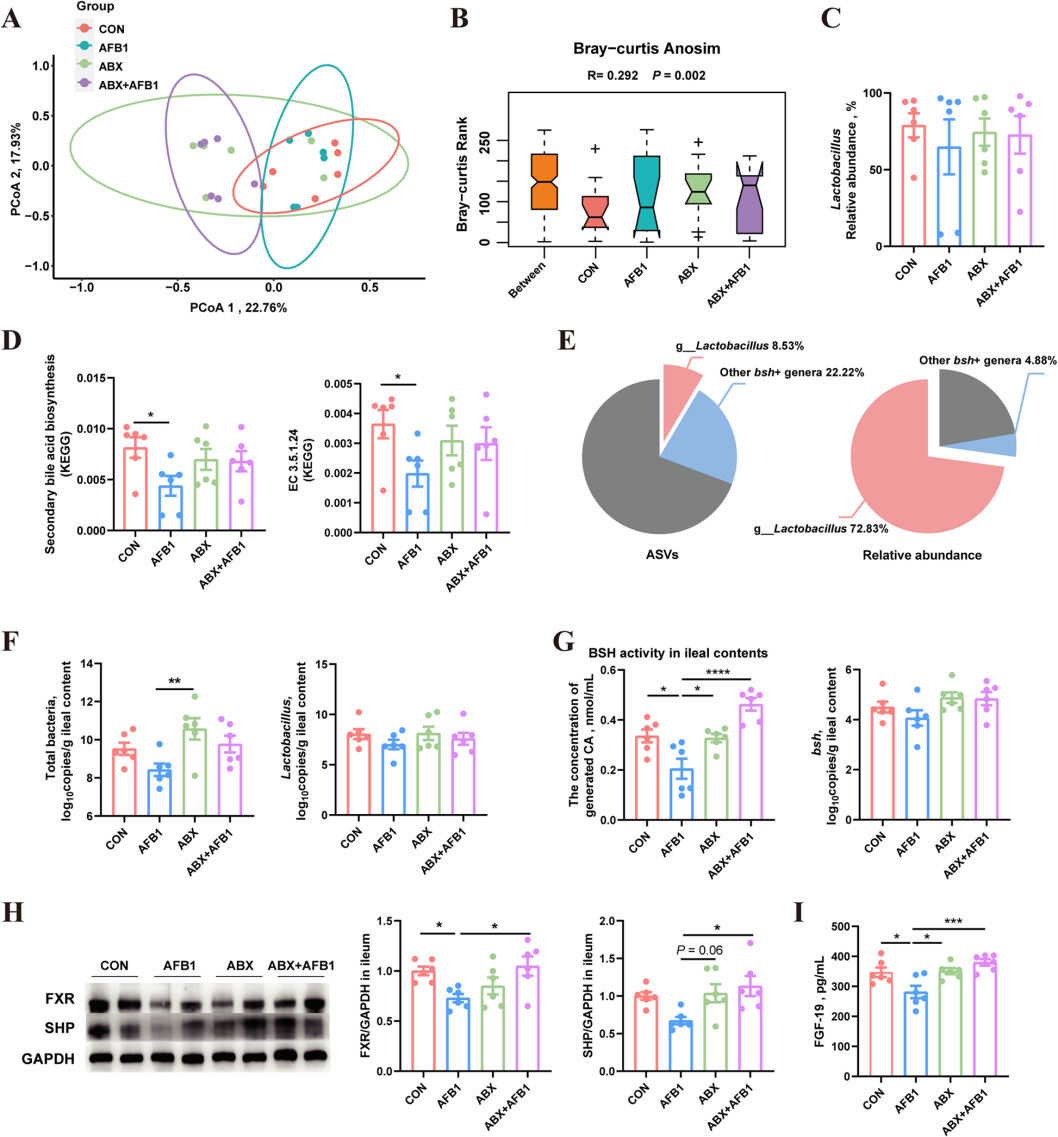
AFB1 inhibits the ileal FXR pathway in piglets by altering the ileal microbiota. **A** Principal Coordinates Analysis (PCoA) of β diversity. **B** ANOSIM based on Bray-Curtis distance (*n* = 6). **C** The relative abundance of *Lactobacillus* (*n* = 6). **D** Levels of secondary bile acid biosynthesis and EC 3.5.1.24 (KEGG, *n* = 6). **E** The ratio of g_*Lactobacillus* and other EC 3.5.1.24 positive genera among all ASVs, and their relative abundance in the ileal microbiota across all samples. **F** The copy number of total bacteria and *Lactobacillus* in ileal contents (*n* = 6). **G** BSH activity and the copy number of *bsh* gene in ileal contents (*n* = 6). **H** Protein expression of ileal FXR and SHP (*n* = 6). **I** Levels of FGF19 in the serum of piglets (*n* = 6). Statistical analysis: one-way ANOVA and Tukey’s post-test for the comparison of four groups (^****^P < 0.0001; ^***^P < 0.001; ^**^P < 0.01; ^*^P < 0.05)

The height of villi and the depth of crypts in the duodenum, jejunum, and ileum (Fig. S3A–C), as well as protein expression of colonic tight junction proteins (Fig. S3D) and serum levels of DAO and LPS (Fig. 1F) in piglets, showed no significant differences among the four groups. RNA sequencing results of the liver showed that ABX treatment did not cause significant changes in liver RNA levels of piglets (Fig. S4A and B), which excluded the possibility of direct liver impact from ABX treatment. This means that the main mode of action for antibiotics to alleviate liver damage caused by AFB1 treatment is still through changing the composition and abundance of gut microbiota. To assess the impact of AFB1 and ABX treatments on the piglets’ gut microbiota, we performed 16S rRNA sequencing of bacteria from ileal and colonic content samples. The index of ACE, Chao1, Shannon and Simpson showed that AFB1 treatment slightly reduced the α-diversity of colonic microbiota of piglets, while ABX treatment significantly reduced it (Fig. 2A). Clear separation was observed between the ABX treatment group (ABX and ABX + AFB1) and the non-ABX treatment group (CON and AFB1) (PCOA1 = 44.57%, *R* = 0.625, *P* = 0.001) when Bray-Curtis and analysis of similarity (ANOSIM) based on Bray-Curtis distance were used to evaluate the β-diversity of colonic microbiota (Fig. 2B, C). Additionally, the ANOSIM based on Bray-Curtis distance showed that the microbial composition significantly differed among all four groups, except between the CON and AFB1 groups (Table S4). Bacteria with top 13 and top 20 relative abundances at the phylum (Fig. S5) and genus levels (Fig. 2D) were shown, respectively. After ABX treatment, the relative abundance of *Lactobacillus* in the piglet colon significantly increased, becoming the dominant bacterial group within the colonic microbiota (Fig. 2E). KEGG pathway enrichment performed using the PICRUSt2 indicated that ABX treatment significantly improved secondary BA synthesis. The simplified secondary BA biosynthesis pathway diagram was shown in Fig. S5B. EC 3.5.1.24 (choloylglycine hydrolase) plays a role in this pathway by hydrolyzing glycine or taurine from conjugated BAs. The levels of EC 3.5.1.24 performed using the PICRUSt2 showed that ABX treatment significantly improved the relative abundance of microbes capable of producing BSH (Fig. S5C). Quantitative PCR was used to measure the absolute abundance of total bacteria and *Lactobacillus* in colonic contents. The results showed that ABX treatment led to a significant reduction in the total microbial abundance in the piglet colon (Fig. 2F). Furthermore, absolute qPCR results indicated that ABX treatment not decreased but actually increased the absolute abundance of *Lactobacillus* in the colon. Unlike the significant differences observed in the colonic microbiota, the variations in ileal microbiota among the four groups of piglets were considerably smaller. Index of ACE, Chao1, Shannon and Simpson showed that neither ABX nor AFB1 treatment significantly affected the α-diversity of ileal microbiota in piglets (Fig. S6A). Bray-Curtis principal coordinates analysis (PCoA) (Fig. 3A) and ANOSIM based on Bray-Curtis distance (Fig. 3B) was used for the β-diversity analysis and revealed that there were significant differences between CON and ABX + AFB1, AFB1 and ABX, and AFB1 and ABX + AFB1 (Table S5). The relative abundance of ileal microbiota at the phylum and genus levels is shown (Fig. S6B, C). *Lactobacillus* was the dominant genus in the ileal microbiota of all four groups of piglets (Fig. S6B), and no significant differences were observed in the relative abundance of *Lactobacillus* among the four groups (Fig. 3C). The Significantly altered KEGG pathways in the CON and AFB1 groups are shown in Fig. S6D (PICRUSt2, *P* < 0.05, FC > 1.5). AFB1 treatment significantly reduced secondary BA synthesis and the expression of EC 3.5.1.24, while ABX treatment partially mitigated this reduction (Fig. 3D). The potential of all ASVs in the ileal microbiota of piglets to express EC 3.5.1.24 is shown in Table S6. The results indicated that all ASVs annotated as g_*Lactobacillus* can express EC 3.5.1.24, representing 8.53% of the total ASVs, but accounting for 72.83% of the total relative abundance in the ileal microbiota of all piglets (Fig. 3E). In contrast, the relative abundance of other microbes capable of expressing EC 3.5.1.24 is only 4.88% (Fig. 3E). Considering that 16S rRNA sequencing reflects the relative abundance of microbiota, absolute quantification qPCR also be used to measure the total bacterial absolute abundance and the absolute abundance of *Lactobacillus* in ileal contents. The absolute abundance results of bacterial DNA indicated that AFB1 treatment appeared to decrease the absolute abundance of ileal microbiota in piglets, while ABX treatment actually increased the absolute abundance of ileal microbiota (Fig. 3F). The absolute abundance of *Lactobacillus* did not show significant differences among the four groups.

In this study, AFB1 treatment significantly reduced the BSH activity of ileal microbiota in piglets, while ABX treatment not only did not decrease microbial BSH activity but also mitigated the reduction in BSH activity caused by AFB1 treatment (Fig. 3G). Consequently, the expression of proteins in the piglet ileum assessed via Western-blotting revealed that AFB1 treatment lowered the expression of FXR (Significantly, *P* < 0.05) and SHP proteins compared with the control, whereas, ABX treatment significantly reversed this reduction (*P* < 0.05) (Fig. 3H). Furthermore, serum FGF19 levels were measured using ELISA, showing that AFB1 treatment decreased serum FGF19 levels in piglets, while ABX treatment mitigated this decrease (Fig. 3I).

### AFB1 enhances the expression of hepatic *CYP8B1* and modifies BA synthesis in piglets

**Fig. 4:**
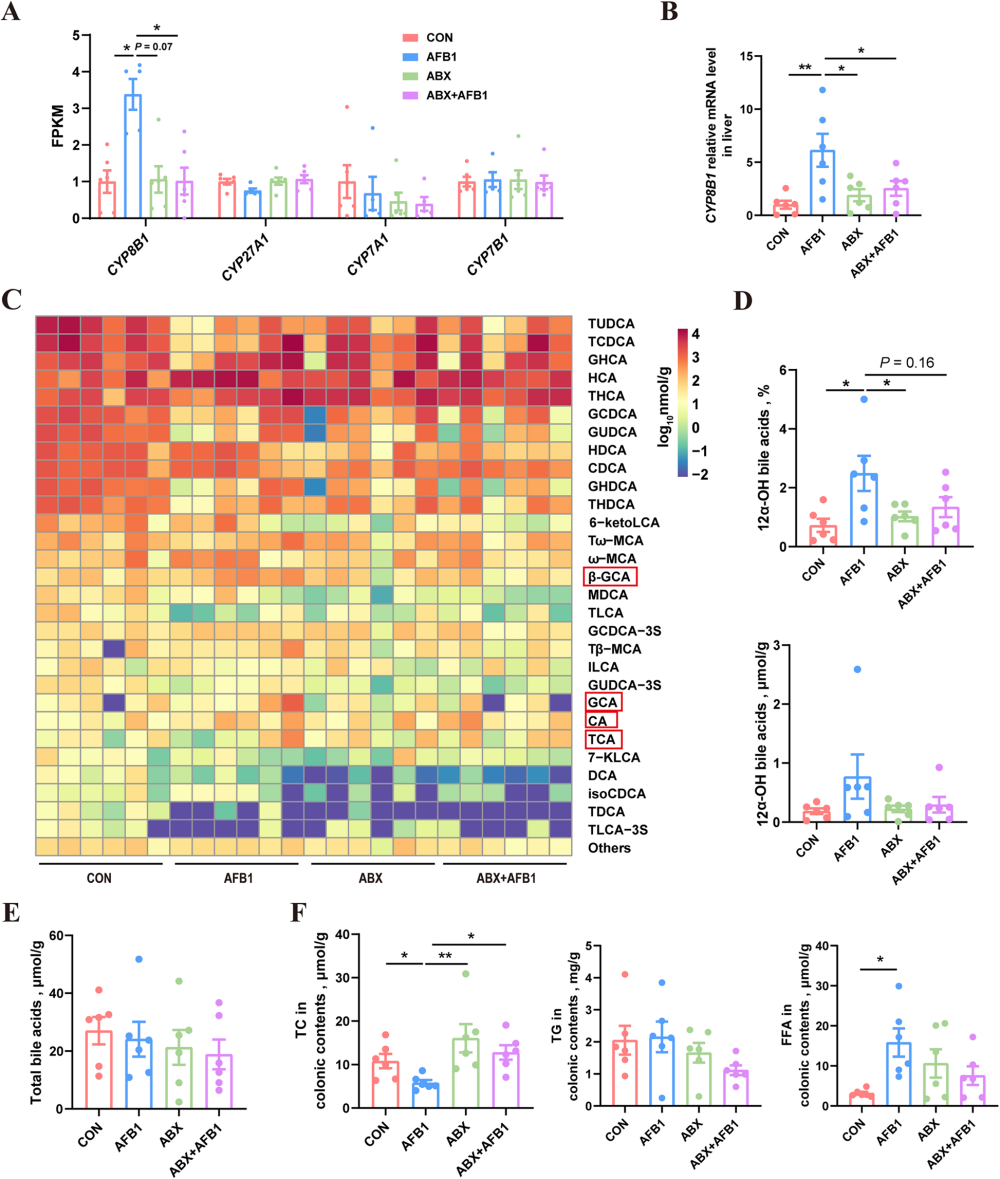
AFB1 enhances the expression of hepatic *CYP8B1* and modifies bile acid synthesis in piglets. **A** Expression levels of hepatic *CYP8B1*, *CYP27A1*, *CYP7A1* and *CYP7B1* as determined by RNA-seq (*n* = 5 or 6). **B** Relative mRNA expression of hepatic *CYP8B1* measured by qPCR (*n* = 6). **C** Heatmap showing the concentration of different BAs in jejunal contents. **D** Proportion and concentration of 12α-OH BAs in jejunal contents (*n* = 6). **E** The concentration of total bile acids in jejunal contents (*n* = 6). **F** Concentration of TC, TG and FFA in the colonic contents (*n* = 6). Statistical analysis: one-way ANOVA and Tukey’s post-test for the comparison of four groups (^**^P < 0.01; ^*^P < 0.05)

To evaluate whether the downregulation of the ileal FXR pathway caused by AFB1 leads to alterations in hepatic BA synthesis and metabolism in piglets, we used transcriptome analysis and qPCR to examine the expression of genes involved in BA synthesis, including *CYP7A1*, *CYP27A1*, *CYP7B1* and *CYP8B1*. The liver transcriptome analysis revealed significant changes occurred in only one gene related to BA synthesis, specifically *CYP8B1*, following AFB1 treatment. However, the upregulation of *CYP8B1* induced by AFB1 was inhibited by ABX treatment (Fig. 4A). No significant differences were observed in the expression of other genes associated with BA synthesis among the four experimental groups. The qPCR results for these gene expressions were consistent with those obtained from the transcriptome analysis (Fig. 4B, Fig. S2B).

BAs primarily aid in the absorption of lipids in the duodenum and jejunum. Therefore, we analyzed the BA composition in the jejunal contents of piglets to assess the practical impact of changes in hepatic BA synthesis (Fig. 4C). We observed a significant increase in the proportion of 12-αOH BAs, which include CA and its conjugated BAs, GCA and TCA, as well as their derivative BAs, such as β-GCA (red box in Fig. 4C), in response to AFB1 treatment (Fig. 4D). Notably, this increase was mitigated by ABX treatment (Fig. 4D). The total BA concentrations in the jejunum of the four groups of piglets showed no significant differences (Fig. 4E). Furthermore, changes in BA composition could potentially affect the piglets’ ability to absorb other lipid substances. Therefore, we assessed the levels of lipids, including TC, TG, and FFA, in the colons of piglets. TC residue levels were found to be decreased in the colons of piglets treated with AFB1, whereas ABX treatment inhibited this reduction (Fig. 4F). The TG residue levels in the colon showed no significant differences among the four groups of piglets. However, the FFA residue levels were significantly higher in the colon of AFB1-treated piglets compared to untreated piglets (Fig. 4F).

### FXR agonists mitigated AFB1-induced liver damage in piglets by reducing AFB1 absorption

**Fig. 5:**
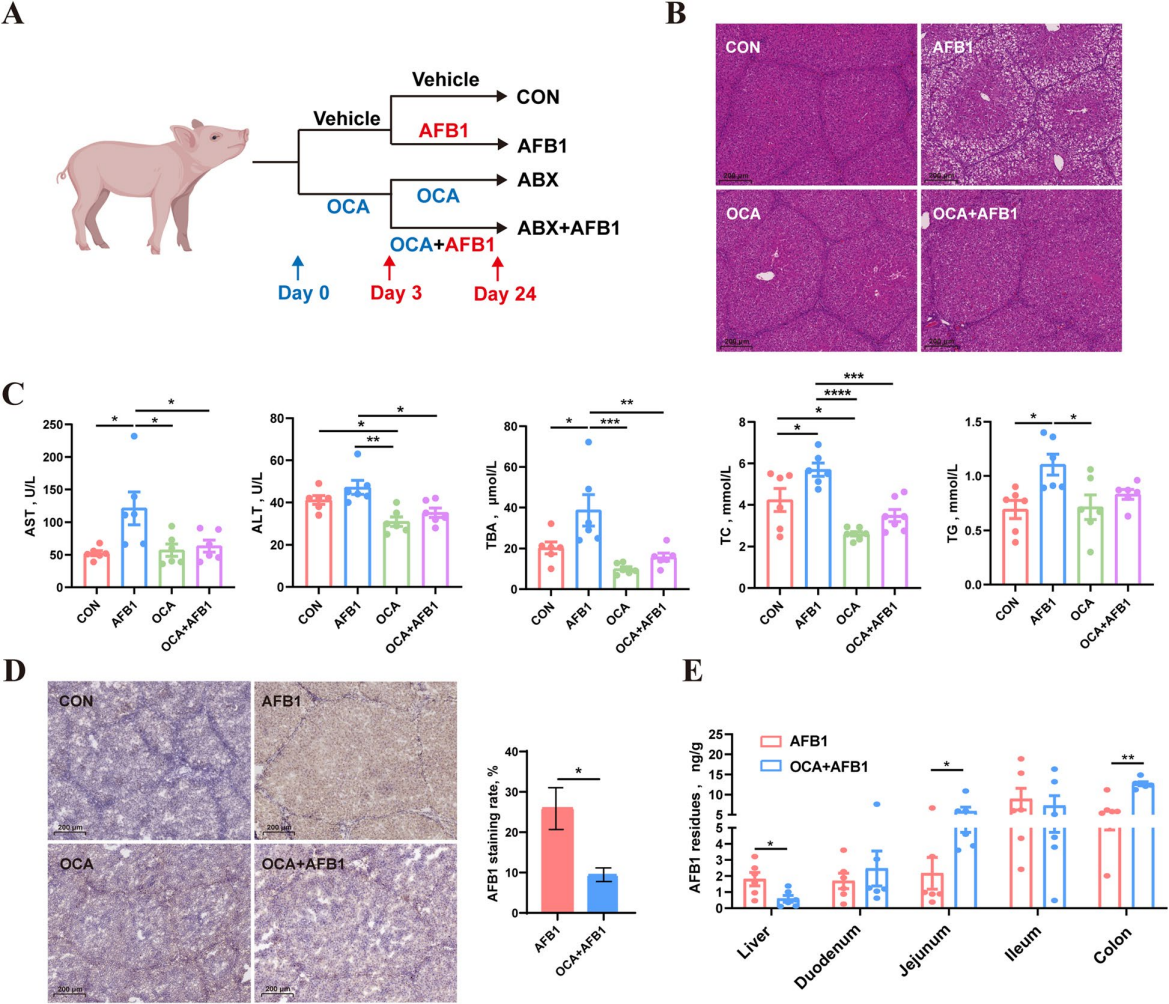
OCA mitigated AFB1-induced liver damage in piglets by reducing AFB1 absorption. **A** Experimental design. **B** Representative images of liver sections stained with H&E. **C** Levels of AST, ALT, TBA, TC and TG in the serum of piglets (*n* = 6). **D** Representative images of liver sections with AFB1 immunohistochemistry staining and the statistic of AFB1 staining rate (*n* = 3). **E** AFB1 residues in the liver and contents of each intestinal segment (*n* = 6). Statistical analysis: unpaired two-tailed Student’s *t*-test for the comparison of two groups, one-way ANOVA and Tukey’s post-test for the comparison of four groups (^****^P < 0.0001; ^***^P < 0.001; ^**^P < 0.01; ^*^P < 0.05)

To illustrate the critical role of the ileal FXR pathway in AFB1 absorption, we administered the FXR agonist OCA to piglets (Fig. 5A). Histological analysis of liver tissue using H&E staining indicated that OCA treatment effectively alleviated the hepatocellular vacuolation caused by AFB1 treatment (Fig. 5B). Serum levels of AST, ALT, TBA, TC, and TG increased significantly in the AFB1 group compared to the CON group. In contrast, these levels decreased with the administration of OCA in the OCA + AFB1 group, indicating that OCA treatment effectively alleviated AFB1-induced liver damage (Fig. 5C). Additionally, OCA treatment significantly reduced the levels of HDL-C and LDL-C in the serum of piglets (Fig. S7A). Immunohistochemical analysis showed that OCA treatment reduced the accumulation of AFB1 in the piglets’ livers (Fig. 5D), however, led to increased residual AFB1 quantified by ELISA in the jejunum and colon of piglets (Fig. 5E). These findings suggest that the FXR agonist OCA effectively mitigated AFB1-induced liver injury in piglets, possibly by reducing AFB1 absorption.

### FXR agonists effectively mitigate the suppression of the ileal FXR pathway and the upregulation of hepatic *CYP8B1* induced by AFB1

**Fig. 6:**
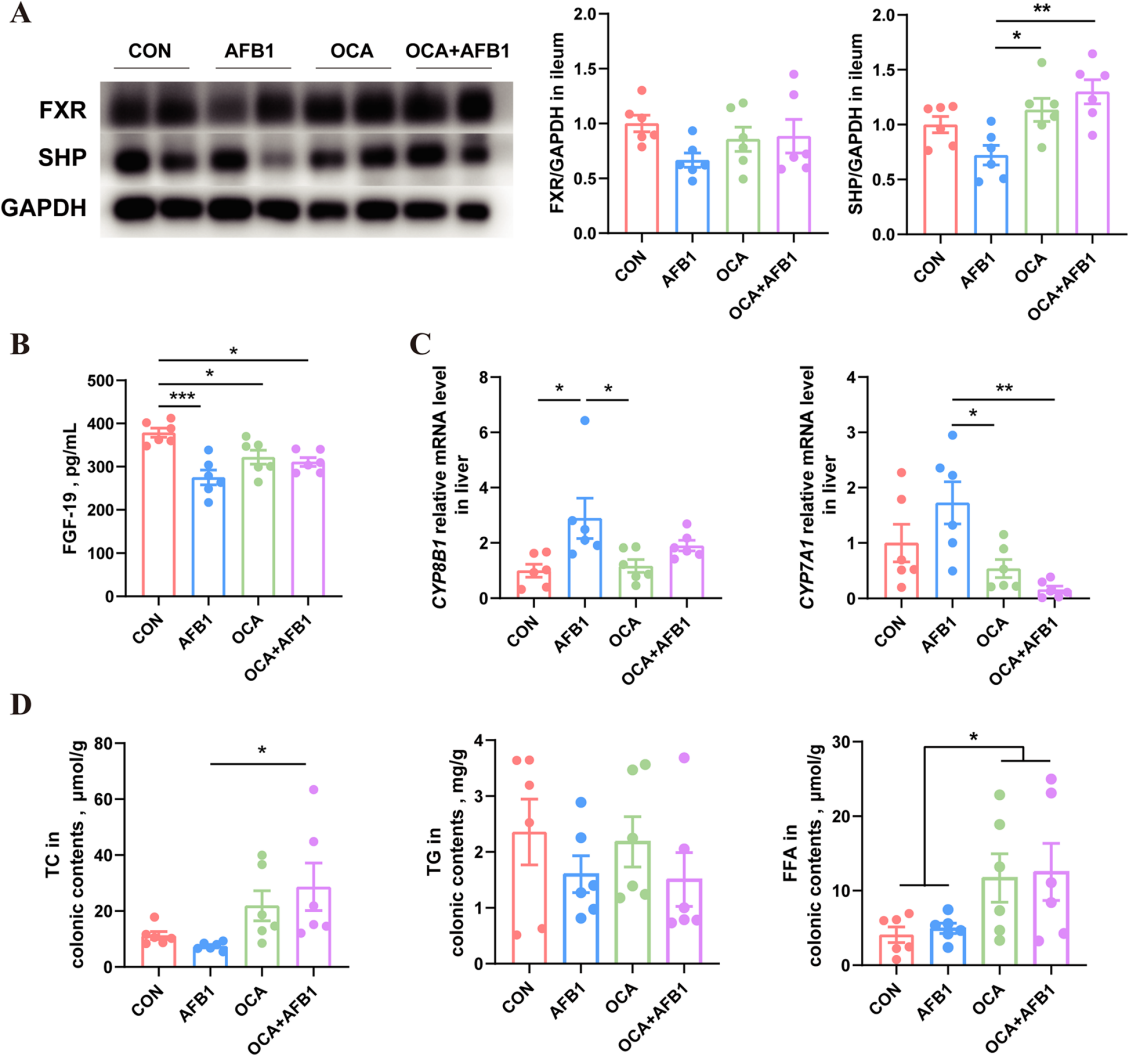
OCA effectively alleviates the inhibition of the ileal FXR pathway and the upregulation of hepatic *CYP8B1* caused by AFB1. **A** Protein expression levels of ileal FXR and SHP (*n* = 6). **B** Levels of FGF19 in the serum of piglets (*n* = 6). **C** Relative mRNA expression of hepatic *CYP8B1* and *CYP7A1* (*n* = 6). **D** Concentration of TC, TG and FFA in the colonic contents (*n* = 6). Statistical analysis: one-way ANOVA and Tukey’s post-test for the comparison of four groups (^***^P < 0.001; ^**^P < 0.01; ^*^P < 0.05)

Western blot analysis of ileal proteins showed that the average expression levels of FXR and SHP in the ileum of piglets treated with AFB1 decreased compared to the other three groups. Specifically, the AFB1 group exhibited a marked reduction in SHP expression in the ileum when compared to both groups treated with OCA (*P* < 0.05) (Fig. 6A). Additionally, the ELISA results showed that serum FGF-19 levels in AFB1-treated piglets were significantly higher than those in the CON group, but not significantly higher than those in the OCA + AFB1 group (Fig. 6B).

An analysis of *CYP8B1* and *CYP7A1* mRNA levels in piglet livers indicated that AFB1 treatment significantly increased *CYP8B1* expression, while OCA treatment partially mitigated the upregulation of *CYP8B1* induced by AFB1. Furthermore, in contrast to ABX, OCA treatment significantly reduced *CYP7A1* expression in piglet livers (*P* < 0.05) (Fig. 6C). Additionally, we assessed the levels of lipid substances, including TC, TG, and FFA, in the colons of piglets. The TC content was significantly higher in the OCA + AFB1 group compare to the AFB1 group (*P* < 0.05) (Fig. 6D). Moreover, neither AFB1 nor OCA treatment had a significant effect on absorption of TG in piglets. However, unlike ABX treatment, OCA treatment significantly increased the levels of FFA in the colons of piglets (Fig. 6D).

### *Cyp8b1*-KO mice exhibit reduced absorption of AFB1 compared to WT mice

**Fig. 7:**
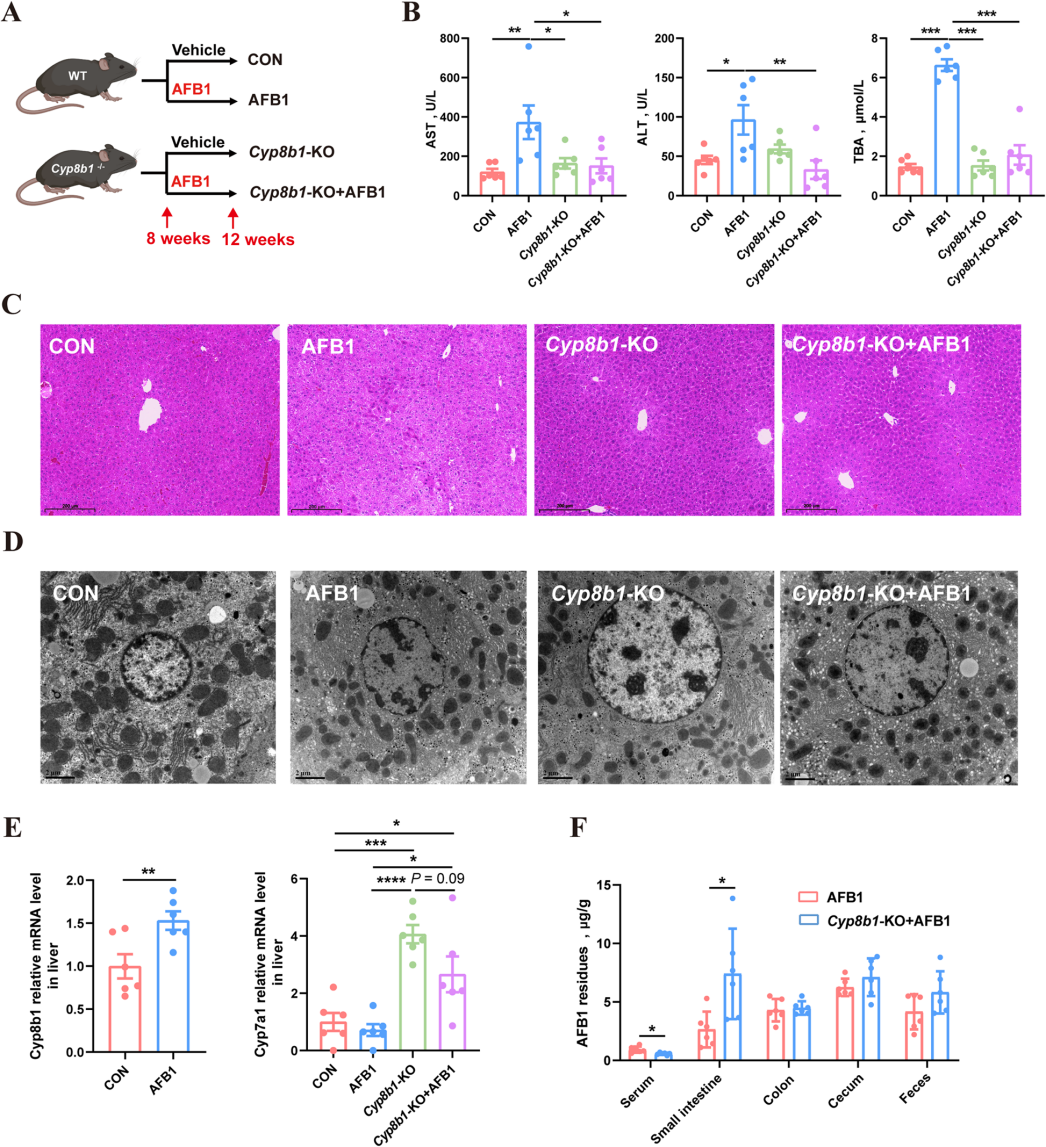
*Cyp8b1*-KO mice exhibit reduced absorption of AFB1 compared to WT mice. **A** Experimental design. **B** Levels of AST, ALT, TBA in the serum of mice (*n* = 6). **C** Representative images of liver sections stained with H&E. **D** TEM of liver sections. **E** Relative mRNA expression of hepatic *Cyp8b1* and *Cyp7a1* (*n* = 6). **F** AFB1 residues in the serum, contents of each intestinal segment and feces of mice (*n* = 6). Statistical analysis: unpaired two-tailed Student’s *t*-test for the comparison of two groups, one-way ANOVA and Tukey’s post-test for the comparison of four groups (^****^P < 0.0001; ^***^P < 0.001; ^**^P < 0.01; ^*^P < 0.05)

To verify the importance of the *CYP8B1* gene in the absorption of AFB1 in animals, we administered AFB1 to *Cyp8b1* knockout (KO) mice (Fig. 7A). The mice were genotyped prior to treatment (Fig. S8). AFB1 treatment resulted in a significant increase in serum levels of AST, ALT, and TBA, which are indicators of liver damage, in WT mice (Fig. 7B). In contrast, *Cyp8b1*-KO mice did not show significant elevations in these markers following AFB1 treatment, indicating that *Cyp8b1*-KO mice can effectively mitigate AFB1-induced liver damage. This finding underscores the critical role of the *CYP8B1* gene in the mechanism of AFB1-induced liver injury.

Histopathological examination of liver tissue using H&E staining further supported these findings. AFB1-treated WT mice exhibited marked hepatic vacuolation, whereas *Cyp8b1*-KO mice showed significantly less liver damage following AFB1 treatment (Fig. 7C). TEM of liver tissues revealed nuclear and mitochondrion abnormalities in the hepatocytes of AFB1-treated WT mice, while the cellular organelles in *Cyp8b1*-KO mice remained relatively normal, even when treated with AFB1 (Fig. 7D). The results of qPCR analysis of liver BA synthesis-related genes showed that AFB1 treatment also significantly increased the expression of *Cyp8b1* in the livers of mice, however, no *Cyp8b1* mRNA expression was detected in the livers of *Cyp8b1*-KO mice (Fig. 7E). Interestingly, the knockout of the *Cyp8b1* gene resulted in a significant increase in the expression of the *Cyp7a1* gene. Neither AFB1 treatment nor *Cyp8b1* knockout significantly affected the expression of *Cyp27a1* and *Cyp7b1* genes (Fig. S9B).

To further investigate the significance of the *Cyp8b1* gene in AFB1 absorption, another six WT mice and six *Cyp8b1*-KO mice were euthanized 6 h after AFB1 gavage, and serum and intestinal content samples were collected to measure AFB1 concentration. The results indicated that, following the initial AFB1 gavage, the serum AFB1 concentration in WT mice was significantly higher than in *Cyp8b1*-KO mice, while the AFB1 concentration in the small intestine of WT mice was notably lower than in *Cyp8b1*-KO mice (Fig. 7F).

Due to variations in the weight of intestinal contents and feces among individual mice, no significant differences were observed in the total amounts of AFB1 calculated from the weights and concentrations of AFB1 in the intestinal contents and feces between the two groups (Fig. S9C). However, the total unabsorbed AFB1 tended to be lower in WT mice compared to *Cyp8b1*-KO mice (*P* = 0.10) (Fig. S9C).

## Discussion

Existing studies have extensively investigated the biochemical processes and mechanisms by which AFB1 is metabolized and produces toxicity within the body [4, 5]. In the liver, AFB1 is enzymatically converted into metabolites such as AFB1-8,9 epoxide (AFBO) and aflatoxin M1 (AFM1) by enzymes including cytochrome P450 enzyme 1A2 (CYP1A2) and cytochrome P450 enzyme 3A4 (CYP3A4), leading to inflammation, injury, and carcinogenesis [4, 5]. Notably, AFBO has two stereoisomers: exo-AFBO and endo-AFBO [30]. Only exo-AFBO binds to DNA at the N7 position of guanine [31, 32], while endo-AFBO is rapidly excreted through conjugation with glutathione-*S*-transferase [32]. However, the absorption process of aflatoxins remains inadequately explored. Although early studies reported that the vast majority of AFB1 is absorbed in the duodenum and jejunum [33], no study has yet fully investigated the mechanisms of mycotoxin absorption or the roles of gut microbiota and BAs in this process. Here, we unexpectedly discovered that ABX treatment alleviated AFB1-induced liver damage in piglets by reducing AFB1 absorption, potentially mediated through the gut microbiota-BA axis.

In this study, ABX treatment effectively alleviated AFB1-induced liver damage in piglets. Additionally, ABX treatment increased the residual AFB1 in the intestinal lumen of piglets, resulting in reduced accumulation of AFB1 in the liver. This indicates that ABX treatment effectively decreased the intestinal absorption of AFB1 in piglets. While previous studies have shown that AFB1 treatment causes intestinal barrier damage in animals [34, 35], both histopathological analyses of the small intestine and the expression levels of tight junction proteins in the colon indicated that AFB1 treatment did not cause significant damage to the intestinal barrier of the piglets in this study. Notably, the absence of significant differences in the serum levels of DAO and LPS, which reflect the integrity of the intestinal barrier, strongly supports this conclusion. Therefore, it is deemed that the integrity of the intestinal barrier is not the key factor contributing to the variation in AFB1 absorption in piglets.

The results of 16S rRNA sequencing of ileal contents indicated that ABX treatment did not significantly alter the microbial composition of the ileum in piglets. However, KEGG functional prediction from 16S rRNA sequencing of ileal microbiota indicated that AFB1 treatment significantly reduced the secondary BA synthesis function and level of EC 3.5.1.24 in ileal microbiota, which was partially mitigated by ABX treatment. EC 3.5.1.24 has been reported to be almost synonymous with BSH [24, 36]. In this study, all ASVs annotated as g_*Lactobacillus* have the potential to express BSH, with their relative abundance constituting approximately 93.7% of all microbes capable of expressing BSH in the ileum. This suggests that the BSH level in the ileum of piglets is predominantly influenced by the abundance and activity of *Lactobacillus*. *Lactobacillus* is widely reported to have the ability to produce BSH, but the capacity for BSH production varies significantly among different *Lactobacillus* strains [37–39]. In this study, although AFB1 treatment did not significantly reduce the absolute or relative abundance of *Lactobacillus* in the ileum of piglets, it did significantly decrease in both the predicted BSH activity based on KEGG functional analysis and the actual measured BSH activity. This reduction may be attributed to AFB1 treatment altering the species composition within the *Lactobacillus* genus, resulting in a decline in the abundance of high-BSH-producing strains. Additionally, the relative abundance of *Lactobacillus* significantly increased in the rectal swabs and colonic contents of piglets following ABX treatment. This suggests that ABX treatment may have created a more favorable environment for the growth and metabolism of *Lactobacillus*. *Lactobacillus,* a beneficial symbiotic gut bacterium, was identified as a reservoir of antibiotic resistance genes [40]. A previous study also reported that AFB1 treatment decreased BSH activity in the feces of mice [41]. Recent studies have reported that BSH activity of ileal microbiota can regulate the expression of ileal FXR by modulating the ratio of conjugated to unconjugated BAs, thereby influencing hepatic BA synthesis [27, 28]. The results showed that AFB1 treatment indeed decreased the expression level of FXR protein in the ileum of piglets, leading to reduced downstream SHP protein expression and serum FGF19 levels. ABX treatment effectively mitigated the AFB1-induced inhibition of the ileal FXR pathway. In this study, we unexpectedly found that ABX treatment increased BSH activity within the ileal microbiota of suckling piglets. We believe this result does not imply that ABX treatment directly mitigated AFB1-induced damage; rather, it suggests that the increase in BSH activity within the ileal microbiota may have indirectly lessened the harmful effects of AFB1. Additionally, the effectiveness of ABX treatment observed in this study may be attributed to the piglets being in the nursing phase, a developmental stage in which the gut microbiota is predominantly composed of *Lactobacillus*. In future studies, we plan to further investigate this protective effect by supplementing germ-free animals (rodents and pigs) with BSH-producing microorganisms or externally administering BSH that remains active in vivo.

Some studies have reported that alterations in the expression of the FXR pathway in the ileum can lead to changes in BA synthesis and metabolism in the liver. As previously mentioned, the synthesis of BAs in the liver requires several enzymes, including CYP7A1, CYP8B1, CYP27A1, and CYP7B1. Among these, CYP7A1 is the rate-limiting enzyme that determines the total amount of BA synthesis; CYP8B1 is essential for the synthesis of 12α-OH BAs, determining the proportion of 12α-OH BAs in total BAs; and CYP27A1 and CYP7B1 determine the proportion of non-12α-OH BAs in total BAs [23, 42]. Although numerous studies have reported the relationship between the ileal FXR pathway and the expression of these enzymes, the specific genes affected vary across different studies. Studies by Goodwin et al*.* [43] and Lu et al*.* [44] indicate that changes in FXR expression in the ileum affect *CYP7A1* expression, while studies by Kuang et al*.* [45] and Worthmann et al*.* [46] suggest that changes in ileal FXR expression influence *CYP7B1* expression. Additionally, previous studies [47, 48] have shown that changes in ileal FXR expression impact hepatic *CYP8B1* expression. However, there is insufficient research on the mechanisms through which ileal FXR influences the expression of specific BA synthesis enzymes in the liver. In this study, AFB1 treatment resulted in the overexpression of *CYP8B1* without affecting the expression of other BA synthesis enzymes. ABX treatment reduced the AFB1-induced overexpression of *CYP8B1*. Targeted BA metabolomics of jejunal contents indicated that AFB1 treatment increased the proportion of 12α-OH BAs, which are synthesized by the enzyme CYP8B1. A previous study reported that the ratio of 12α-OH to non-12α-OH BAs was markedly decreased in antibiotic-treated hamsters [49]. Therefore, it is supposed that the overexpression of *CYP8B1* and the corresponding increase in 12α-OH BAs may play a crucial role in AFB1 absorption. Since BA synthesis and metabolism typically affect lipid absorption in the small intestine, we measured the concentrations of three lipid substances–TC, TG, and FFA–in the colonic contents of piglets. Higher concentrations of these substances in the colon suggest lower absorption in the small intestine. The results show that only the trend in TC absorption corresponds with that of AFB1. Specifically, AFB1 treatment increased cholesterol absorption in the piglets, whereas ABX treatment decreased it. Previous research has shown that inhibiting hepatic *CYP8B1* expression in mice can reduce cholesterol absorption [50].

To investigate the impact of ileal FXR expression on AFB1 absorption, we treated piglets with OCA, an orally active FXR agonist. OCA has been validated in various animals for its ability to successfully activate ileal FXR expression. It has been reported that OCA, by activating the ileal FXR pathway, can inhibit hepatic BA synthesis and secretion, thereby reducing lipid absorption and alleviating liver fat accumulation. This makes it a promising candidate for mitigating the progression of metabolic dysfunction-associated steatotic liver disease (MASLD) [51, 52]. Additionally, OCA has been shown to inhibit hepatic *CYP8B1* expression in animals [48, 53]. Our results indicate that OCA treatment effectively activated the ileal FXR pathway in piglets and reduced AFB1 absorption, thereby alleviating AFB1-induced liver damage. Furthermore, AFB1 treatment increased cholesterol absorption in piglets, while OCA treatment decreased cholesterol absorption. This suggests a potential link between AFB1 and cholesterol absorption, indicating that the pathways involved in cholesterol absorption may also play significant roles in AFB1 absorption. This will be a key focus of our future research.

To investigate the significance of CYP8B1 for AFB1 absorption, we treated *Cyp8b1*-KO mice with AFB1. The reason we administered a dose of 1 mg/kg BW of AFB1 to mice is that they exhibit high activity of glutathione *S*-transferases and a strong tolerance to AFB1 [54]. In our previous studies, doses of 100 µg/kg BW and 300 µg/kg BW did not result in significant liver damage in the mice. These results indicated that *Cyp8b1*-KO mice exhibited significantly less liver damage from AFB1 exposure compared to WT mice, suggesting that the *Cyp8b1* gene plays a crucial role in of AFB1-induced liver damage. Although mice and pigs are different species, we observed a similar upregulation of liver *Cyp8b1* expression due to AFB1 treatment in mice as seen in piglets. Additionally, we found that knocking out *Cyp8b1* significantly upregulated *Cyp7a1* expression in mouse livers, consistent with previous reports [42]. This may be a compensatory mechanism by the body to counteract the functional loss of BAs due to the deficiency of 12α-OH BAs by synthesizing more total of BAs. Furthermore, we observed that AFB1 treatment tended to reduce *Cyp7a1* expression in mouse livers, particularly in *Cyp8b1*-KO mice. Research on AFB1 and BA metabolism remains limited; however, Liu et al*.* [41] reported that AFB1 treatment significantly upregulated *Cyp7a1* expression in WT mouse livers. Therefore, we are currently uncertain about how to interpret this phenomenon. Samples collected 6 h after the initial AFB1 treatment showed that serum AFB1 concentrations in *Cyp8b1*-KO mice were significantly lower than those in WT mice. Additionally, the concentration of residual AFB1 in the small intestines of *Cyp8b1*-KO mice was significantly higher than in WT mice, further supporting the role of *Cyp8b1* as a key gene facilitating AFB1 absorption.

## Conclusions

**Fig. 8:**
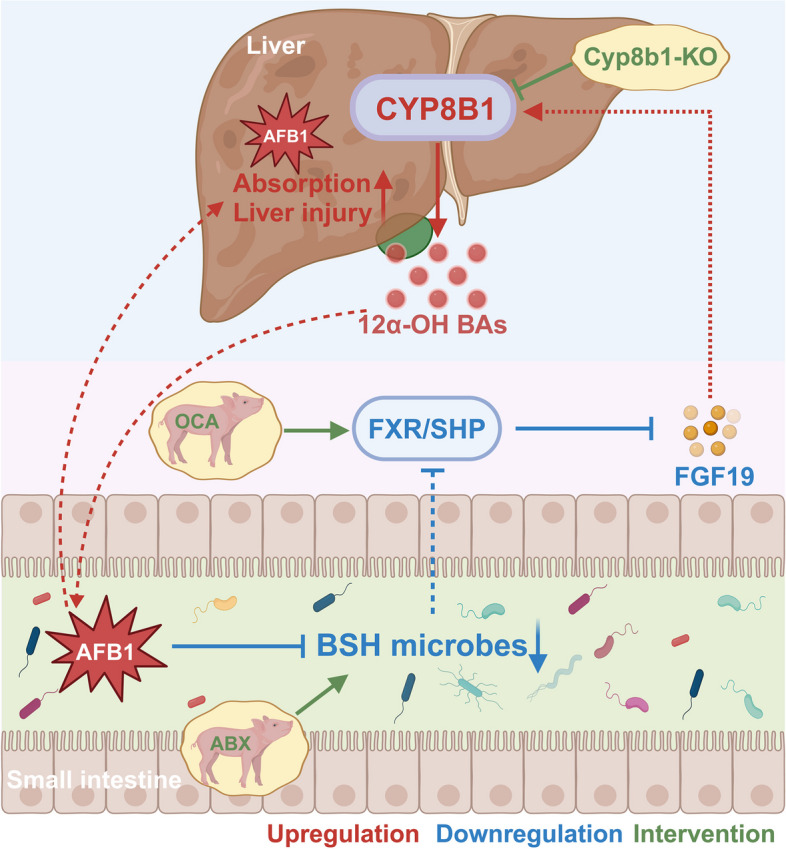
The proposed mechanism by which aflatoxin affects its toxicity via the gut microbiota-bile acid axis. AFB1 inhibits the BSH activity of ileal microbiota and upregulates hepatic *CYP8B1* expression by suppressing the ileal FXR pathway, then the increased expression of *CYP8B1* results in a higher proportion of 12α-OH BAs in bile acids, which facilitates the absorption of AFB1 in animals

In summary, our results indicate that AFB1 inhibits the BSH activity of ileal microbiota and upregulates hepatic *CYP8B1* expression by suppressing the ileal FXR pathway. The increased expression of *CYP8B1* results in a higher proportion of 12α-OH BAs in BAs, which facilitates the absorption of AFB1 in the intestine. Furthermore, the enhancing BSH activity in the ileal microbiota, the activation of the ileal FXR pathway with FXR agonists, or the inhibition of liver *CYP8B1* expression can all reduce the intestinal absorption of AFB1 in piglets to some extent, thereby alleviating AFB1-induced liver injury (Fig. 8). The overall results of this study suggest new strategies to mitigate health risks from AFB1 in piglets.

## Supplementary Information

Additional file 1: Method S1. 16S rRNA amplicon sequencing and data analyses. Method S2. Bile acid analysis. Method S3. RNA-seq. Table S1. Primer sequences used for *Cyp8b1*-KO genotyping. Table S2. Average recovery rates of AFB1 in samples. Table S3. Primer sequences for qPCR analysis. Table S4. Dissimilarities of colonic microbiota between piglets in different groups revealed by ANOSIM based on Bray-Curtis distance. Table S5. Dissimilarities of ileal microbiota between piglets in different groups revealed by ANOSIM based on Bray-Curtis distance. Fig. S1. ABX cleared the gut microbiota of piglets on d 3. Fig. S2. Effect of AFB1 and ABX treatments on piglets. Fig. S3. Effect of AFB1 and ABX on the intestinal barrier of piglets. Fig. S4. Overview of RNA-seq. Fig. S5. Effect of AFB1 and ABX treatment on colonic microbiota of piglets. Fig. S6. Effect of AFB1 and ABX treatment on ileal microbiota of piglets. Fig. S7. Effect of AFB1 and OCA treatments on piglets. Fig. S8. Results of *Cyp8b1*-KO mice genotyping. Fig. S9. Effect of AFB1 treatment on WT mice and *Cyp8b1*-KO mice.

Additional file 2: Table S6. EC 3.5.1.24 (choloylglycine hydrolase) activity prediction of ASVs revealed by PICRUSt2 with KEGG pathway database.

## Data Availability

All the relevant data supporting these findings are available in this report. In particular, 16S rRNA sequence data are available in the Sequence Read Archive (SRA) under BioProject accession PRJNA1163955; RNA-seq data are available in the SRA under BioProject accession PRJNA1163533.

## Abbreviations

ABX: Antibiotics
AFB1: Aflatoxin B1
AFBO: AFB1-8,9 epoxide
AFM1: Aflatoxin M1
ALP: Alkaline phosphatase
ALT: Alanine transaminase
AST: Aspartate transaminase
BA: Bile acid
BSH: Bile salt hydrolase
BW: Body weight
CA: Cholic acid
CYP1A2: Cytochrome P450 enzyme 1A2
CYP27A1: Sterol 27-hydroxylase
CYP3A4: Cytochrome P450 enzyme 3A4
CYP7A1: Cholesterol 7α-hydroxylase
CYP7B1: Oxysterol 7α-hydroxylase
CYP8B1: Sterol 12α-hydroxylase
DAB: Diaminobenzidine
DAO: Diamine oxidase
FGF: Fibroblast growth factor
FXR: Farnesoid X receptor
GCA: Glycocholic acid
HDL-C: High-density lipoprotein cholesterol
KO: Knockout
LDL-C: Low-density lipoprotein cholesterol
LPS: Lipopolysaccharide
OCA: Obeticholic acid
PBS: Phosphate buffer saline
PCoA: Principal coordinates analysis
PFA: Paraformaldehyde
qPCR: Quantitative PCR
RT: Reverse transcription
SHP: Small heterodimer partner
SPF: Specific pathogen-free
TBA: Total bile acids
TC: Total cholesterol
TG: Triglycerides
WT: Wild-type
