# Supplementary material for: Gut microbiota-mediated bile acid transformations regulate the transport of aflatoxin B1 from the intestine to the liver in piglets
Source: J Anim Sci Biotechnol. 2025 Mar 8;16:38. doi: 10.1186/s40104-025-01169-x (PMC11889867; doi:10.1186/s40104-025-01169-x)
Supplement: Supplementary file 1 — Additional file 1: Method S1. 16S rRNA amplicon sequencing and data analyses. Method S2. Bile acid analysis. Method S3. RNA-seq. Table S1. Primer sequences used for Cyp8b1-KO genotyping. Table S2. Average recovery rates of AFB1 in samples. Table S3. Primer sequences for qPCR analysis. Table S4. Dissimilarities of colonic microbiota between piglets in different groups revealed by ANOSIM based on Bray-Curtis distance. Table S5. Dissimilarities of ileal microbiota between piglets in different groups revealed by ANOSIM based on Bray-Curtis distance. Fig. S1. ABX cleared the gut microbiota of piglets on d 3. Fig. S2. Effect of AFB1 and ABX treatments on piglets. Fig. S3. Effect of AFB1 and ABX on the intestinal barrier of piglets. Fig. S4. Overview of RNA-seq. Fig. S5. Effect of AFB1 and ABX treatment on colonic microbiota of piglets. Fig. S6. Effect of AFB1 and ABX treatment on ileal microbiota of piglets. Fig. S7. Effect of AFB1 and OCA treatments on piglets. Fig. S8. Results of Cyp8b1-KO mice genotyping. Fig. S9. Effect of AFB1 treatment on WT mice and Cyp8b1-KO mice. [file 40104_2025_1169_MOESM1_ESM.docx]

**Supplementary material**

**Method**

**16S rRNA amplicon sequencing and data analyses**

The rectal swabs, ileal contents and colonic contents were used for DNA extraction and 16s rRNA gene sequencing. Microbial DNA was extracted using the QIAamp Fast DNA Stool Minikit (Qiagen, Hilden, Germany) according to manufacturer’s protocol. The V3-V4 region of the bacteria 16S rRNA genes were amplified, extracted, purified, quantified and sequenced as previously described. Briefly, the V3-V4 region of the bacteria 16S rRNA genes were amplified by PCR (95°C for 3 min, followed by 30 cycles at 98°C for 20 s, 58°C for 15 s, 72°C for 20 s and a final extension at 72 °C for 5 min) with primers 341F 5'-CCTACGGGRSGCAGCAG)-3' and 806R 5'-GGACTACVVGGGTATCTAATC-3’. PCR reactions were performed in 30 μL mixture containing 15 μL of 2× KAPA Library Amplification Ready Mix, 1 μL of each primer (10 μmol/L), 50 ng of template DNA and ddH2O. Amplicons were extracted from 2% agarose gels and purified using the AxyPrep DNA Gel Extraction Kit (Axygen Biosciences, Union City, CA, U.S.) according to the manufacturer’s instructions and quantified using Qubit®2.0 (Invitrogen, U.S.). After preparation of library, these tags were sequenced on MiSeq/HiSeq platform (Illumina, Inc., CA, USA) for paired end reads of 500/250 bp.

Illumina MiSeq fastq reads were imported into the QIIME2 platform (version 2021.2) and were processed by the Deblur program, which obtains single-nucleotide resolution based on error profiles within samples. In this study, these sequences were assigned to bacterial features, which are synonymous to ASVs, ESVs, and sub-OTUs and sequences between different features differed at the single-nucleotide level. Deblur generates unique features that could be compared between different studies. The taxonomy of these features was assigned to the silva reference database (138 version) classifier with 99% similarity. A feature table was generated using Qiime2’s qiime vsearch cluster-features-closed-reference command. Determination of alpha and beta diversities were also conducted in qiime2. Predictive metabolic functional analysis was performed using the PICRUSt2 plugin for QIIME2.

**Bile acid analysis**

Add 50 μL of internal standard working solution (200 ng/mL) and 350 μL of extraction solution (methanol) into 100 ug jejunal contents, vortex and mix for 30 s, sonicate at low temperature for 30 min (5°C, 40 KHz), than store at -20°C for 30 min, centrifuge at 4°C for 15 min at 13000 rcf, remove the supernatant and blow dry with nitrogen, add 100 μL of 50% acetonitrile, vortex and mix for 30 s, sonicate at low temperature for 10 min (5°C, 40 KHz), centrifuge at 4°C for 15 min at 13000 rcf, remove the supernatant for LC-MS/MS. The supernatant was purged with nitrogen, added 100 μL of 50% acetonitrile, vortexed and mixed for 30 s, sonicated at low temperature for 10 min (5℃, 40 KHz), centrifuged at 4℃ for 15 min at 13000 rcf, and the supernatant was taken for LC-MS/MS analysis.

The LC-MS/MS analysis of sample was conducted on a ExionLC AD system coupled with a QTRAP® 6500+ mass spectrometer (Sciex, USA) at Majorbio Bio-Pharm Technology Co. Ltd. (Shanghai, China). Briefly, samples were separated by an Waters BEH C18 (150*2.1 mm,1.7 μm) and thermostated at 50℃. The mobile phases consisted of water solution (with 0.025% formic acid and 10 mmol/L ammonium acetate, solvent A) and acetonitrile: methanol (9:1, v/v) solution (solvent B), was delivered at a flow rate was set to 0.4 mL/min. During the period of analysis, all samples were stored at 4 ℃.

The mass spectrometric data was collected using a SCIEX QTRAP 6500+ Mass Spectrometer equipped with an electrospray ionization (ESI) source operating in negative ion mode. The optimal parameters were set as followed: Curtain Gas (CUR) :35, Collision Gas (CAD): Medium, IonSpray Voltage (IS): -4500, Temperature (TEM): 550, Ion Source Gas1 (GS1): 50, Ion Source Gas2 (GS2):50.

The LC-MS raw data were imported into Sciex software OS. All ion fragments were automatically identificated and integrated by using default parameters, besides, all intergration was checked manually. The metabolite concentration of sample was calculated according to linear regression standard curve.

**RNA-seq**

Total RNA from samples was isolated and purified using the TRIzol reagent (Invitrogen, Carlsbad, USA). After purification of poly (A) RNA, reverse transcription, and amplification, cDNA libraries were generated using NEBNext® UltraTM RNA Library Prep Kit for Illumina (NEB, #E7770, USA) and sequenced with 2×150bp paired-end sequencing (PE150) on an illumina Novaseq™ 6000 (Illumina, San Diego, CA).

Raw data of fastq were processed using fastp software (v0.23.1) with default parameters to obtain clean reads by removing adaptor contamination, low-quality bases and undetermined bases. Then, HISAT2[1] software (v2.1.0) was employed to map these clean reads to the Ensembl Sus scrofa 11.1 reference genome (release 105) or the Ensembl GRCh 38.p13 (release 109). The “featureCounts” function in the subread package (v2.0.2) was used to quantify mRNA expression levels. Differentially expressed mRNAs were selected based on log2 FC (|log2 FC| ≥ 1.0) and models were derived from negative binomial distribution (*P* value < 0.05) by DESeq2[2] package (v1.38.3).

Table S1. Primer sequences used for *Cyp8b1*-KO genotyping

| PCR No. | Primer No. | Sequence | Band Size |
| --- | --- | --- | --- |
| PCR 1 | T017736-F1 | AGAGAGGCCGTGAACTCTCAATG | WT:8688bp |
|  | T017736-R1 | CTTCTACAAGGCTAGGAACCCAAGG | KO: 314bp |
| PCR 2 | T017736-F2 | CCGATGAGCTGTTCAGGAAGTTC | WT:439bp |
|  | T017736-R2 | TCCATCACGCTGTCCAACACTG | KO:0bp |

Table S2. Average recovery rates of AFB1 in samples

| Sample (*n* = 3) | Concentration, ng/g | Recovery rate, % |
| --- | --- | --- |
| Liver | 5 | 80.60±5.69 |
|  | 15 | 81.26±5.26 |
|  | 50 | 82.56±7.01 |
| Intestinal content | 5 | 103.17±12.80 |
|  | 15 | 96.54±8.54 |
|  | 50 | 100.24±3.24 |

Table S3. Primer sequences for qPCR analysis

| Target gene | Forward Primer Sequence (5’-3’) | Reverse Primer Sequence (5’-3’) |
| --- | --- | --- |
| For host genes of pig | | |
| *GAPDH* | GTCGGAGTGAACGGATTTGGC | GGAGGTCAATGAAGGGGTCA |
| *CYP8B1* | GCAGGCAAGAAGATCCACCACTAC | TGACCATGAGCAGCACAAAGAGC |
| *CYP27A1* | ACTGAAGACCGCGATGAAAC | CAAAGGCGAATCAGGAAGGG |
| *CYP7A1* | GAAAGAGAGACCACATCTCGG | GAATGGTGTTGGCTTGCGAT |
| *CYP7B1* | GGTCTTATTCCTGCGCACCC | AAATGTGATGTACTTTCCTCCAAGG |
|  |  |  |
| For host genes of mouse | | |
| *GAPDH* | CATCACTGCCACCCAGAAGACTG | ATGCCAGTGAGCTTCCCGTTCAG |
| *CYP8B1* | CATGAAGGCTGTGCGTGAGGAA | CATCACGCTGTCCAACACTGGA |
| *CYP27A1* | TCAGGAGACCATCGGCACCTTT | CCAGTCACTTCCTTGTGCAAGG |
| *CYP7A1* | CACCATTCCTGCAACCTTCTGG | ATGGCATTCCCTCCAGAGCTGA |
| *CYP7B1* | CGGAAATCTTCGATGCTCCAAAG | GCTTGTTCCGAGTCCAAAAGGC |
|  |  |  |
| For bacteria | | |
| Total bacteria | GTGSTGCAYGGYYGTCGTCA | ACGTCRTCCMCNCCTTCCTC |
| *Lactobacillus* | AGCAGTAGGGAATCTTCCA | ATTCCACCGCTACACATG |
| *bsh* | ATGGGCGGACTAGGATTACC | TGCCACTCTCTGTCTGCATC |

Table S4. Dissimilarities of colonic microbiota between piglets in different groups revealed by ANOSIM based on Bray–Curtis distance

| Group 1 | Group 2 | Sample size | Permutations | R-value | *P*-value |
| --- | --- | --- | --- | --- | --- |
| CON | AFB1 | 12 | 999 | 0.052 | 0.276 |
| CON | ABX | 12 | 999 | 0.952 | 0.002 |
| CON | ABX+AFB1 | 12 | 999 | 0.965 | 0.005 |
| AFB1 | ABX | 12 | 999 | 0.787 | 0.002 |
| AFB1 | ABX+AFB1 | 12 | 999 | 0.865 | 0.002 |
| ABX | ABX+AFB1 | 12 | 999 | 0.270 | 0.004 |

Table S5. Dissimilarities of ileal microbiota between piglets in different groups revealed by ANOSIM based on Bray–Curtis distance

| Group 1 | Group 2 | Sample size | Permutations | R-value | *P*-value |
| --- | --- | --- | --- | --- | --- |
| CON | AFB1 | 12 | 999 | 0.207 | 0.090 |
| CON | ABX | 12 | 999 | 0.231 | 0.061 |
| CON | ABX+AFB1 | 12 | 999 | 0.457 | 0.002 |
| AFB1 | ABX | 12 | 999 | 0.361 | 0.005 |
| AFB1 | ABX+AFB1 | 12 | 999 | 0.511 | 0.002 |
| ABX | ABX+AFB1 | 12 | 999 | -0.039 | 0.583 |


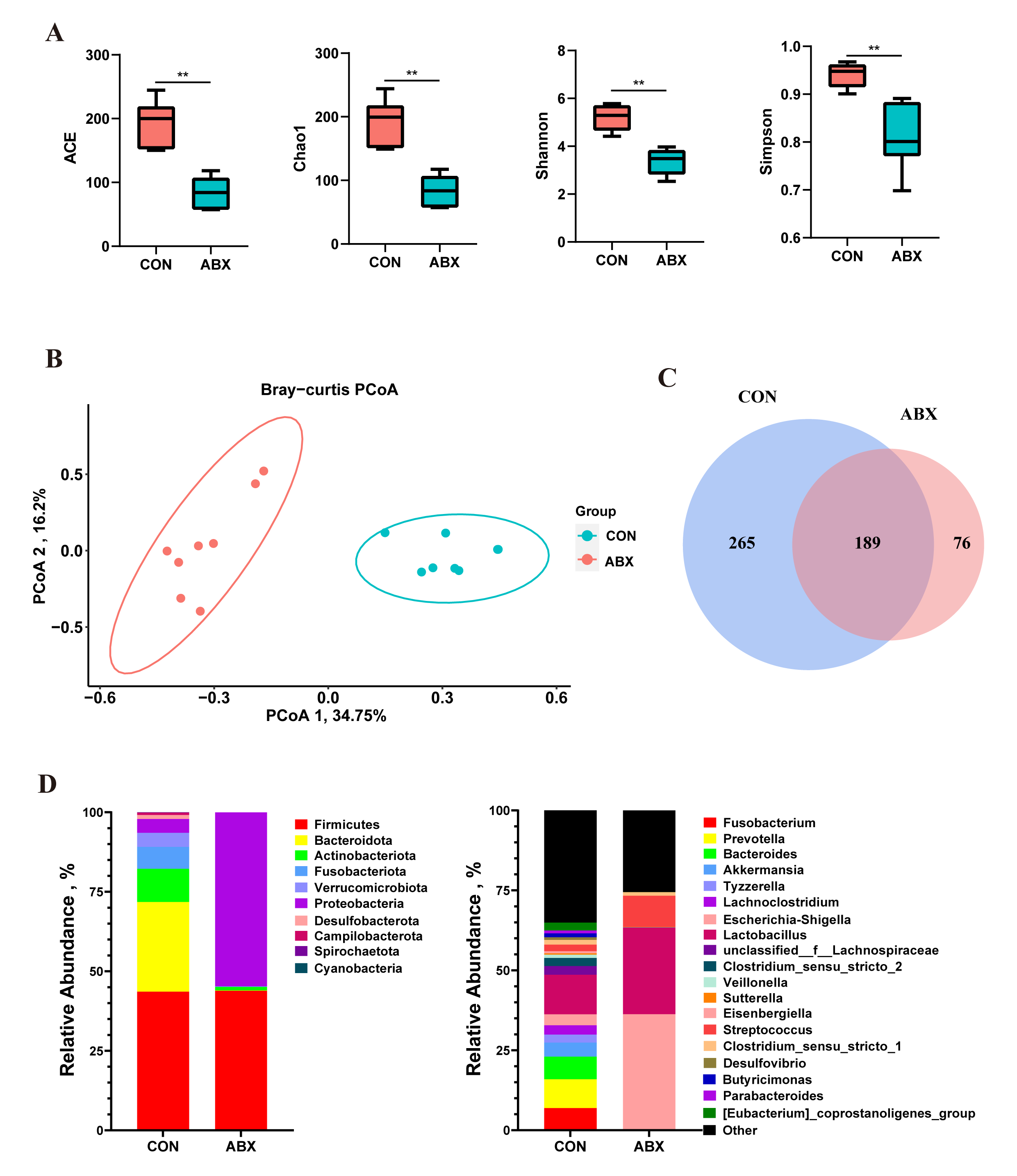


Figure S1. ABX cleared the gut microbiota of piglets on d 3. (A) ACE, Chao1, Shannon and Simpson index in α-diversity analysis (*n* = 8). (B) Principal Coordinates Analysis (PCoA) of β diversity. (C) ASVs Venn diagram. (D) Relative abundance of microbiota at phylum and genus level. Statistical analysis: one-way ANOVA and Tukey’s post-test for the comparison of four groups (***P* < 0.01, **P* < 0.05).


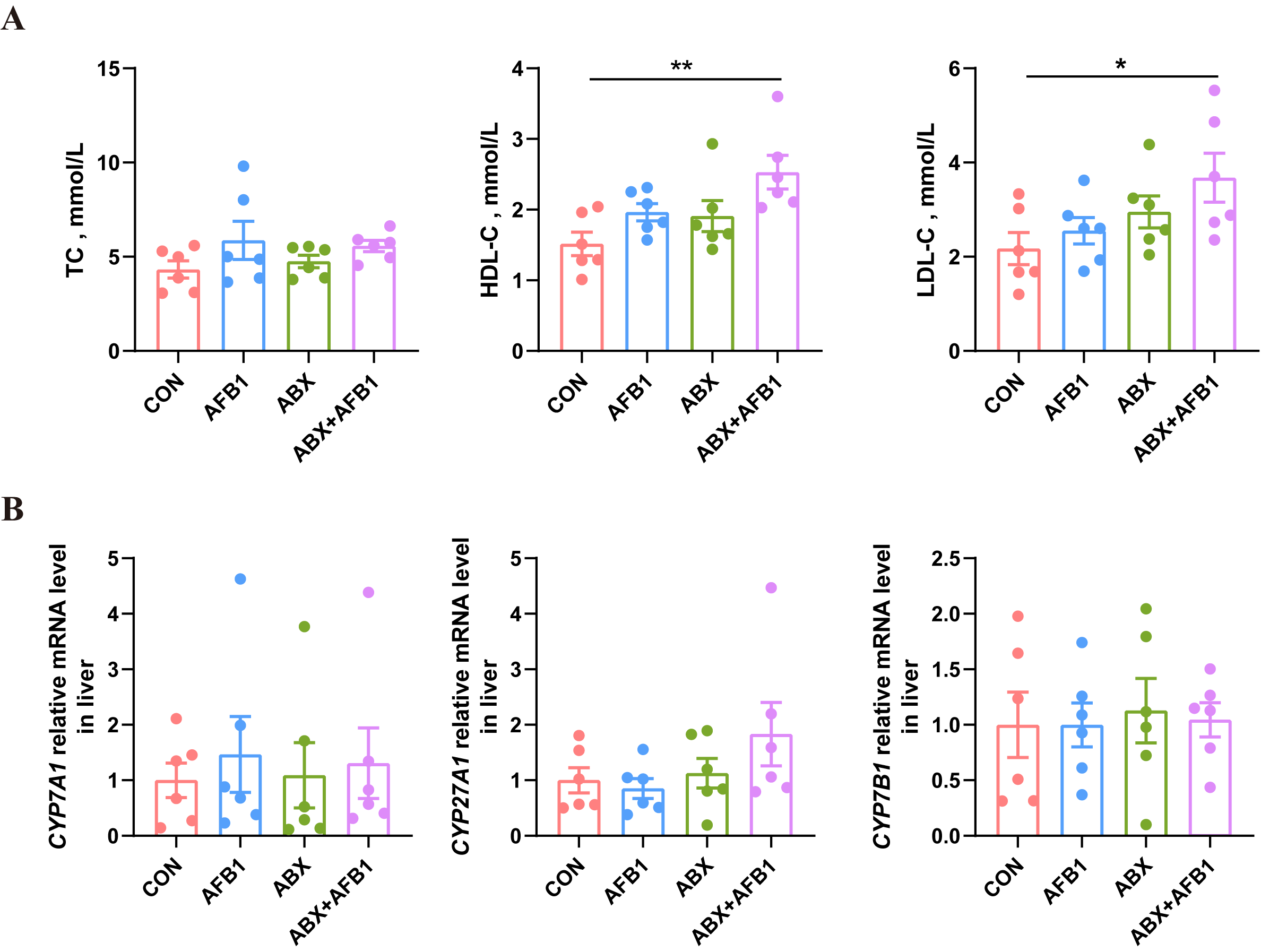


Figure S2. Effect of AFB1 and ABX treatments on piglets. Related to Figure 1 and Figure 4. (A) Levels of TC, HDL-C and LDL-C in the serum of piglets (*n* = 6). (B) Relative mRNA expression of hepatic *CYP7A1*, *CYP27A1* and *CYP7B1* from qPCR (*n* = 6). Statistical analysis: one-way ANOVA and Tukey’s post-test for the comparison of four groups (***P* < 0.01, **P* < 0.05).


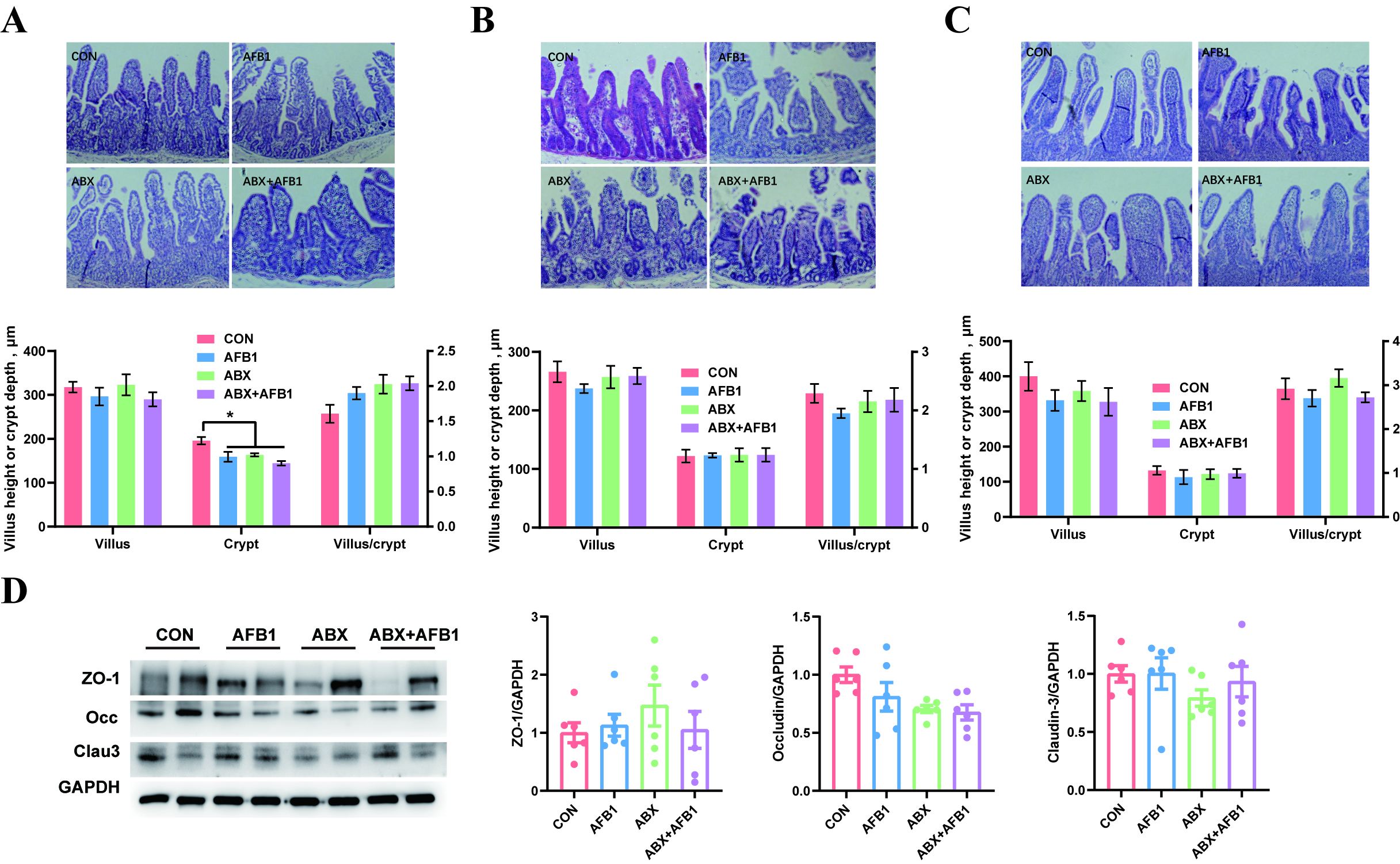


Figure S3. Effect of AFB1 and ABX on the intestinal barrier of piglets. Representative images of duodenum (A), jejunum (B), ileum (C) sections stained with H&E and the statistic of villus height and crypt depth (*n* = 6). (D) Protein expression of colonic tight junction protein (*n* = 6). Statistical analysis: one-way ANOVA and Tukey’s post-test for the comparison of four groups (**P* < 0.05).


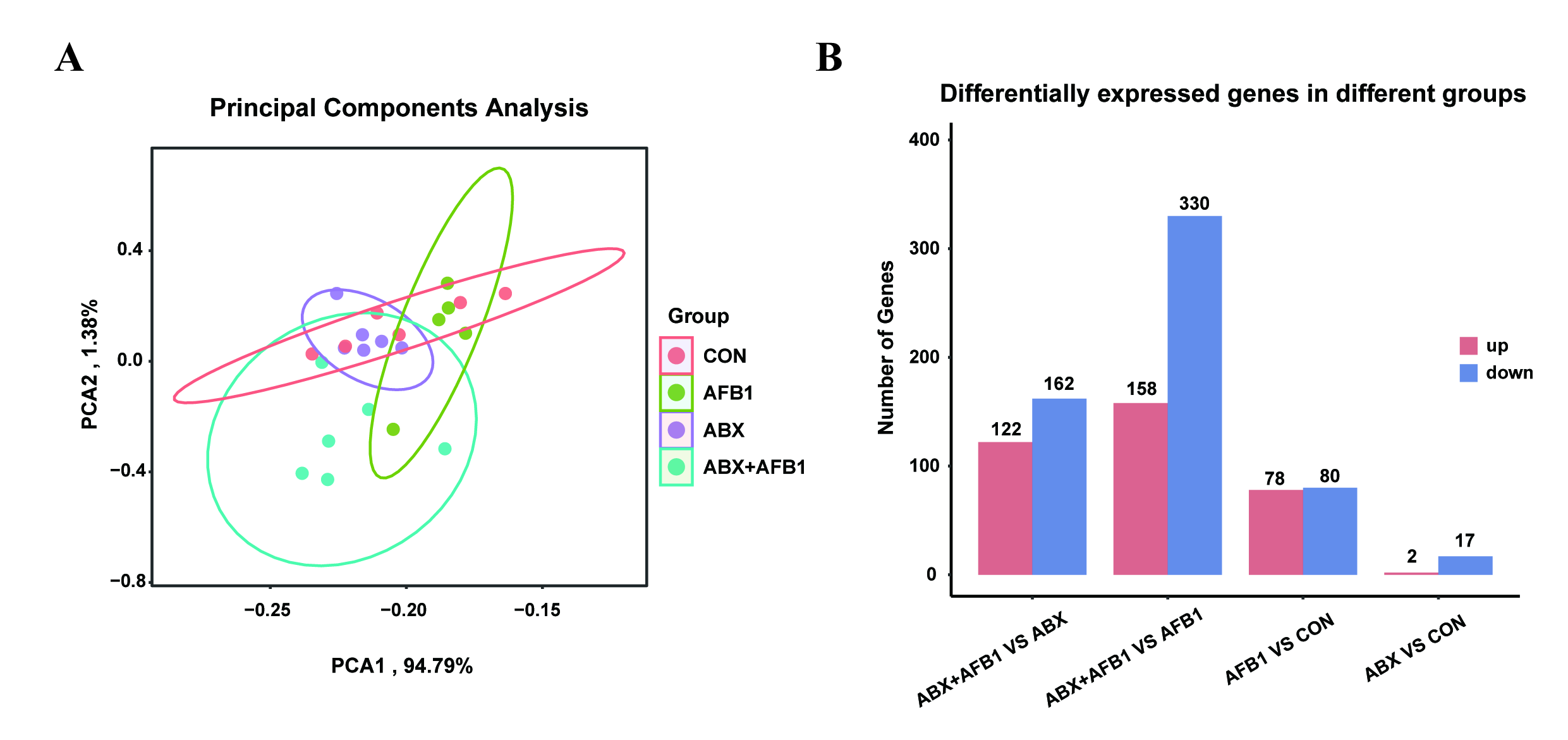


Figure S4. Overview of RNA-seq. (A) Principal Coordinates Analysis (PCoA). (B) Numbers of differentially expressed genes in different groups.


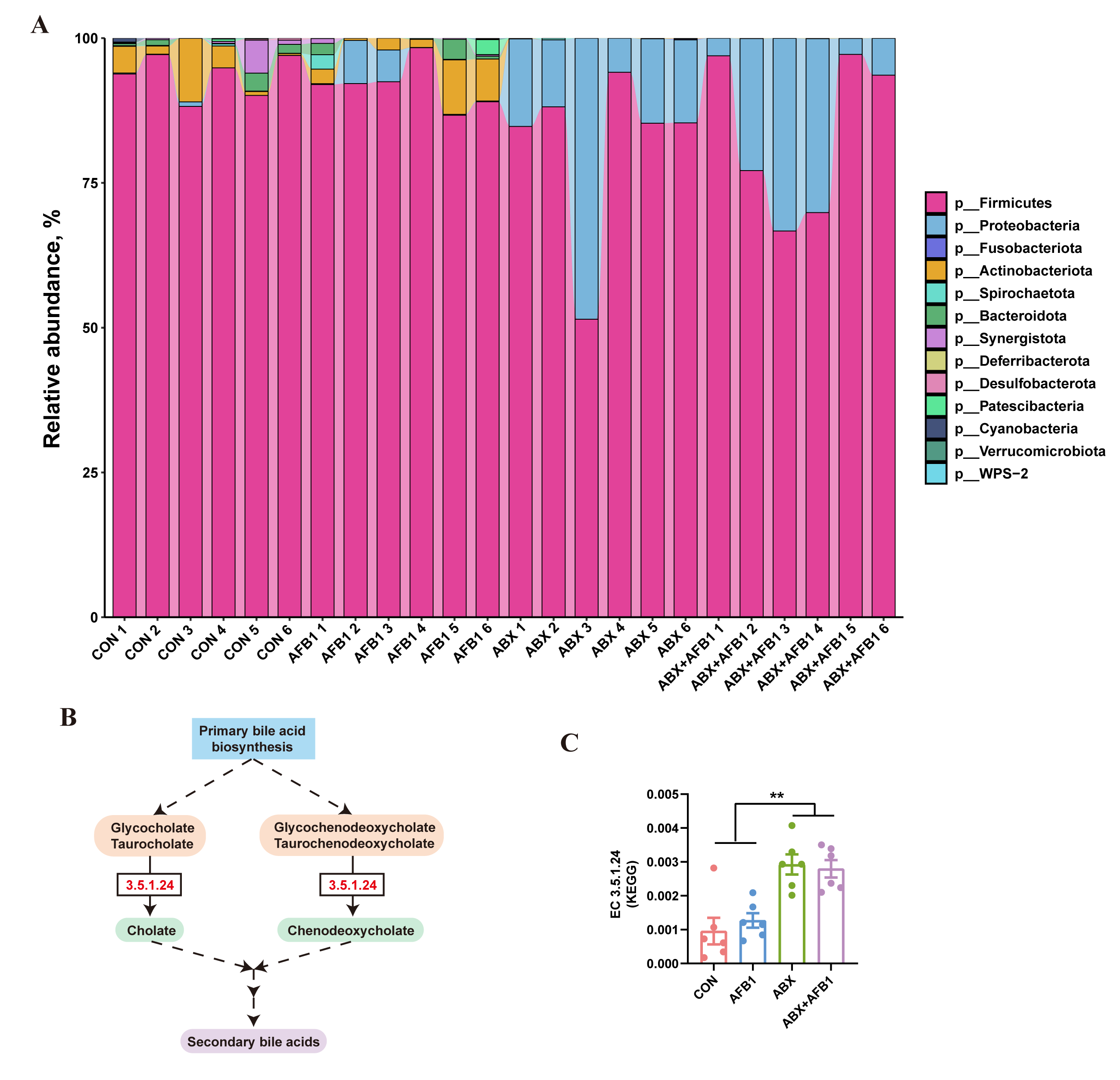


Figure S5. Effect of AFB1 and ABX treatment on colonic microbiota of piglets. (A) Relative abundance of colonic microbiota at phylum level. (B) Simplified secondary bile acid biosynthesis pathway diagram (KEGG). (C) EC 3.5.1.24 (choloylglycine hydrolase) activity prediction of colonic microbiota revealed by PICRUSt2 with KEGG pathway database (*n* = 6). Statistical analysis: one-way ANOVA and Tukey’s post-test for the comparison of four groups (***P* < 0.01).


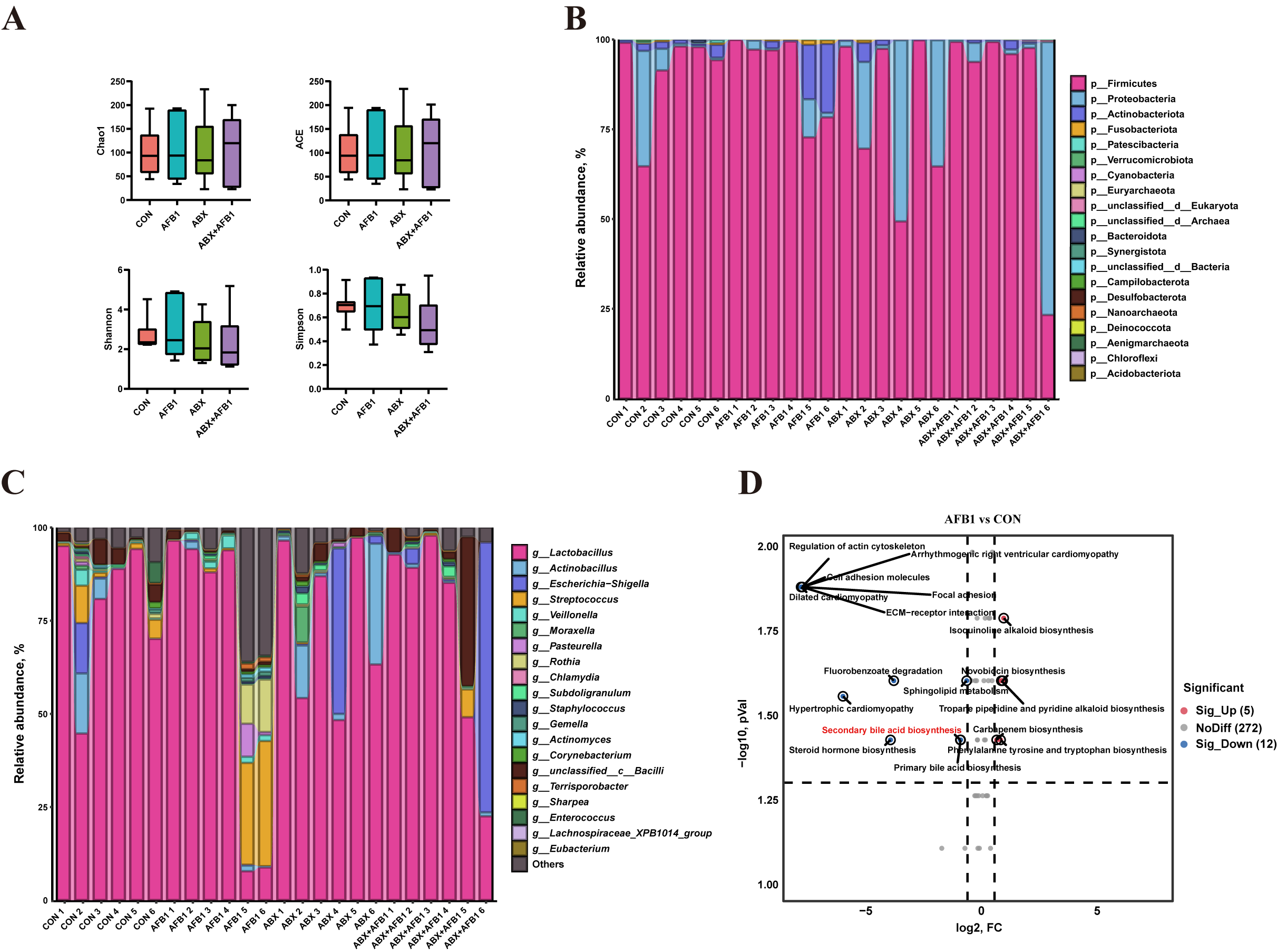


Figure S6. Effect of AFB1 and ABX treatment on ileal microbiota of piglets. (A) ACE, Chao1, Shannon and Simpson index in α-diversity analysis (*n* = 6). (B) Relative abundance of microbiota at phylum level. (C) Relative abundance of microbiota at genus level. (D) Volcano plots of significantly changed KEGG pathway in the CON and AFB1 groups (PICRUSt2, *P* < 0.05, FC >1.5).


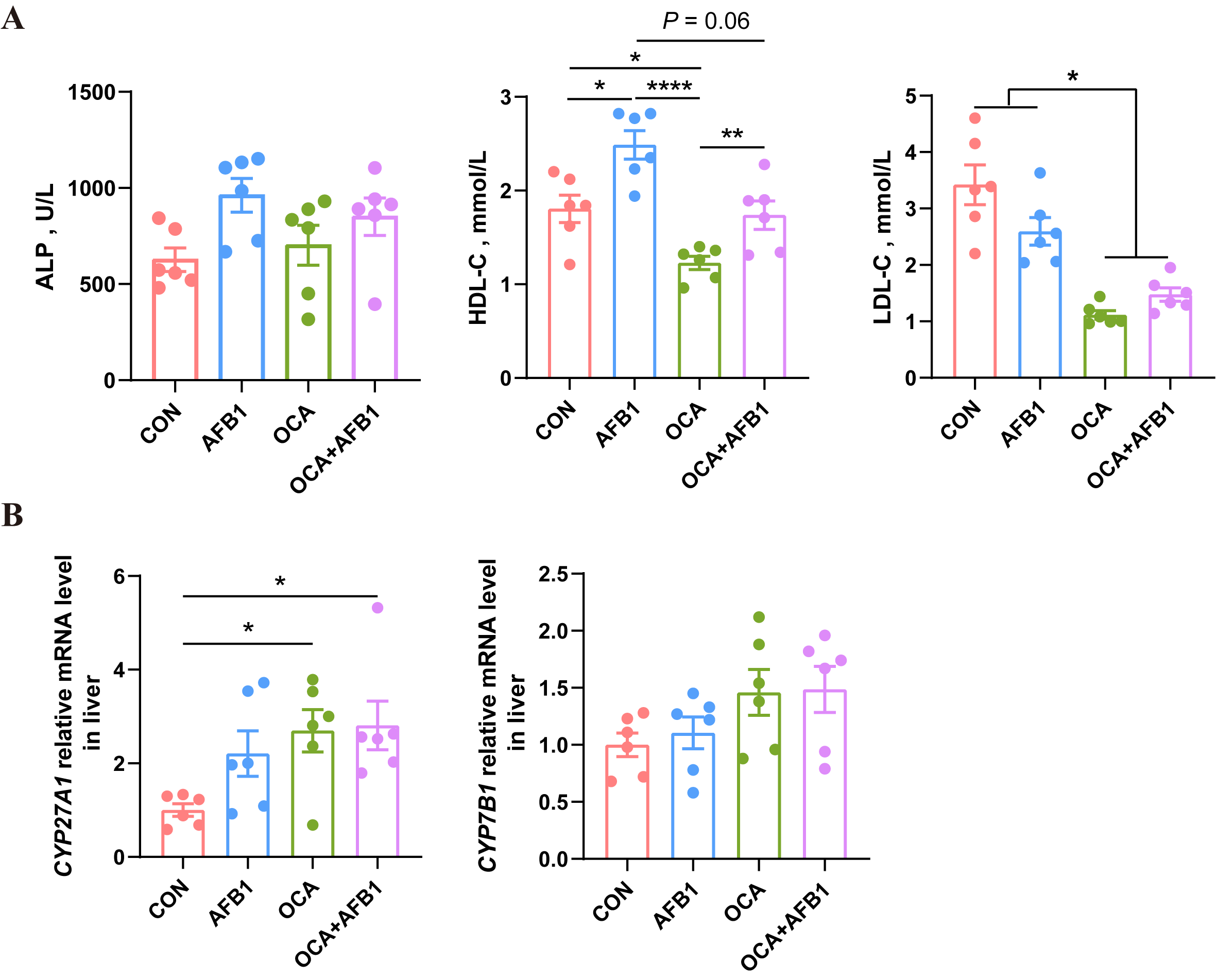
Figure S7. Effect of AFB1 and OCA treatments on piglets. Related to Figure 5 and Figure 6. (A) Levels of ALP, HDL-C and LDL-C in the serum of piglets (*n* = 6). (B) Relative mRNA expression of hepatic *CYP27A1* and *CYP7B1* (*n* = 6). Statistical analysis: one-way ANOVA and Tukey’s post-test for the comparison of four groups (*****P* < 0.0001; ***P* < 0.01; **P* < 0.05).


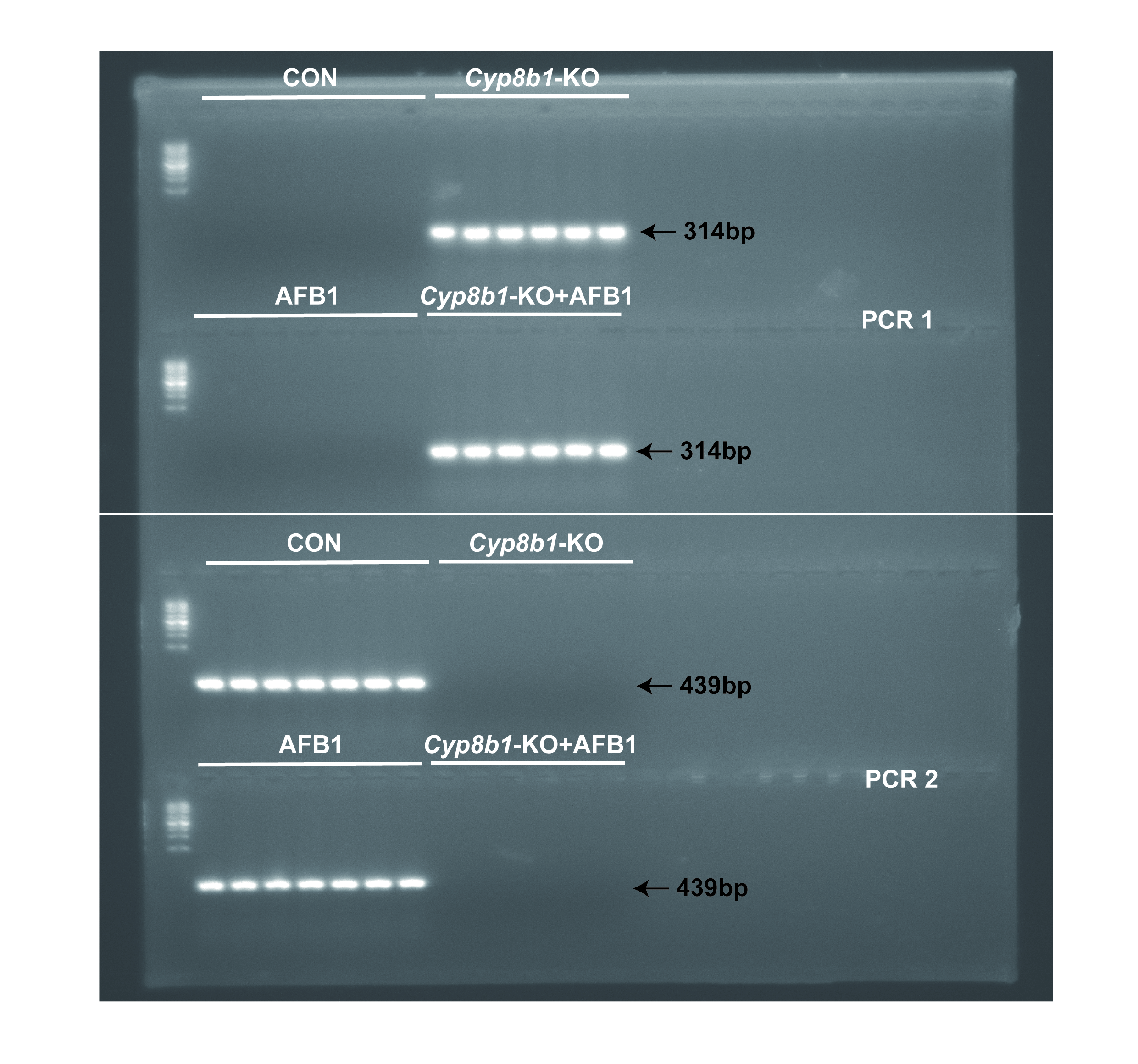


Figure S8. Results of *Cyp8b1*-KO mice genotyping.


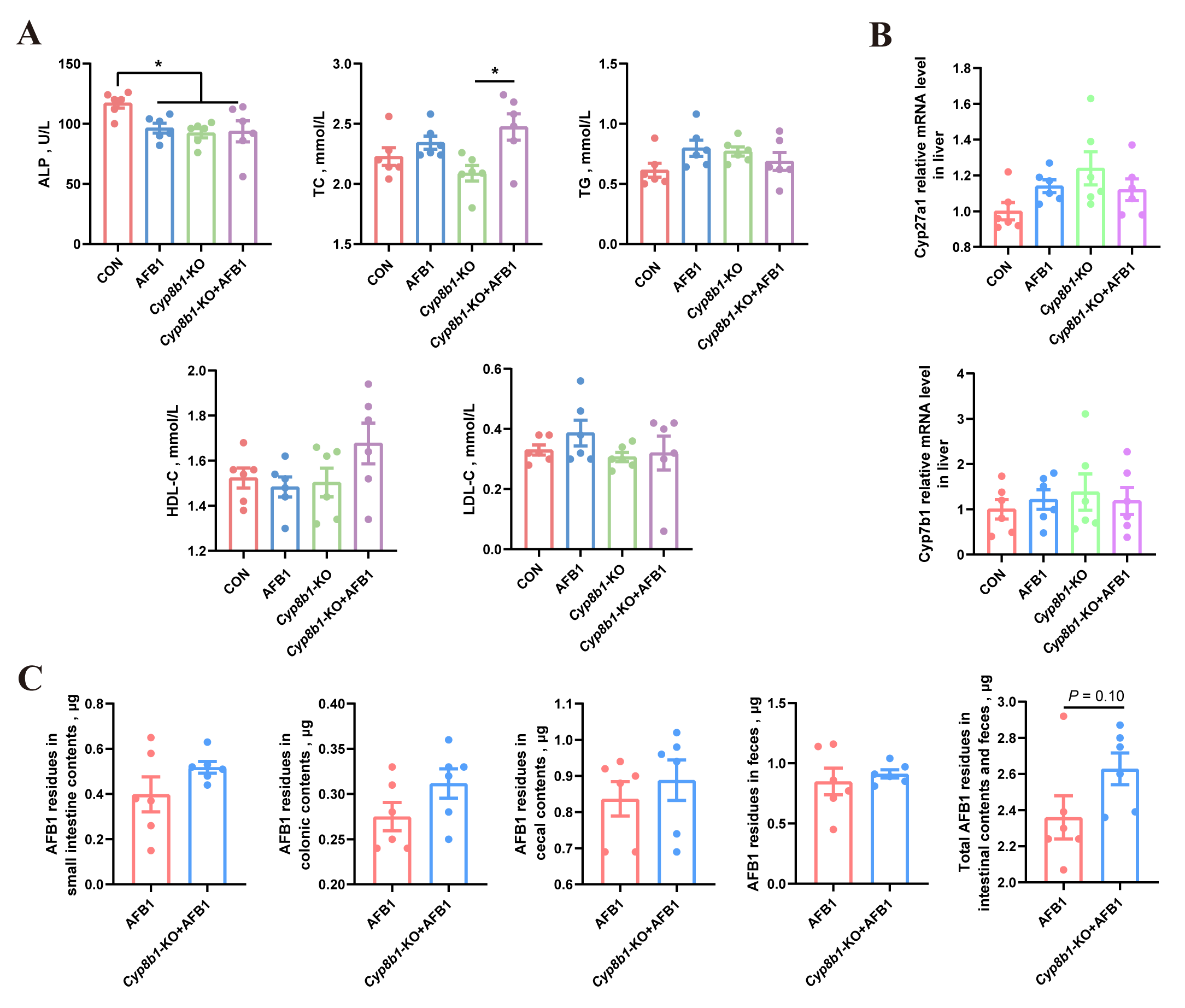


Figure S9. Effect of AFB1 treatment on WT mice and *Cyp8b1*-KO mice. Related to Figure 7. (A) Levels of ALP, TC, TG, HDL-C and LDL-C in the serum of mice (*n* = 6). (B) Relative mRNA expression of hepatic *Cyp27a1* and *Cyp7b1* (*n* = 6). (C) Amount of AFB1 residues in the contents of each intestinal segment and feces of mice (*n* = 6). Statistical analysis: one-way ANOVA and Tukey’s post-test for the comparison of four groups (**P* < 0.05).
