# Supplementary material for: Gut microbiota-mediated bile acid transformations regulate the transport of aflatoxin B1 from the intestine to the liver in piglets
Source: J Anim Sci Biotechnol. 2025 Mar 8;16:38. doi: 10.1186/s40104-025-01169-x (PMC11889867; doi:10.1186/s40104-025-01169-x)
Supplement: Supplementary file 2 — Additional file 2: Table S6. EC 3.5.1.24 (choloylglycine hydrolase) activity prediction of ASVs revealed by PICRUSt2 with KEGG pathway database. [file 40104_2025_1169_MOESM2_ESM.docx]

Table S6. EC 3.5.1.24 (choloylglycine hydrolase) activity prediction of ASVs revealed by PICRUSt2 with KEGG pathway database.

| ASV | Categorical | Relative abundance (%) | EC:3.5.1.24 |
| --- | --- | --- | --- |
| ASV504 | d__Bacteria; p__Firmicutes; c__Bacilli; o__Lactobacillales; f__Lactobacillaceae; g__Lactobacillus | 0.0048 | 4 |
| ASV448 | d__Bacteria; p__Firmicutes; c__Bacilli; o__Lactobacillales; f__Lactobacillaceae; g__Lactobacillus | 0.0037 | 4 |
| ASV94 | d__Bacteria; p__Firmicutes; c__Bacilli; o__Lactobacillales; f__Lactobacillaceae; g__Lactobacillus | 0.0073 | 4 |
| ASV57 | d__Bacteria; p__Firmicutes; c__Bacilli; o__Lactobacillales; f__Lactobacillaceae; g__Lactobacillus | 10.4346 | 3 |
| ASV2 | d__Bacteria; p__Firmicutes; c__Bacilli; o__Lactobacillales; f__Lactobacillaceae; g__Lactobacillus | 10.6071 | 3 |
| ASV329 | d__Bacteria; p__Firmicutes; c__Bacilli; o__Lactobacillales; f__Lactobacillaceae; g__Lactobacillus | 0.0000 | 3 |
| ASV147 | d__Bacteria; p__Firmicutes; c__Clostridia; o__Clostridiales; f__Clostridiaceae; g__Clostridium_sensu_stricto_1 | 0.0176 | 3 |
| ASV262 | d__Bacteria; p__Firmicutes; c__Bacilli; o__Lactobacillales; f__Lactobacillaceae; g__Lactobacillus | 0.0016 | 2 |
| ASV128 | d__Bacteria; p__Firmicutes; c__Bacilli; o__Lactobacillales; f__Enterococcaceae; g__Enterococcus | 0.0102 | 2 |
| ASV345 | d__Bacteria; p__Firmicutes; c__Bacilli; o__Lactobacillales | 0.0019 | 2 |
| ASV60 | d__Bacteria; p__Firmicutes; c__Clostridia; o__Eubacteriales; f__Eubacteriaceae; g__Eubacterium | 0.0388 | 2 |
| ASV349 | d__Bacteria; p__Firmicutes; c__Bacilli; o__Lactobacillales; f__Lactobacillaceae; g__Lactobacillus | 0.0018 | 2 |
| ASV359 | d__Bacteria; p__Bacteroidota; c__Bacteroidia; o__Bacteroidales; f__Bacteroidaceae; g__Bacteroides | 0.0016 | 2 |
| ASV250 | d__Bacteria; p__Firmicutes; c__Clostridia; o__Lachnospirales; f__Lachnospiraceae; g__Lachnoclostridium | 0.0016 | 2 |
| ASV87 | d__Bacteria; p__Firmicutes; c__Clostridia; o__Clostridiales; f__Clostridiaceae; g__Clostridium_sensu_stricto_1 | 0.0213 | 2 |
| ASV470 | d__Bacteria; p__Firmicutes; c__Bacilli; o__Lactobacillales; f__Lactobacillaceae; g__Lactobacillus | 0.0022 | 2 |
| ASV181 | d__Bacteria; p__Firmicutes; c__Bacilli; o__Lactobacillales; f__Lactobacillaceae; g__Lactobacillus | 0.0350 | 2 |
| ASV307 | d__Bacteria; p__Proteobacteria; c__Alphaproteobacteria; o__Rhizobiales; f__Rhizobiaceae; g__Ochrobactrum | 0.0000 | 2 |
| ASV475 | d__Bacteria; p__Firmicutes; c__Bacilli; o__Lactobacillales; f__Lactobacillaceae; g__Lactobacillus | 2.5995 | 2 |
| ASV21 | d__Bacteria; p__Firmicutes; c__Bacilli; o__Lactobacillales; f__Lactobacillaceae; g__Lactobacillus | 2.5153 | 2 |
| ASV65 | d__Bacteria; p__Firmicutes; c__Bacilli; o__Lactobacillales; f__Lactobacillaceae; g__Lactobacillus | 0.0328 | 2 |
| ASV223 | d__Bacteria; p__Bacteroidota; c__Bacteroidia; o__Bacteroidales; f__Muribaculaceae; g__Muribaculaceae | 0.0016 | 2 |
| ASV13 | d__Bacteria; p__Firmicutes; c__Bacilli; o__Lactobacillales; f__Lactobacillaceae; g__Lactobacillus | 10.5286 | 2 |
| ASV351 | d__Bacteria; p__Firmicutes; c__Bacilli; o__Lactobacillales; f__Lactobacillaceae; g__Lactobacillus | 0.0254 | 2 |
| ASV411 | d__Bacteria; p__Firmicutes; c__Bacilli; o__Lactobacillales; f__Enterococcaceae; g__Enterococcus | 0.0041 | 2 |
| ASV480 | d__Bacteria; p__Bacteroidota; c__Bacteroidia; o__Bacteroidales; f__Muribaculaceae; g__Muribaculaceae | 0.0022 | 2 |
| ASV498 | d__Bacteria; p__Firmicutes; c__Bacilli; o__Lactobacillales; f__Lactobacillaceae; g__Lactobacillus | 0.0177 | 2 |
| ASV243 | d__Bacteria; p__Firmicutes; c__Bacilli; o__Lactobacillales; f__Lactobacillaceae; g__Lactobacillus | 0.0419 | 2 |
| ASV434 | d__Bacteria; p__Firmicutes; c__Bacilli; o__Lactobacillales; f__Enterococcaceae; g__Enterococcus | 0.0018 | 2 |
| ASV1 | d__Bacteria; p__Firmicutes; c__Bacilli; o__Lactobacillales; f__Lactobacillaceae; g__Lactobacillus | 20.3617 | 2 |
| ASV449 | d__Bacteria; p__Firmicutes; c__Bacilli; o__Lactobacillales; f__Lactobacillaceae; g__Lactobacillus | 0.0016 | 2 |
| ASV353 | d__Bacteria; p__Firmicutes; c__Bacilli; o__Lactobacillales; f__Lactobacillaceae; g__Lactobacillus | 0.0156 | 2 |
| ASV486 | d__Bacteria; p__Bacteroidota; c__Bacteroidia; o__Bacteroidales; f__Bacteroidaceae; g__Bacteroides | 0.0015 | 2 |
| ASV189 | d__Bacteria; p__Firmicutes; c__Clostridia; o__Lachnospirales; f__Lachnospiraceae; g__Howardella | 0.1946 | 2 |
| ASV290 | d__Bacteria; p__Firmicutes; c__Bacilli; o__Lactobacillales; f__Lactobacillaceae; g__Lactobacillus | 0.0016 | 2 |
| ASV185 | d__Bacteria; p__Firmicutes; c__Clostridia; o__Oscillospirales; f__Ruminococcaceae; g__UBA1819 | 0.0012 | 2 |
| ASV454 | d__Bacteria; p__Firmicutes; c__Bacilli; o__Lactobacillales; f__Lactobacillaceae; g__Lactobacillus | 0.0016 | 1 |
| ASV318 | d__Bacteria; p__Firmicutes; c__Bacilli; o__Lactobacillales; f__Streptococcaceae; g__Lactococcus | 0.0000 | 1 |
| ASV90 | d__Bacteria; p__Actinobacteriota; c__Coriobacteriia; o__Coriobacteriales; f__Coriobacteriaceae; g__Collinsella | 0.0891 | 1 |
| ASV481 | d__Bacteria; p__Bacteroidota; c__Bacteroidia; o__Bacteroidales; f__Marinifilaceae; g__Odoribacter | 0.0022 | 1 |
| ASV168 | d__Bacteria; p__Firmicutes; c__Bacilli; o__Staphylococcales; f__Staphylococcaceae; g__Staphylococcus | 0.0034 | 1 |
| ASV228 | d__Bacteria; p__Firmicutes; c__Clostridia; o__Clostridiales; f__Clostridiaceae; g__Clostridium_sensu_stricto_1 | 0.0012 | 1 |
| ASV69 | d__Bacteria; p__Firmicutes; c__Bacilli; o__Lactobacillales; f__Lactobacillaceae; g__Lactobacillus | 1.1751 | 1 |
| ASV150 | d__Bacteria; p__Firmicutes; c__Clostridia; o__Peptostreptococcales-Tissierellales; f__Anaerovoracaceae; g__[Eubacterium]_nodatum_group | 0.0027 | 1 |
| ASV118 | d__Bacteria; p__Firmicutes; c__Clostridia; o__Clostridiales; f__Clostridiaceae; g__Clostridium_sensu_stricto_1 | 0.0099 | 1 |
| ASV368 | d__Bacteria; p__Firmicutes; c__Clostridia; o__Peptostreptococcales-Tissierellales; f__Peptostreptococcaceae | 0.0054 | 1 |
| ASV97 | d__Bacteria; p__Firmicutes; c__Clostridia; o__Lachnospirales; f__Lachnospiraceae; g__Blautia | 0.0257 | 1 |
| ASV367 | d__Bacteria; p__Firmicutes; c__Bacilli; o__Lactobacillales; f__Lactobacillaceae; g__Lactobacillus | 0.0033 | 1 |
| ASV488 | d__Bacteria; p__Bacteroidota; c__Bacteroidia; o__Bacteroidales; f__Bacteroidaceae; g__Bacteroides | 0.0014 | 1 |
| ASV385 | d__Bacteria; p__Firmicutes; c__Bacilli; o__Lactobacillales; f__Carnobacteriaceae; g__Granulicatella | 0.5362 | 1 |
| ASV81 | d__Bacteria; p__Firmicutes; c__Clostridia; o__Lachnospirales; f__Lachnospiraceae; g__Blautia | 0.0268 | 1 |
| ASV261 | d__Bacteria; p__Firmicutes; c__Bacilli; o__Lactobacillales; f__Lactobacillaceae; g__Lactobacillus | 0.0025 | 1 |
| ASV53 | d__Bacteria; p__Firmicutes; c__Clostridia; o__Clostridiales; f__Clostridiaceae; g__Clostridium_sensu_stricto_1 | 0.0097 | 1 |
| ASV67 | d__Bacteria; p__Firmicutes; c__Bacilli; o__Lactobacillales; f__Lactobacillaceae; g__Lactobacillus | 10.9125 | 1 |
| ASV107 | d__Bacteria; p__Actinobacteriota; c__Coriobacteriia; o__Coriobacteriales; f__Atopobiaceae; g__Olsenella | 0.0067 | 1 |
| ASV80 | d__Bacteria; p__Actinobacteriota; c__Coriobacteriia; o__Coriobacteriales; f__Atopobiaceae; g__Olsenella | 0.0253 | 1 |
| ASV109 | d__Bacteria; p__Actinobacteriota; c__Coriobacteriia; o__Coriobacteriales; f__Atopobiaceae; g__Olsenella | 0.0089 | 1 |
| ASV332 | d__Bacteria; p__Firmicutes; c__Bacilli; o__Lactobacillales; f__Enterococcaceae; g__Enterococcus | 0.0000 | 1 |
| ASV70 | d__Bacteria; p__Firmicutes; c__Bacilli; o__Lactobacillales; f__Lactobacillaceae; g__Lactobacillus | 1.5355 | 1 |
| ASV477 | d__Bacteria; p__Bacteroidota; c__Bacteroidia; o__Bacteroidales; f__Bacteroidaceae; g__Bacteroides | 0.0033 | 1 |
| ASV28 | d__Bacteria; p__Firmicutes; c__Bacilli; o__Erysipelotrichales; f__Erysipelotrichaceae; g__Turicibacter | 0.0292 | 1 |
| ASV371 | d__Bacteria; p__Firmicutes; c__Bacilli; o__Erysipelotrichales; f__Erysipelotrichaceae; g__Faecalibaculum | 0.0025 | 1 |
| ASV76 | d__Bacteria; p__Actinobacteriota; c__Actinobacteria; o__Bifidobacteriales; f__Bifidobacteriaceae; g__Bifidobacterium | 0.1061 | 1 |
| ASV56 | d__Bacteria; p__Firmicutes; c__Bacilli; o__Erysipelotrichales; f__Erysipelatoclostridiaceae; g__Catenibacterium | 0.0660 | 1 |
| ASV364 | d__Bacteria; p__Firmicutes; c__Bacilli; o__Lactobacillales; f__Lactobacillaceae; g__Lactobacillus | 0.1066 | 1 |
| ASV476 | d__Bacteria; p__Bacteroidota; c__Bacteroidia; o__Bacteroidales; f__Bacteroidaceae; g__Bacteroides | 0.0033 | 1 |
| ASV278 | d__Bacteria; p__Firmicutes; c__Bacilli; o__Lactobacillales; f__Lactobacillaceae; g__Lactobacillus | 0.4048 | 1 |
| ASV313 | d__Bacteria; p__Firmicutes; c__Bacilli; o__Staphylococcales; f__Staphylococcaceae; g__Staphylococcus | 0.0001 | 1 |
| ASV356 | d__Bacteria; p__Firmicutes; c__Clostridia; o__Lachnospirales; f__Lachnospiraceae | 0.0000 | 1 |
| ASV260 | d__Bacteria; p__Firmicutes; c__Clostridia; o__Oscillospirales; f__Oscillospiraceae; g__Colidextribacter | 0.0018 | 1 |
| ASV58 | d__Bacteria; p__Firmicutes; c__Bacilli; o__Lactobacillales; f__Lactobacillaceae; g__Lactobacillus | 0.0019 | 1 |
| ASV145 | d__Bacteria; p__Firmicutes; c__Bacilli; o__Erysipelotrichales; f__Erysipelotrichaceae; g__Faecalicoccus | 0.0030 | 1 |
| ASV64 | d__Bacteria; p__Firmicutes; c__Bacilli; o__Staphylococcales; f__Staphylococcaceae; g__Staphylococcus | 0.0029 | 1 |
| ASV497 | d__Bacteria; p__Firmicutes; c__Bacilli; o__Lactobacillales; f__Streptococcaceae; g__Streptococcus | 0.0018 | 1 |
| ASV129 | d__Bacteria; p__Firmicutes; c__Clostridia; o__Lachnospirales; f__Lachnospiraceae; g__Syntrophococcus | 0.0034 | 1 |
| ASV372 | d__Bacteria; p__Firmicutes; c__Bacilli; o__Lactobacillales | 0.0016 | 1 |
| ASV315 | d__Bacteria; p__Proteobacteria; c__Alphaproteobacteria; o__Rhizobiales; f__Rhizobiaceae; g__Aureimonas | 0.0000 | 1 |
| ASV155 | d__Bacteria; p__Firmicutes; c__Clostridia; o__Peptostreptococcales-Tissierellales; f__Anaerovoracaceae; g__[Eubacterium]_nodatum_group | 0.0015 | 1 |
| ASV86 | d__Bacteria; p__Firmicutes; c__Bacilli; o__Erysipelotrichales; f__Erysipelotrichaceae; g__Holdemanella | 0.0321 | 1 |
| ASV51 | d__Bacteria; p__Firmicutes; c__Clostridia; o__Peptostreptococcales-Tissierellales; f__Peptostreptococcaceae; g__Terrisporobacter | 0.0041 | 1 |
| ASV215 | d__Bacteria; p__Firmicutes; c__Clostridia; o__Clostridiales; f__Clostridiaceae; g__Clostridium_sensu_stricto_1 | 0.0025 | 1 |
| ASV237 | d__Bacteria; p__Firmicutes; c__Bacilli; o__Lactobacillales; f__Streptococcaceae; g__Streptococcus | 0.0012 | 1 |
| ASV49 | d__Bacteria; p__Actinobacteriota; c__Actinobacteria; o__Corynebacteriales; f__Dietziaceae; g__Dietzia | 0.0056 | 1 |
| ASV464 | d__Bacteria; p__Firmicutes; c__Bacilli; o__Lactobacillales; f__Enterococcaceae; g__Enterococcus | 0.0015 | 1 |
| ASV121 | d__Bacteria; p__Actinobacteriota; c__Coriobacteriia; o__Coriobacteriales; f__Eggerthellaceae; g__Enterorhabdus | 0.0051 | 1 |
| ASV244 | d__Bacteria; p__Firmicutes; c__Bacilli; o__Lactobacillales; f__Lactobacillaceae; g__Lactobacillus | 0.0104 | 1 |
| ASV116 | d__Bacteria; p__Firmicutes; c__Clostridia; o__Lachnospirales; f__Lachnospiraceae; g__Agathobacter | 0.0084 | 1 |
| ASV34 | d__Bacteria; p__Firmicutes; c__Bacilli; o__Staphylococcales; f__Staphylococcaceae; g__Staphylococcus | 0.0043 | 1 |
| ASV247 | d__Bacteria; p__Firmicutes; c__Bacilli; o__Lactobacillales; f__Lactobacillaceae; g__Lactobacillus | 0.0026 | 1 |
| ASV204 | d__Bacteria; p__Firmicutes; c__Bacilli; o__Erysipelotrichales; f__Erysipelatoclostridiaceae; g__Sharpea | 0.0064 | 1 |
| ASV316 | d__Bacteria; p__Firmicutes; c__Bacilli; o__Lactobacillales; f__Lactobacillaceae; g__Lactobacillus | 0.0000 | 1 |
| ASV124 | d__Bacteria; p__Firmicutes; c__Bacilli; o__Staphylococcales; f__Staphylococcaceae; g__Staphylococcus | 0.0062 | 1 |
| ASV148 | d__Bacteria; p__Firmicutes; c__Clostridia; o__Oscillospirales; f__Oscillospiraceae; g__uncultured | 0.0019 | 1 |
| ASV83 | d__Bacteria; p__Firmicutes; c__Bacilli; o__Erysipelotrichales; f__Erysipelotrichaceae; g__Catenisphaera | 0.0220 | 1 |
| ASV11 | d__Bacteria; p__Firmicutes; c__Bacilli; o__Staphylococcales; f__Staphylococcaceae; g__Staphylococcus | 0.0668 | 1 |
| ASV350 | d__Bacteria; p__Firmicutes; c__Clostridia; o__Lachnospirales; f__Lachnospiraceae; g__Acetitomaculum | 0.0081 | 1 |
| ASV382 | d__Bacteria; p__Firmicutes; c__Clostridia; o__Oscillospirales; f__Ruminococcaceae; g__Subdoligranulum | 0.0034 | 1 |
| ASV43 | d__Bacteria; p__Firmicutes; c__Clostridia; o__Peptostreptococcales-Tissierellales; f__Anaerovoracaceae; g__[Eubacterium]_nodatum_group | 0.0601 | 1 |
| ASV340 | d__Bacteria; p__Firmicutes; c__Bacilli; o__Staphylococcales; f__Staphylococcaceae; g__Staphylococcus | 0.0014 | 1 |
| ASV4 | d__Bacteria; p__Firmicutes; c__Bacilli; o__Lactobacillales; f__Lactobacillaceae; g__Lactobacillus | 0.6867 | 1 |
| ASV419 | d__Bacteria; p__Firmicutes; c__Bacilli; o__Staphylococcales; f__Staphylococcaceae; g__Staphylococcus | 0.0032 | 1 |
| ASV255 | d__Bacteria; p__Firmicutes; c__Bacilli; o__Lactobacillales; f__Lactobacillaceae; g__Lactobacillus | 0.0014 | 1 |
| ASV127 | d__Bacteria; p__Firmicutes; c__Clostridia; o__Lachnospirales; f__Lachnospiraceae | 0.0051 | 1 |
| ASV366 | d__Bacteria; p__Firmicutes; c__Bacilli; o__Lactobacillales; f__Lactobacillaceae; g__Lactobacillus | 0.0501 | 1 |
| ASV6 | d__Bacteria; p__Firmicutes; c__Clostridia; o__Oscillospirales; f__Ruminococcaceae; g__Subdoligranulum | 0.7623 | 1 |
| ASV117 | d__Bacteria; p__Firmicutes; c__Clostridia; o__Lachnospirales; f__Lachnospiraceae | 0.0054 | 1 |
| ASV214 | d__Bacteria; p__Firmicutes; c__Bacilli; o__Erysipelotrichales; f__Erysipelotrichaceae; g__Solobacterium | 0.1667 | 1 |
| ASV71 | d__Bacteria; p__Actinobacteriota; c__Coriobacteriia; o__Coriobacteriales; f__Coriobacteriaceae; g__Collinsella | 0.0408 | 1 |
| ASV292 | d__Bacteria; p__Firmicutes; c__Bacilli; o__Erysipelotrichales; f__Erysipelotrichaceae; g__Holdemanella | 0.0014 | 1 |
| ASV392 | d__Bacteria; p__Firmicutes; c__Bacilli; o__Lactobacillales; f__Enterococcaceae; g__Enterococcus | 0.0412 | 1 |
| ASV412 | d__Bacteria; p__Firmicutes; c__Bacilli; o__Lactobacillales | 0.0088 | 1 |
| ASV159 | d__Bacteria; p__Firmicutes; c__Clostridia; o__Lachnospirales; f__Lachnospiraceae; g__Marvinbryantia | 0.0022 | 1 |
| ASV151 | d__Bacteria; p__Firmicutes; c__Bacilli; o__Lactobacillales; f__Leuconostocaceae; g__Weissella | 0.0047 | 1 |
| ASV478 | d__Bacteria; p__Bacteroidota; c__Bacteroidia; o__Bacteroidales; f__Bacteroidaceae; g__Bacteroides | 0.0027 | 1 |
| ASV304 | d__Bacteria; p__Firmicutes; c__Bacilli; o__Staphylococcales; f__Staphylococcaceae; g__Staphylococcus | 0.0000 | 1 |
| ASV300 | d__Bacteria; p__Firmicutes; c__Bacilli; o__Lactobacillales; f__Enterococcaceae; g__Enterococcus | 0.0000 | 1 |
| ASV279 | d__Bacteria; p__Firmicutes; c__Bacilli; o__Lactobacillales; f__Lactobacillaceae; g__Lactobacillus | 0.0043 | 1 |
| ASV291 | d__Bacteria; p__Firmicutes; c__Bacilli; o__Erysipelotrichales; f__Erysipelotrichaceae; g__Turicibacter | 0.0014 | 1 |
| ASV192 | d__Bacteria; p__Actinobacteriota; c__Coriobacteriia; o__Coriobacteriales; f__Eggerthellaceae; g__DNF00809 | 0.0305 | 1 |
| ASV153 | d__Bacteria; p__Firmicutes; c__Clostridia; o__Clostridiales; f__Clostridiaceae; g__Clostridium_sensu_stricto_1 | 0.0196 | 1 |
| ASV467 | d__Bacteria; p__Actinobacteriota; c__Actinobacteria; o__Bifidobacteriales; f__Bifidobacteriaceae; g__Bifidobacterium | 0.0019 | 1 |
| ASV20 | d__Bacteria; p__Firmicutes; c__Clostridia; o__Peptostreptococcales-Tissierellales; f__Peptostreptococcaceae; g__Romboutsia | 1.7665 | 1 |
| ASV61 | d__Bacteria; p__Firmicutes; c__Bacilli; o__Lactobacillales; f__Lactobacillaceae; g__Lactobacillus | 0.0582 | 1 |
| ASV487 | d__Bacteria; p__Bacteroidota; c__Bacteroidia; o__Bacteroidales; f__Bacteroidaceae; g__Bacteroides | 0.0018 | 1 |
| ASV418 | d__Bacteria; p__Firmicutes; c__Clostridia; o__Lachnospirales; f__Lachnospiraceae | 0.0059 | 1 |
| ASV167 | d__Bacteria; p__Actinobacteriota; c__Actinobacteria; o__Micrococcales; f__Microbacteriaceae | 0.0010 | 1 |
| ASV446 | d__Bacteria; p__Firmicutes; c__Bacilli; o__Erysipelotrichales; f__Erysipelotrichaceae; g__Dielma | 0.0018 | 1 |
| ASV84 | d__Bacteria; p__Actinobacteriota; c__Coriobacteriia; o__Coriobacteriales; f__Eggerthellaceae; g__Enterorhabdus | 0.0180 | 1 |
| ASV325 | d__Bacteria; p__Firmicutes; c__Clostridia; o__Peptostreptococcales-Tissierellales; f__Peptostreptococcales-Tissierellales; g__Parvimonas | 0.0027 | 1 |
| ASV12 | d__Bacteria; p__Firmicutes; c__Bacilli; o__Staphylococcales; f__Staphylococcaceae; g__Staphylococcus | 0.0102 | 1 |
| ASV460 | d__Bacteria; p__Firmicutes; c__Bacilli; o__Erysipelotrichales; f__Erysipelatoclostridiaceae; g__Sharpea | 0.0037 | 1 |
| ASV248 | d__Bacteria; p__Firmicutes; c__Clostridia; o__Oscillospirales; f__Ruminococcaceae; g__Subdoligranulum | 0.0040 | 1 |
| ASV407 | d__Bacteria; p__Firmicutes; c__Bacilli; o__Lactobacillales; f__Lactobacillaceae; g__Lactobacillus | 0.0063 | 1 |
| ASV15 | d__Bacteria; p__Firmicutes; c__Bacilli; o__Staphylococcales; f__Staphylococcaceae; g__Staphylococcus | 0.0055 | 1 |
| ASV93 | d__Bacteria; p__Actinobacteriota; c__Coriobacteriia; o__Coriobacteriales; f__Atopobiaceae; g__Olsenella | 0.0078 | 1 |
| ASV242 | d__Bacteria; p__Firmicutes; c__Bacilli; o__Lactobacillales; f__Lactobacillaceae; g__Lactobacillus | 0.3543 | 1 |
| ASV369 | d__Bacteria; p__Firmicutes; c__Bacilli; o__Lactobacillales; f__Lactobacillaceae; g__Lactobacillus | 0.0018 | 1 |
| ASV106 | d__Bacteria; p__Firmicutes; c__Clostridia; o__Peptostreptococcales-Tissierellales; f__Peptostreptococcaceae; g__Terrisporobacter | 0.0259 | 1 |
| ASV85 | d__Bacteria; p__Actinobacteriota; c__Coriobacteriia; o__Coriobacteriales; f__Eggerthellaceae; g__Enterorhabdus | 0.0193 | 1 |
| ASV59 | d__Bacteria; p__Firmicutes; c__Clostridia; o__Oscillospirales; f__Ruminococcaceae; g__Subdoligranulum | 0.0063 | 1 |
| ASV239 | d__Bacteria; p__Firmicutes; c__Bacilli; o__Staphylococcales; f__Staphylococcaceae; g__Staphylococcus | 0.0016 | 1 |
| ASV365 | d__Bacteria; p__Firmicutes; c__Bacilli; o__Lactobacillales; f__Lactobacillaceae; g__Lactobacillus | 0.0932 | 1 |
| ASV82 | d__Bacteria; p__Firmicutes; c__Bacilli; o__Lactobacillales; f__Lactobacillaceae; g__Lactobacillus | 0.1757 | 1 |
| ASV110 | d__Bacteria; p__Firmicutes; c__Clostridia; o__Christensenellales; f__Christensenellaceae; g__Christensenella | 0.0113 | 1 |
| ASV41 | d__Bacteria; p__Proteobacteria; c__Gammaproteobacteria; o__Enterobacterales; f__Morganellaceae; g__Proteus | 0.0040 | 1 |
| ASV38 | d__Bacteria; p__Firmicutes; c__Bacilli; o__Staphylococcales; f__Staphylococcaceae; g__Staphylococcus | 0.0222 | 1 |
| ASV293 | d__Bacteria; p__Firmicutes; c__Clostridia; o__Clostridiales; f__Clostridiaceae; g__Clostridium_sensu_stricto_1 | 0.0019 | 1 |
| ASV437 | d__Bacteria; p__Firmicutes; c__Bacilli; o__Lactobacillales | 0.0019 | 1 |
| ASV78 | d__Bacteria; p__Firmicutes; c__Clostridia; o__Clostridiales; f__Clostridiaceae; g__Sarcina | 0.0180 | 1 |
| ASV348 | d__Bacteria; p__Proteobacteria; c__Gammaproteobacteria; o__Enterobacterales; f__Morganellaceae; g__Morganella | 0.0004 | 1 |
| ASV7 | d__Bacteria; p__Firmicutes; c__Clostridia; o__Peptostreptococcales-Tissierellales; f__Peptostreptococcaceae; g__Terrisporobacter | 0.2323 | 1 |
| ASV479 | d__Bacteria; p__Bacteroidota; c__Bacteroidia; o__Bacteroidales; f__Rikenellaceae; g__Alistipes | 0.0026 | 1 |
| ASV384 | d__Bacteria; p__Firmicutes; c__Clostridia; o__Lachnospirales; f__Lachnospiraceae | 0.0501 | 1 |
| ASV17 | d__Bacteria; p__Firmicutes; c__Clostridia; o__Lachnospirales; f__Lachnospiraceae | 0.0034 | 1 |
| ASV72 | d__Bacteria; p__Actinobacteriota; c__Coriobacteriia; o__Coriobacteriales; f__Atopobiaceae; g__Olsenella | 0.0346 | 1 |
| ASV381 | d__Bacteria; p__Actinobacteriota; c__Actinobacteria; o__Micrococcales; f__Micrococcaceae; g__Rothia | 0.0091 | 0 |
| ASV154 | d__Bacteria; p__Actinobacteriota; c__Actinobacteria; o__Corynebacteriales; f__Corynebacteriaceae; g__Corynebacterium | 0.0030 | 0 |
| ASV36 | d__Bacteria; p__Firmicutes; c__Clostridia; o__Peptostreptococcales-Tissierellales; f__Anaerovoracaceae; g__Mogibacterium | 0.0933 | 0 |
| ASV317 | d__Bacteria; p__Actinobacteriota; c__Actinobacteria; o__Corynebacteriales; f__Corynebacteriaceae; g__Corynebacterium | 0.0000 | 0 |
| ASV54 | d__Bacteria; p__Actinobacteriota; c__Actinobacteria; o__Actinomycetales; f__Actinomycetaceae; g__Actinomyces | 0.0045 | 0 |
| ASV452 | d__Bacteria; p__Firmicutes; c__Clostridia; o__Lachnospirales; f__Lachnospiraceae; g__Johnsonella | 0.0025 | 0 |
| ASV311 | d__Bacteria; p__Actinobacteriota; c__Actinobacteria; o__Micrococcales; f__Micrococcaceae; g__Kocuria | 0.0000 | 0 |
| ASV23 | d__Bacteria; p__Actinobacteriota; c__Coriobacteriia; o__Coriobacteriales; f__Eggerthellaceae; g__uncultured | 0.0622 | 0 |
| ASV403 | d__Bacteria; p__Firmicutes; c__Clostridia; o__Lachnospirales; f__Lachnospiraceae | 0.0086 | 0 |
| ASV282 | d__Bacteria; p__Actinobacteriota; c__Actinobacteria; o__Corynebacteriales; f__Corynebacteriaceae; g__Corynebacterium | 0.0008 | 0 |
| ASV408 | d__Bacteria; p__Firmicutes; c__Clostridia; o__Lachnospirales; f__Lachnospiraceae; g__Lachnoanaerobaculum | 0.0084 | 0 |
| ASV469 | d__Bacteria; p__Proteobacteria; c__Gammaproteobacteria; o__Pasteurellales; f__Pasteurellaceae; g__Actinobacillus | 0.0055 | 0 |
| ASV499 | d__Bacteria; p__Patescibacteria; c__Gracilibacteria; o__Absconditabacteriales_(SR1); f__Absconditabacteriales_(SR1); g__Absconditabacteriales_(SR1) | 0.0073 | 0 |
| ASV422 | d__Bacteria; p__Firmicutes; c__Bacilli; o__Lactobacillales; f__Streptococcaceae; g__Streptococcus | 0.0023 | 0 |
| ASV146 | d__Bacteria; p__Actinobacteriota; c__Actinobacteria; o__Corynebacteriales; f__Corynebacteriaceae; g__Corynebacterium | 0.0049 | 0 |
| ASV443 | d__Bacteria; p__Actinobacteriota; c__Actinobacteria; o__Actinomycetales; f__Actinomycetaceae; g__Arcanobacterium | 0.0015 | 0 |
| ASV213 | d__Bacteria; p__Firmicutes; c__Clostridia; o__Peptostreptococcales-Tissierellales; f__Anaerovoracaceae; g__Mogibacterium | 0.0014 | 0 |
| ASV379 | d__Bacteria; p__Firmicutes; c__Clostridia; o__Peptostreptococcales-Tissierellales; f__Peptostreptococcaceae; g__Proteocatella | 0.0036 | 0 |
| ASV436 | d__Bacteria; p__Firmicutes; c__Clostridia; o__Lachnospirales; f__Lachnospiraceae; g__Johnsonella | 0.0026 | 0 |
| ASV483 | d__Bacteria; p__Firmicutes; c__Clostridia; o__Lachnospirales; f__Lachnospiraceae; g__[Ruminococcus]_torques_group | 0.0016 | 0 |
| ASV308 | d__Bacteria; p__Firmicutes; c__Bacilli; o__Lactobacillales; f__Leuconostocaceae; g__Leuconostoc | 0.0000 | 0 |
| ASV186 | d__Bacteria; p__Actinobacteriota; c__Actinobacteria; o__Corynebacteriales; f__Corynebacteriaceae; g__Corynebacterium | 0.0022 | 0 |
| ASV447 | d__Bacteria; p__Firmicutes; c__Clostridia; o__Lachnospirales; f__Lachnospiraceae; g__Johnsonella | 0.0025 | 0 |
| ASV212 | d__Bacteria; p__Firmicutes; c__Clostridia; o__Lachnospirales; f__Lachnospiraceae; g__Blautia | 0.0019 | 0 |
| ASV163 | d__Bacteria; p__Firmicutes; c__Clostridia; o__Peptostreptococcales-Tissierellales; f__Peptostreptococcales-Tissierellales; g__Peptoniphilus | 0.0018 | 0 |
| ASV162 | d__Bacteria; p__Firmicutes; c__Clostridia; o__Peptostreptococcales-Tissierellales; f__Anaerovoracaceae; g__Mogibacterium | 0.0033 | 0 |
| ASV170 | d__Bacteria; p__Firmicutes; c__Clostridia; o__Lachnospirales; f__Lachnospiraceae | 0.0041 | 0 |
| ASV270 | d__Bacteria; p__Proteobacteria; c__Gammaproteobacteria; o__Enterobacterales; f__Enterobacteriaceae; g__Klebsiella | 0.0075 | 0 |
| ASV259 | d__Bacteria; p__Actinobacteriota; c__Actinobacteria; o__Micrococcales; f__Microbacteriaceae; g__Pseudoclavibacter | 0.0003 | 0 |
| ASV432 | d__Bacteria; p__Firmicutes; c__Bacilli; o__Erysipelotrichales; f__Erysipelotrichaceae; g__uncultured | 0.0021 | 0 |
| ASV199 | d__Bacteria; p__Firmicutes; c__Clostridia; o__Oscillospirales; f__[Eubacterium]_coprostanoligenes_group; g__[Eubacterium]_coprostanoligenes_group | 0.0027 | 0 |
| ASV440 | d__Bacteria; p__Actinobacteriota; c__Actinobacteria; o__Actinomycetales; f__Actinomycetaceae; g__Arcanobacterium | 0.0026 | 0 |
| ASV172 | d__Bacteria; p__Firmicutes; c__Clostridia; o__Lachnospirales; f__Lachnospiraceae; g__Epulopiscium | 0.0034 | 0 |
| ASV161 | d__Bacteria; p__Firmicutes; c__Clostridia; o__Peptostreptococcales-Tissierellales; f__Peptostreptococcales-Tissierellales; g__Anaerococcus | 0.0027 | 0 |
| ASV489 | d__Bacteria; p__Firmicutes; c__Bacilli; o__Staphylococcales; f__Staphylococcaceae; g__Staphylococcus | 0.0034 | 0 |
| ASV420 | d__Bacteria; p__Proteobacteria; c__Gammaproteobacteria; o__Pasteurellales; f__Pasteurellaceae; g__Actinobacillus | 0.0014 | 0 |
| ASV198 | d__Bacteria; p__Firmicutes; c__Clostridia; o__Christensenellales; f__Christensenellaceae; g__Christensenellaceae_R-7_group | 0.0023 | 0 |
| ASV164 | d__Bacteria; p__Firmicutes; c__Bacilli; o__Lactobacillales; f__Streptococcaceae; g__Streptococcus | 0.3008 | 0 |
| ASV133 | d__Bacteria; p__Firmicutes; c__Clostridia; o__Oscillospirales; f__[Eubacterium]_coprostanoligenes_group; g__[Eubacterium]_coprostanoligenes_group | 0.0051 | 0 |
| ASV309 | d__Bacteria; p__Proteobacteria; c__Gammaproteobacteria; o__Enterobacterales; f__Enterobacteriaceae | 0.0000 | 0 |
| ASV92 | d__Bacteria; p__Firmicutes; c__Clostridia; o__Oscillospirales; f__Oscillospiraceae; g__NK4A214_group | 0.0185 | 0 |
| ASV246 | d__Bacteria; p__Proteobacteria; c__Gammaproteobacteria; o__Pasteurellales; f__Pasteurellaceae; g__Actinobacillus | 0.0156 | 0 |
| ASV327 | d__Bacteria; p__Firmicutes; c__Bacilli; o__Lactobacillales; f__Aerococcaceae; g__Facklamia | 0.0000 | 0 |
| ASV314 | d__Bacteria; p__Actinobacteriota; c__Actinobacteria; o__Micrococcales; f__Brevibacteriaceae; g__Brevibacterium | 0.0000 | 0 |
| ASV500 | d__Bacteria; p__Actinobacteriota; c__Actinobacteria; o__Propionibacteriales; f__Propionibacteriaceae; g__uncultured | 0.0027 | 0 |
| ASV415 | d__Bacteria; p__Fusobacteriota; c__Fusobacteriia; o__Fusobacteriales; f__Fusobacteriaceae; g__Fusobacterium | 0.0115 | 0 |
| ASV387 | d__Bacteria; p__Firmicutes; c__Bacilli; o__Lactobacillales; f__Streptococcaceae; g__Streptococcus | 0.1289 | 0 |
| ASV496 | d__Bacteria; p__Actinobacteriota; c__Actinobacteria; o__Propionibacteriales; f__Propionibacteriaceae; g__Tessaracoccus | 0.0026 | 0 |
| ASV450 | d__Bacteria; p__Firmicutes; c__Clostridia; o__Peptostreptococcales-Tissierellales; f__Peptostreptococcaceae; g__Proteocatella | 0.0054 | 0 |
| ASV334 | d__Bacteria; p__Actinobacteriota; c__Actinobacteria; o__Corynebacteriales; f__Corynebacteriaceae; g__Corynebacterium | 0.0000 | 0 |
| ASV362 | d__Bacteria; p__Proteobacteria; c__Gammaproteobacteria; o__Pasteurellales; f__Pasteurellaceae; g__Actinobacillus | 0.0016 | 0 |
| ASV96 | d__Bacteria; p__Actinobacteriota; c__Actinobacteria; o__Micrococcales; f__Micrococcaceae; g__Rothia | 1.5089 | 0 |
| ASV346 | d__Bacteria; p__Firmicutes; c__Bacilli; o__Lactobacillales; f__Streptococcaceae; g__Streptococcus | 0.0552 | 0 |
| ASV303 | d__Bacteria; p__Firmicutes; c__Clostridia; o__Peptostreptococcales-Tissierellales; f__Peptostreptococcales-Tissierellales; g__Parvimonas | 0.0052 | 0 |
| ASV26 | d__Bacteria; p__Actinobacteriota; c__Actinobacteria; o__Corynebacteriales; f__Corynebacteriaceae; g__Corynebacterium | 0.0152 | 0 |
| ASV209 | d__Bacteria; p__Proteobacteria; c__Gammaproteobacteria; o__Pasteurellales; f__Pasteurellaceae; g__Pasteurella | 0.7231 | 0 |
| ASV98 | d__Bacteria; p__Actinobacteriota; c__Actinobacteria; o__Actinomycetales; f__Actinomycetaceae; g__Actinomyces | 0.0762 | 0 |
| ASV33 | d__Bacteria; p__Firmicutes; c__Clostridia; o__Peptostreptococcales-Tissierellales; f__Anaerovoracaceae; g__Family_XIII_AD3011_group | 0.0257 | 0 |
| ASV115 | d__Bacteria; p__Firmicutes; c__Clostridia; o__Peptococcales; f__Peptococcaceae; g__uncultured | 0.0062 | 0 |
| ASV394 | d__Bacteria; p__Actinobacteriota; c__Actinobacteria; o__Actinomycetales; f__Actinomycetaceae; g__Actinomyces | 0.0344 | 0 |
| ASV194 | d__Bacteria; p__Actinobacteriota; c__Actinobacteria; o__Corynebacteriales; f__Corynebacteriaceae; g__Corynebacterium | 0.0021 | 0 |
| ASV355 | d__Bacteria; p__Proteobacteria; c__Alphaproteobacteria; o__Caulobacterales; f__Caulobacteraceae; g__uncultured | 0.0314 | 0 |
| ASV130 | d__Bacteria; p__Firmicutes; c__Clostridia; o__Oscillospirales; f__Oscillospiraceae; g__UCG-005 | 0.0033 | 0 |
| ASV140 | d__Bacteria; p__Firmicutes; c__Bacilli; o__Lactobacillales; f__Streptococcaceae; g__Streptococcus | 0.0027 | 0 |
| ASV22 | d__Bacteria; p__Actinobacteriota; c__Actinobacteria; o__Corynebacteriales; f__Corynebacteriaceae; g__Corynebacterium | 0.0941 | 0 |
| ASV227 | d__Bacteria; p__Proteobacteria; c__Gammaproteobacteria; o__Pasteurellales; f__Pasteurellaceae; g__Actinobacillus | 0.0169 | 0 |
| ASV184 | d__Bacteria; p__Actinobacteriota; c__Actinobacteria; o__Micrococcales; f__Micrococcaceae; g__Kocuria | 0.0015 | 0 |
| ASV413 | d__Bacteria; p__Firmicutes; c__Bacilli; o__Lactobacillales; f__Streptococcaceae; g__Streptococcus | 0.0059 | 0 |
| ASV312 | d__Bacteria; p__Actinobacteriota; c__Actinobacteria; o__Micrococcales; f__Microbacteriaceae | 0.0000 | 0 |
| ASV188 | d__Bacteria; p__Firmicutes; c__Negativicutes; o__Veillonellales-Selenomonadales; f__Selenomonadaceae; g__Anaerovibrio | 0.0180 | 0 |
| ASV50 | d__Bacteria; p__Firmicutes; c__Bacilli; o__Lactobacillales; f__Streptococcaceae; g__Streptococcus | 0.0084 | 0 |
| ASV196 | d__Bacteria; p__Firmicutes; c__Clostridia; o__Christensenellales; f__Christensenellaceae; g__Christensenellaceae_R-7_group | 0.0027 | 0 |
| ASV89 | d__Bacteria; p__Firmicutes; c__Clostridia; o__Oscillospirales; f__Oscillospiraceae; g__UCG-002 | 0.0093 | 0 |
| ASV205 | d__Bacteria; p__Firmicutes; c__Clostridia; o__Oscillospirales; f__Oscillospiraceae | 0.0088 | 0 |
| ASV238 | d__Bacteria; p__Firmicutes; c__Bacilli; o__Staphylococcales; f__Gemellaceae; g__Gemella | 0.0150 | 0 |
| ASV230 | d__Bacteria; p__Firmicutes; c__Bacilli; o__Lactobacillales; f__Streptococcaceae; g__Streptococcus | 0.0679 | 0 |
| ASV416 | d__Bacteria; p__Actinobacteriota; c__Actinobacteria; o__Corynebacteriales; f__Corynebacteriaceae; g__Corynebacterium | 0.0425 | 0 |
| ASV29 | d__Bacteria; p__Actinobacteriota; c__Actinobacteria; o__Actinomycetales; f__Actinomycetaceae; g__Trueperella | 0.0387 | 0 |
| ASV264 | d__Bacteria; p__Proteobacteria; c__Gammaproteobacteria; o__Pasteurellales; f__Pasteurellaceae; g__Actinobacillus | 0.0242 | 0 |
| ASV39 | d__Bacteria; p__Actinobacteriota; c__Actinobacteria; o__Actinomycetales; f__Actinomycetaceae; g__Trueperella | 0.0093 | 0 |
| ASV326 | d__Bacteria; p__Actinobacteriota; c__Actinobacteria; o__Corynebacteriales; f__Corynebacteriaceae; g__Corynebacterium | 0.0000 | 0 |
| ASV435 | d__Bacteria; p__Patescibacteria; c__Saccharimonadia; o__Saccharimonadales; f__Saccharimonadaceae; g__Candidatus_Saccharimonas | 0.0016 | 0 |
| ASV357 | d__Bacteria; p__Proteobacteria; c__Gammaproteobacteria; o__Burkholderiales; f__Burkholderiaceae; g__Burkholderia-Caballeronia-Paraburkholderia | 0.0185 | 0 |
| ASV430 | d__Bacteria; p__Actinobacteriota; c__Actinobacteria; o__Corynebacteriales; f__Corynebacteriaceae; g__Corynebacterium | 0.0025 | 0 |
| ASV491 | d__Bacteria; p__Proteobacteria; c__Gammaproteobacteria; o__Pseudomonadales; f__Moraxellaceae; g__Acinetobacter | 0.0038 | 0 |
| ASV322 | d__Bacteria; p__Actinobacteriota; c__Actinobacteria; o__Micrococcales; f__Dermabacteraceae; g__Brachybacterium | 0.0000 | 0 |
| ASV267 | d__Bacteria; p__Proteobacteria; c__Gammaproteobacteria; o__Pasteurellales; f__Pasteurellaceae; g__Actinobacillus | 0.0869 | 0 |
| ASV297 | d__Bacteria; p__Synergistota; c__Synergistia; o__Synergistales; f__Synergistaceae; g__Cloacibacillus | 0.0014 | 0 |
| ASV425 | d__Bacteria; p__Proteobacteria; c__Gammaproteobacteria; o__Pasteurellales; f__Pasteurellaceae; g__Actinobacillus | 0.0016 | 0 |
| ASV44 | d__Bacteria; p__Firmicutes; c__Clostridia; o__Lachnospirales; f__Lachnospiraceae; g__[Eubacterium]_fissicatena_group | 0.0152 | 0 |
| ASV25 | d__Bacteria; p__Proteobacteria; c__Gammaproteobacteria; o__Pasteurellales; f__Pasteurellaceae; g__Actinobacillus | 0.2130 | 0 |
| ASV120 | d__Bacteria; p__Actinobacteriota; c__Actinobacteria; o__Propionibacteriales; f__Propionibacteriaceae; g__Cutibacterium | 0.0052 | 0 |
| ASV18 | d__Bacteria; p__Proteobacteria; c__Gammaproteobacteria; o__Pseudomonadales; f__Moraxellaceae; g__Moraxella | 0.3950 | 0 |
| ASV75 | d__Bacteria; p__Firmicutes; c__Clostridia; o__Clostridiales; f__Clostridiaceae; g__Clostridium_sensu_stricto_6 | 0.0253 | 0 |
| ASV344 | d__Bacteria; p__Synergistota; c__Synergistia; o__Synergistales; f__Synergistaceae; g__Cloacibacillus | 0.0015 | 0 |
| ASV202 | d__Bacteria; p__Firmicutes; c__Clostridia; o__Peptostreptococcales-Tissierellales; f__Anaerovoracaceae; g__Family_XIII_AD3011_group | 0.0014 | 0 |
| ASV339 | d__Bacteria; p__Firmicutes; c__Bacilli; o__Lactobacillales; f__Aerococcaceae; g__Aerococcus | 0.0010 | 0 |
| ASV79 | d__Bacteria; p__Firmicutes; c__Clostridia; o__Peptostreptococcales-Tissierellales; f__Anaerovoracaceae; g__Family_XIII_AD3011_group | 0.0222 | 0 |
| ASV399 | d__Bacteria; p__Actinobacteriota; c__Actinobacteria | 0.0143 | 0 |
| ASV123 | d__Bacteria; p__Firmicutes; c__Clostridia; o__Lachnospirales; f__Lachnospiraceae; g__[Eubacterium]_hallii_group | 0.0080 | 0 |
| ASV269 | d__Bacteria; p__Proteobacteria; c__Gammaproteobacteria; o__Enterobacterales; f__Enterobacteriaceae | 0.0015 | 0 |
| ASV9 | d__Bacteria; p__Proteobacteria; c__Gammaproteobacteria; o__Pasteurellales; f__Pasteurellaceae; g__Actinobacillus | 0.0490 | 0 |
| ASV402 | d__Bacteria; p__Patescibacteria; c__Saccharimonadia; o__Saccharimonadales; f__Saccharimonadales; g__Saccharimonadales | 0.0427 | 0 |
| ASV352 | d__Bacteria; p__Actinobacteriota; c__Actinobacteria; o__Actinomycetales; f__Actinomycetaceae; g__Actinomyces | 0.0030 | 0 |
| ASV473 | d__Bacteria; p__Firmicutes; c__Clostridia; o__Oscillospirales; f__Oscillospirales; g__Hydrogenoanaerobacterium | 0.0018 | 0 |
| ASV405 | d__Bacteria; p__Actinobacteriota; c__Actinobacteria; o__Actinomycetales; f__Actinomycetaceae; g__Actinomyces | 0.0070 | 0 |
| ASV373 | d__Bacteria; p__Firmicutes; c__Bacilli; o__Lactobacillales; f__Streptococcaceae; g__Streptococcus | 0.0021 | 0 |
| ASV254 | d__Bacteria; p__Proteobacteria; c__Gammaproteobacteria; o__Enterobacterales; f__Enterobacteriaceae | 0.0023 | 0 |
| ASV135 | d__Bacteria; p__Firmicutes; c__Negativicutes; o__Acidaminococcales; f__Acidaminococcaceae; g__Phascolarctobacterium | 0.0019 | 0 |
| ASV197 | d__Bacteria; p__Actinobacteriota; c__Actinobacteria; o__Actinomycetales; f__Actinomycetaceae; g__Actinomyces | 0.0014 | 0 |
| ASV335 | d__Bacteria; p__Cyanobacteria; c__Cyanobacteriia; o__Chloroplast; f__Chloroplast; g__Chloroplast | 0.0049 | 0 |
| ASV31 | d__Bacteria; p__Proteobacteria; c__Gammaproteobacteria; o__Pasteurellales; f__Pasteurellaceae; g__Actinobacillus | 0.0633 | 0 |
| ASV459 | d__Bacteria; p__Proteobacteria; c__Gammaproteobacteria; o__Pseudomonadales; f__Moraxellaceae | 0.0078 | 0 |
| ASV77 | d__Bacteria; p__Firmicutes; c__Negativicutes; o__Acidaminococcales; f__Acidaminococcaceae; g__Phascolarctobacterium | 0.0505 | 0 |
| ASV427 | d__Bacteria; p__Firmicutes; c__Clostridia; o__Lachnospirales; f__Lachnospiraceae; g__Oribacterium | 0.0021 | 0 |
| ASV439 | d__Bacteria; p__Firmicutes; c__Clostridia; o__Oscillospirales; f__Ruminococcaceae; g__Incertae_Sedis | 0.0015 | 0 |
| ASV438 | d__Bacteria; p__Firmicutes; c__Clostridia; o__Peptostreptococcales-Tissierellales; f__Peptostreptococcales-Tissierellales; g__Murdochiella | 0.0018 | 0 |
| ASV465 | d__Bacteria; p__Firmicutes; c__Clostridia; o__Clostridia; f__Hungateiclostridiaceae; g__Mageibacillus | 0.0014 | 0 |
| ASV263 | d__Bacteria; p__Proteobacteria; c__Gammaproteobacteria; o__Enterobacterales; f__Enterobacteriaceae; g__Klebsiella | 0.0416 | 0 |
| ASV252 | d__Bacteria; p__Firmicutes; c__Clostridia; o__Oscillospirales; f__Oscillospiraceae | 0.0189 | 0 |
| ASV330 | d__Bacteria; p__Proteobacteria; c__Gammaproteobacteria; o__Pseudomonadales; f__Moraxellaceae; g__Acinetobacter | 0.0000 | 0 |
| ASV137 | d__Bacteria; p__Verrucomicrobiota; c__Verrucomicrobiae; o__Verrucomicrobiales; f__Akkermansiaceae; g__Akkermansia | 0.0052 | 0 |
| ASV390 | d__Bacteria; p__Firmicutes; c__Clostridia; o__Peptostreptococcales-Tissierellales; f__Anaerovoracaceae; g__[Eubacterium]_brachy_group | 0.0627 | 0 |
| ASV8 | d__Bacteria; p__Proteobacteria; c__Gammaproteobacteria; o__Enterobacterales; f__Enterobacteriaceae; g__Escherichia-Shigella | 7.2643 | 0 |
| ASV503 | d__Bacteria; p__Proteobacteria; c__Gammaproteobacteria; o__Pseudomonadales; f__Pseudomonadaceae; g__Pseudomonas | 0.0014 | 0 |
| ASV453 | d__Bacteria; p__Actinobacteriota; c__Actinobacteria; o__Corynebacteriales; f__Corynebacteriaceae; g__Corynebacterium | 0.0165 | 0 |
| ASV220 | d__Bacteria; p__Firmicutes; c__Bacilli; o__Lactobacillales; f__Aerococcaceae; g__Globicatella | 0.1453 | 0 |
| ASV468 | d__Bacteria; p__Fusobacteriota; c__Fusobacteriia; o__Fusobacteriales; f__Fusobacteriaceae; g__Fusobacterium | 0.0059 | 0 |
| ASV111 | d__Bacteria; p__Firmicutes; c__Clostridia; o__Christensenellales; f__Christensenellaceae; g__Christensenellaceae_R-7_group | 0.0060 | 0 |
| ASV240 | d__Bacteria; p__Firmicutes; c__Bacilli; o__Lactobacillales; f__Streptococcaceae; g__Streptococcus | 1.3221 | 0 |
| ASV451 | d__Bacteria; p__Firmicutes; c__Clostridia; o__Peptostreptococcales-Tissierellales; f__Anaerovoracaceae; g__Family_XIII_AD3011_group | 0.0049 | 0 |
| ASV336 | d__Bacteria; p__Firmicutes; c__Clostridia; o__Monoglobales; f__Monoglobaceae; g__Monoglobus | 0.0004 | 0 |
| ASV370 | d__Bacteria; p__Firmicutes; c__Bacilli; o__Lactobacillales; f__Streptococcaceae; g__Streptococcus | 0.0914 | 0 |
| ASV176 | d__Bacteria; p__Actinobacteriota; c__Actinobacteria; o__Corynebacteriales; f__Corynebacteriaceae; g__Corynebacterium | 0.0015 | 0 |
| ASV404 | d__Bacteria; p__Firmicutes; c__Clostridia; o__Peptostreptococcales-Tissierellales; f__Peptostreptococcaceae; g__Filifactor | 0.0074 | 0 |
| ASV177 | d__Bacteria; p__Actinobacteriota; c__Actinobacteria; o__Actinomycetales; f__Actinomycetaceae; g__Actinomyces | 0.0029 | 0 |
| ASV157 | d__Bacteria; p__Firmicutes; c__Bacilli; o__RF39; f__RF39; g__RF39 | 0.0019 | 0 |
| ASV289 | d__Bacteria; p__Firmicutes; c__Clostridia; o__Oscillospirales; f__Ruminococcaceae; g__uncultured | 0.0025 | 0 |
| ASV52 | d__Bacteria; p__Firmicutes; c__Clostridia; o__Clostridiales; f__Clostridiaceae; g__Clostridium_sensu_stricto_6 | 0.0052 | 0 |
| ASV10 | d__Bacteria; p__Firmicutes; c__Bacilli; o__Staphylococcales; f__Staphylococcaceae; g__Staphylococcus | 0.1287 | 0 |
| ASV472 | d__Bacteria; p__Actinobacteriota; c__Actinobacteria; o__Actinomycetales; f__Actinomycetaceae; g__Actinomyces | 0.0014 | 0 |
| ASV104 | d__Bacteria; p__Firmicutes; c__Clostridia; o__Lachnospirales; f__Lachnospiraceae; g__[Eubacterium]_hallii_group | 0.0119 | 0 |
| ASV284 | d__Bacteria; p__Proteobacteria; c__Gammaproteobacteria; o__Pasteurellales; f__Pasteurellaceae; g__Actinobacillus | 0.1589 | 0 |
| ASV257 | d__Bacteria; p__Proteobacteria; c__Gammaproteobacteria; o__Enterobacterales; f__Enterobacteriaceae; g__Klebsiella | 0.0062 | 0 |
| ASV376 | d__Bacteria; p__Fusobacteriota; c__Fusobacteriia; o__Fusobacteriales; f__Leptotrichiaceae; g__Leptotrichia | 0.0016 | 0 |
| ASV101 | d__Bacteria; p__Firmicutes; c__Clostridia; o__Lachnospirales; f__Lachnospiraceae; g__Blautia | 0.0070 | 0 |
| ASV169 | d__Bacteria; p__Firmicutes; c__Clostridia; o__Oscillospirales; f__[Eubacterium]_coprostanoligenes_group; g__[Eubacterium]_coprostanoligenes_group | 0.0038 | 0 |
| ASV428 | d__Bacteria; p__Patescibacteria; c__Saccharimonadia; o__Saccharimonadales; f__Saccharimonadaceae; g__Candidatus_Saccharimonas | 0.0026 | 0 |
| ASV320 | d__Bacteria; p__Proteobacteria; c__Gammaproteobacteria; o__Enterobacterales; f__Enterobacteriaceae | 0.0000 | 0 |
| ASV74 | d__Bacteria; p__Firmicutes; c__Clostridia; o__Oscillospirales; f__Ruminococcaceae; g__Ruminococcus | 0.0829 | 0 |
| ASV47 | d__Bacteria; p__Firmicutes; c__Clostridia; o__Peptostreptococcales-Tissierellales; f__Peptostreptococcales-Tissierellales; g__Helcococcus | 0.0015 | 0 |
| ASV442 | d__Bacteria; p__Firmicutes; c__Clostridia; o__Peptostreptococcales-Tissierellales; f__Anaerovoracaceae; g__[Eubacterium]_nodatum_group | 0.0022 | 0 |
| ASV91 | d__Bacteria; p__Firmicutes; c__Clostridia; o__Lachnospirales; f__Lachnospiraceae; g__[Ruminococcus]_gauvreauii_group | 0.0128 | 0 |
| ASV235 | d__Bacteria; p__Firmicutes; c__Negativicutes; o__Veillonellales-Selenomonadales; f__Veillonellaceae; g__Megasphaera | 0.0025 | 0 |
| ASV493 | d__Bacteria; p__Proteobacteria; c__Gammaproteobacteria; o__Burkholderiales; f__Burkholderiaceae; g__Burkholderia-Caballeronia-Paraburkholderia | 0.0030 | 0 |
| ASV132 | d__Bacteria; p__Firmicutes; c__Bacilli; o__Lactobacillales; f__Enterococcaceae; g__Enterococcus | 0.1890 | 0 |
| ASV37 | d__Bacteria; p__Proteobacteria; c__Gammaproteobacteria; o__Pasteurellales; f__Pasteurellaceae; g__Actinobacillus | 0.0870 | 0 |
| ASV217 | d__Bacteria; p__Actinobacteriota; c__Actinobacteria; o__Corynebacteriales; f__Corynebacteriaceae; g__Corynebacterium | 0.0015 | 0 |
| ASV444 | d__Bacteria; p__Firmicutes; c__Bacilli; o__Lactobacillales | 0.0018 | 0 |
| ASV42 | d__Bacteria; p__Actinobacteriota; c__Actinobacteria; o__Corynebacteriales; f__Corynebacteriaceae; g__Corynebacterium | 0.0110 | 0 |
| ASV272 | d__Bacteria; p__Proteobacteria; c__Gammaproteobacteria; o__Enterobacterales; f__Enterobacteriaceae | 0.0014 | 0 |
| ASV19 | d__Bacteria; p__Actinobacteriota; c__Actinobacteria; o__Corynebacteriales; f__Corynebacteriaceae; g__Corynebacterium | 0.0392 | 0 |
| ASV265 | d__Bacteria; p__Proteobacteria; c__Gammaproteobacteria; o__Enterobacterales; f__Enterobacteriaceae; g__Klebsiella | 0.0303 | 0 |
| ASV358 | d__Bacteria; p__Cyanobacteria; c__Vampirivibrionia; o__Obscuribacterales; f__Obscuribacteraceae; g__Obscuribacteraceae | 0.0085 | 0 |
| ASV233 | d__Bacteria; p__Actinobacteriota; c__Actinobacteria; o__Corynebacteriales; f__Corynebacteriaceae; g__Corynebacterium | 0.0019 | 0 |
| ASV249 | d__Bacteria; p__Firmicutes; c__Clostridia; o__Peptostreptococcales-Tissierellales; f__Anaerovoracaceae; g__Mogibacterium | 0.0018 | 0 |
| ASV236 | d__Bacteria; p__Actinobacteriota; c__Actinobacteria; o__Micrococcales; f__Brevibacteriaceae; g__Brevibacterium | 0.0056 | 0 |
| ASV305 | d__Bacteria; p__Proteobacteria; c__Gammaproteobacteria; o__Enterobacterales; f__Enterobacteriaceae | 0.0000 | 0 |
| ASV423 | d__Bacteria; p__Firmicutes; c__Clostridia; o__Peptostreptococcales-Tissierellales; f__Anaerovoracaceae | 0.0019 | 0 |
| ASV179 | d__Bacteria; p__Firmicutes; c__Bacilli; o__Lactobacillales; f__Streptococcaceae; g__Streptococcus | 0.0859 | 0 |
| ASV474 | d__Bacteria; p__Firmicutes; c__Clostridia; o__Oscillospirales; f__Oscillospiraceae; g__UCG-002 | 0.0019 | 0 |
| ASV183 | d__Bacteria; p__Actinobacteriota; c__Actinobacteria; o__Corynebacteriales; f__Corynebacteriaceae; g__Corynebacterium | 0.0449 | 0 |
| ASV410 | d__Bacteria; p__Actinobacteriota; c__Actinobacteria; o__Corynebacteriales; f__Corynebacteriaceae; g__Corynebacterium | 0.0038 | 0 |
| ASV166 | d__Bacteria; p__Firmicutes; c__Clostridia; o__Lachnospirales; f__Lachnospiraceae; g__Blautia | 0.0014 | 0 |
| ASV131 | d__Bacteria; p__Firmicutes; c__Clostridia; o__Christensenellales; f__Christensenellaceae; g__Christensenellaceae_R-7_group | 0.0040 | 0 |
| ASV203 | d__Bacteria; p__Actinobacteriota; c__Actinobacteria; o__Micrococcales; f__Micrococcaceae; g__Rothia | 0.0021 | 0 |
| ASV319 | d__Bacteria; p__Fusobacteriota; c__Fusobacteriia; o__Fusobacteriales; f__Fusobacteriaceae; g__Fusobacterium | 0.0082 | 0 |
| ASV190 | d__Bacteria; p__Firmicutes; c__Bacilli; o__Lactobacillales; f__Leuconostocaceae; g__Weissella | 0.0033 | 0 |
| ASV32 | d__Bacteria; p__Actinobacteriota; c__Actinobacteria; o__Corynebacteriales; f__Corynebacteriaceae; g__Corynebacterium | 0.0134 | 0 |
| ASV388 | d__Bacteria; p__Actinobacteriota; c__Actinobacteria; o__Actinomycetales; f__Actinomycetaceae; g__Arcanobacterium | 0.1375 | 0 |
| ASV298 | d__Bacteria; p__Actinobacteriota; c__Actinobacteria; o__Corynebacteriales; f__Corynebacteriaceae; g__Corynebacterium | 0.0067 | 0 |
| ASV321 | d__Bacteria; p__Firmicutes; c__Bacilli; o__Staphylococcales; f__Staphylococcaceae; g__Nosocomiicoccus | 0.0000 | 0 |
| ASV225 | d__Bacteria; p__Actinobacteriota; c__Actinobacteria; o__Actinomycetales; f__Actinomycetaceae; g__Actinomyces | 0.0092 | 0 |
| ASV30 | d__Bacteria; p__Actinobacteriota; c__Actinobacteria; o__Corynebacteriales; f__Corynebacteriaceae; g__Corynebacterium | 0.0033 | 0 |
| ASV302 | d__Bacteria; p__Firmicutes; c__Bacilli; o__Lactobacillales; f__Enterococcaceae; g__Enterococcus | 0.0000 | 0 |
| ASV256 | d__Bacteria; p__Firmicutes; c__Bacilli; o__Staphylococcales; f__Staphylococcaceae; g__Staphylococcus | 0.0047 | 0 |
| ASV144 | d__Bacteria; p__Firmicutes; c__Clostridia; o__Lachnospirales; f__Lachnospiraceae; g__Lachnospiraceae_XPB1014_group | 0.0170 | 0 |
| ASV105 | d__Bacteria; p__Firmicutes; c__Bacilli; o__Lactobacillales; f__Streptococcaceae; g__Streptococcus | 0.1109 | 0 |
| ASV174 | d__Bacteria; p__Actinobacteriota; c__Actinobacteria; o__Corynebacteriales; f__Corynebacteriaceae; g__Corynebacterium | 0.0055 | 0 |
| ASV102 | d__Bacteria; p__Firmicutes; c__Clostridia; o__Lachnospirales; f__Lachnospiraceae; g__CHKCI001 | 0.0092 | 0 |
| ASV5 | d__Bacteria; p__Proteobacteria; c__Gammaproteobacteria; o__Pseudomonadales; f__Moraxellaceae; g__Moraxella | 0.3069 | 0 |
| ASV207 | d__Bacteria; p__Firmicutes; c__Clostridia; o__Oscillospirales; f__Oscillospiraceae; g__uncultured | 0.0047 | 0 |
| ASV391 | d__Bacteria; p__Firmicutes; c__Clostridia; o__Lachnospirales; f__Lachnospiraceae | 0.0532 | 0 |
| ASV343 | d__Bacteria; p__Firmicutes; c__Bacilli; o__Bacillales; f__Bacillaceae; g__Bacillus | 0.0025 | 0 |
| ASV331 | d__Bacteria; p__Firmicutes; c__Bacilli; o__Staphylococcales; f__Staphylococcaceae; g__Corticicoccus | 0.0000 | 0 |
| ASV232 | d__Bacteria; p__Firmicutes; c__Negativicutes; o__Veillonellales-Selenomonadales; f__Veillonellaceae; g__Veillonella | 0.5712 | 0 |
| ASV324 | d__Bacteria; p__Actinobacteriota; c__Actinobacteria; o__Micrococcales; f__Micrococcaceae; g__Nesterenkonia | 0.0000 | 0 |
| ASV280 | d__Bacteria; p__Actinobacteriota; c__Actinobacteria; o__Corynebacteriales; f__Corynebacteriaceae; g__Corynebacterium | 0.0010 | 0 |
| ASV398 | d__Bacteria; p__Firmicutes; c__Bacilli; o__Erysipelotrichales; f__Erysipelotrichaceae; g__uncultured | 0.0273 | 0 |
| ASV222 | d__Bacteria; p__Proteobacteria; c__Gammaproteobacteria; o__Pasteurellales; f__Pasteurellaceae; g__Actinobacillus | 0.1057 | 0 |
| ASV347 | d__Bacteria; p__Firmicutes; c__Bacilli; o__Lactobacillales; f__Streptococcaceae; g__Streptococcus | 0.0048 | 0 |
| ASV16 | d__Bacteria; p__Actinobacteriota; c__Actinobacteria; o__Micrococcales; f__Micrococcaceae; g__Rothia | 0.0908 | 0 |
| ASV456 | d__Bacteria; p__Patescibacteria; c__Saccharimonadia; o__Saccharimonadales; f__Saccharimonadales; g__Saccharimonadales | 0.0052 | 0 |
| ASV342 | d__Bacteria; p__Firmicutes; c__Bacilli; o__Bacillales; f__Bacillaceae; g__Bacillus | 0.0070 | 0 |
| ASV494 | d__Bacteria; p__Bacteroidota; c__Bacteroidia; o__Flavobacteriales; f__Weeksellaceae; g__Cloacibacterium | 0.0018 | 0 |
| ASV142 | d__Bacteria; p__Firmicutes; c__Clostridia; o__Peptostreptococcales-Tissierellales; f__Anaerovoracaceae; g__Mogibacterium | 0.0023 | 0 |
| ASV485 | d__Bacteria; p__Bacteroidota; c__Bacteroidia; o__Bacteroidales | 0.0016 | 0 |
| ASV221 | d__Bacteria; p__Firmicutes; c__Negativicutes; o__Veillonellales-Selenomonadales; f__Veillonellaceae; g__Megasphaera | 0.0056 | 0 |
| ASV337 | d__Bacteria; p__Firmicutes; c__Bacilli; o__Lactobacillales; f__Streptococcaceae; g__Streptococcus | 0.0008 | 0 |
| ASV386 | d__Bacteria; p__Fusobacteriota; c__Fusobacteriia; o__Fusobacteriales; f__Fusobacteriaceae; g__Fusobacterium | 0.1416 | 0 |
| ASV182 | d__Bacteria; p__Firmicutes; c__Clostridia; o__Lachnospirales; f__Lachnospiraceae; g__Acetitomaculum | 0.0015 | 0 |
| ASV216 | d__Bacteria; p__Firmicutes; c__Clostridia; o__Peptococcales; f__Peptococcaceae; g__Peptococcus | 0.0104 | 0 |
| ASV341 | d__Bacteria; p__Actinobacteriota; c__Actinobacteria; o__Corynebacteriales; f__Corynebacteriaceae; g__Corynebacterium | 0.0237 | 0 |
| ASV35 | d__Bacteria; p__Firmicutes; c__Clostridia; o__Peptostreptococcales-Tissierellales; f__Peptostreptococcales-Tissierellales; g__Helcococcus | 0.0008 | 0 |
| ASV400 | d__Bacteria; p__Proteobacteria; c__Gammaproteobacteria; o__Pasteurellales; f__Pasteurellaceae | 0.0049 | 0 |
| ASV187 | d__Bacteria; p__Firmicutes; c__Bacilli; o__Lactobacillales; f__Streptococcaceae; g__Streptococcus | 0.3954 | 0 |
| ASV276 | d__Bacteria; p__Firmicutes; c__Bacilli; o__Lactobacillales; f__Streptococcaceae; g__Streptococcus | 0.0119 | 0 |
| ASV231 | d__Bacteria; p__Firmicutes; c__Clostridia; o__Oscillospirales; f__Oscillospiraceae; g__uncultured | 0.0025 | 0 |
| ASV414 | d__Bacteria; p__Firmicutes; c__Clostridia; o__Peptostreptococcales-Tissierellales; f__Anaerovoracaceae; g__S5-A14a | 0.0043 | 0 |
| ASV363 | d__Bacteria; p__Proteobacteria; c__Gammaproteobacteria; o__Burkholderiales; f__Burkholderiaceae; g__Ralstonia | 0.0137 | 0 |
| ASV149 | d__Bacteria; p__Proteobacteria; c__Gammaproteobacteria; o__Burkholderiales; f__Burkholderiaceae; g__Lautropia | 0.0014 | 0 |
| ASV457 | d__Bacteria; p__Verrucomicrobiota; c__Chlamydiae; o__Chlamydiales; f__Chlamydiaceae; g__Chlamydia | 0.0328 | 0 |
| ASV288 | d__Bacteria; p__Synergistota; c__Synergistia; o__Synergistales; f__Synergistaceae; g__Cloacibacillus | 0.0019 | 0 |
| ASV393 | d__Bacteria; p__Actinobacteriota; c__Coriobacteriia; o__Coriobacteriales; f__Atopobiaceae; g__Atopobium | 0.0258 | 0 |
| ASV306 | d__Bacteria; p__Proteobacteria; c__Alphaproteobacteria; o__Rhizobiales; f__Rhizobiaceae; g__Pseudochrobactrum | 0.0000 | 0 |
| ASV136 | d__Bacteria; p__Firmicutes; c__Bacilli; o__Lactobacillales; f__Streptococcaceae; g__Streptococcus | 0.0113 | 0 |
| ASV275 | d__Bacteria; p__Proteobacteria; c__Alphaproteobacteria; o__Sphingomonadales; f__Sphingomonadaceae; g__Sphingomonas | 0.0014 | 0 |
| ASV193 | d__Bacteria; p__Firmicutes; c__Clostridia; o__Peptostreptococcales-Tissierellales; f__Anaerovoracaceae; g__Family_XIII_AD3011_group | 0.0022 | 0 |
| ASV152 | d__Bacteria; p__Firmicutes; c__Clostridia; o__Oscillospirales; f__Oscillospiraceae; g__UCG-005 | 0.0021 | 0 |
| ASV281 | d__Bacteria; p__Cyanobacteria; c__Cyanobacteriia; o__Chloroplast; f__Chloroplast; g__Chloroplast | 0.0121 | 0 |
| ASV323 | d__Bacteria; p__Firmicutes; c__Bacilli; o__Erysipelotrichales; f__Erysipelotrichaceae; g__Erysipelothrix | 0.0000 | 0 |
| ASV268 | d__Bacteria; p__Proteobacteria; c__Gammaproteobacteria; o__Enterobacterales; f__Enterobacteriaceae | 0.0047 | 0 |
| ASV245 | d__Bacteria; p__Proteobacteria; c__Gammaproteobacteria; o__Pasteurellales; f__Pasteurellaceae; g__Actinobacillus | 0.0906 | 0 |
| ASV258 | d__Bacteria; p__Actinobacteriota; c__Actinobacteria; o__Corynebacteriales; f__Dietziaceae; g__Dietzia | 0.0005 | 0 |
| ASV138 | d__Bacteria; p__Firmicutes; c__Clostridia; o__Peptostreptococcales-Tissierellales; f__Anaerovoracaceae; g__Family_XIII_AD3011_group | 0.0036 | 0 |
| ASV66 | d__Bacteria; p__Proteobacteria; c__Gammaproteobacteria; o__Pasteurellales; f__Pasteurellaceae; g__Actinobacillus | 0.9744 | 0 |
| ASV495 | d__Bacteria; p__Firmicutes; c__Clostridia; o__Clostridia_UCG-014; f__Clostridia_UCG-014; g__Clostridia_UCG-014 | 0.0019 | 0 |
| ASV333 | d__Bacteria; p__Actinobacteriota; c__Actinobacteria; o__Micrococcales; f__Brevibacteriaceae; g__Brevibacterium | 0.0000 | 0 |
| ASV401 | d__Bacteria; p__Firmicutes; c__Bacilli; o__Erysipelotrichales; f__Erysipelotrichaceae; g__Erysipelothrix | 0.0074 | 0 |
| ASV433 | d__Bacteria; p__Firmicutes; c__Bacilli; o__Lactobacillales; f__Aerococcaceae; g__Globicatella | 0.0032 | 0 |
| ASV206 | d__Bacteria; p__Firmicutes; c__Clostridia; o__Peptostreptococcales-Tissierellales; f__Anaerovoracaceae; g__[Eubacterium]_brachy_group | 0.0041 | 0 |
| ASV88 | d__Bacteria; p__Firmicutes; c__Clostridia; o__Lachnospirales; f__Lachnospiraceae; g__Lachnoclostridium | 0.0575 | 0 |
| ASV389 | d__Bacteria; p__Firmicutes; c__Bacilli; o__Lactobacillales; f__Streptococcaceae; g__Streptococcus | 0.1083 | 0 |
| ASV139 | d__Bacteria; p__Firmicutes; c__Clostridia; o__Lachnospirales; f__Lachnospiraceae | 0.0054 | 0 |
| ASV286 | d__Bacteria; p__Proteobacteria; c__Gammaproteobacteria; o__Pasteurellales; f__Pasteurellaceae; g__Actinobacillus | 0.0541 | 0 |
| ASV211 | d__Bacteria; p__Actinobacteriota; c__Actinobacteria; o__Corynebacteriales; f__Corynebacteriaceae; g__Corynebacterium | 0.0062 | 0 |
| ASV285 | d__Bacteria; p__Proteobacteria; c__Gammaproteobacteria; o__Pasteurellales; f__Pasteurellaceae; g__Actinobacillus | 0.1286 | 0 |
| ASV426 | d__Bacteria; p__Actinobacteriota; c__Actinobacteria; o__Propionibacteriales; f__Propionibacteriaceae; g__Pseudopropionibacterium | 0.0018 | 0 |
| ASV226 | d__Bacteria; p__Actinobacteriota; c__Actinobacteria; o__Corynebacteriales; f__Corynebacteriaceae; g__Corynebacterium | 0.0025 | 0 |
| ASV191 | d__Bacteria; p__Proteobacteria; c__Gammaproteobacteria; o__Pasteurellales; f__Pasteurellaceae; g__Actinobacillus | 0.0506 | 0 |
| ASV380 | d__Bacteria; p__Firmicutes; c__Clostridia; o__Peptostreptococcales-Tissierellales; f__Peptostreptococcales-Tissierellales; g__Helcococcus | 0.0140 | 0 |
| ASV461 | d__Bacteria; p__Firmicutes; c__Clostridia; o__Lachnospirales; f__Lachnospiraceae; g__Lachnospiraceae_AC2044_group | 0.0030 | 0 |
| ASV374 | d__Bacteria; p__Proteobacteria; c__Gammaproteobacteria; o__Pasteurellales; f__Pasteurellaceae; g__Actinobacillus | 0.0185 | 0 |
| ASV328 | d__Bacteria; p__Proteobacteria; c__Alphaproteobacteria; o__Rhizobiales; f__Rhizobiales_Incertae_Sedis; g__Phreatobacter | 0.0000 | 0 |
| ASV283 | d__Bacteria; p__Proteobacteria; c__Gammaproteobacteria; o__Pasteurellales; f__Pasteurellaceae; g__Actinobacillus | 0.2301 | 0 |
| ASV466 | d__Bacteria; p__Proteobacteria; c__Gammaproteobacteria; o__Pasteurellales; f__Pasteurellaceae; g__Actinobacillus | 0.0014 | 0 |
| ASV219 | d__Bacteria; p__Firmicutes; c__Clostridia; o__Lachnospirales; f__Lachnospiraceae | 0.2672 | 0 |
| ASV501 | d__Bacteria; p__Patescibacteria; c__Saccharimonadia; o__Saccharimonadales; f__Saccharimonadaceae; g__Candidatus_Saccharimonas | 0.0025 | 0 |
| ASV48 | d__Bacteria; p__Firmicutes; c__Bacilli; o__Lactobacillales; f__Streptococcaceae; g__Streptococcus | 0.0220 | 0 |
| ASV122 | d__Bacteria; p__Firmicutes; c__Clostridia; o__Oscillospirales; f__Oscillospiraceae; g__UCG-005 | 0.0036 | 0 |
| ASV112 | d__Bacteria; p__Firmicutes; c__Clostridia; o__Peptostreptococcales-Tissierellales; f__Anaerovoracaceae; g__Family_XIII_AD3011_group | 0.0051 | 0 |
| ASV310 | d__Bacteria; p__Actinobacteriota; c__Actinobacteria; o__Micrococcales; f__Dermabacteraceae; g__Brachybacterium | 0.0000 | 0 |
| ASV141 | d__Bacteria; p__Firmicutes; c__Negativicutes; o__Veillonellales-Selenomonadales; f__Veillonellaceae; g__Veillonella | 0.0236 | 0 |
| ASV482 | d__Bacteria; p__Actinobacteriota; c__Coriobacteriia; o__Coriobacteriales; f__Atopobiaceae; g__Atopobium | 0.0019 | 0 |
| ASV40 | d__Bacteria; p__Firmicutes; c__Clostridia; o__Peptostreptococcales-Tissierellales; f__Anaerovoracaceae; g__[Eubacterium]_saphenum_group | 0.0038 | 0 |
| ASV484 | d__Bacteria; p__Firmicutes; c__Negativicutes; o__Acidaminococcales; f__Acidaminococcaceae; g__Phascolarctobacterium | 0.0016 | 0 |
| ASV429 | d__Bacteria; p__Firmicutes; c__Bacilli; o__Lactobacillales; f__Streptococcaceae; g__Streptococcus | 0.0014 | 0 |
| ASV200 | d__Bacteria; p__Firmicutes; c__Clostridia; o__Clostridia; f__Hungateiclostridiaceae; g__Fastidiosipila | 0.0074 | 0 |
| ASV273 | d__Bacteria; p__Proteobacteria; c__Gammaproteobacteria; o__Enterobacterales; f__Enterobacteriaceae; g__Klebsiella | 0.0023 | 0 |
| ASV208 | d__Bacteria; p__Firmicutes; c__Bacilli; o__Staphylococcales; f__Staphylococcaceae; g__Staphylococcus | 0.0010 | 0 |
| ASV3 | d__Bacteria; p__Proteobacteria; c__Gammaproteobacteria; o__Pasteurellales; f__Pasteurellaceae; g__Actinobacillus | 0.1652 | 0 |
| ASV375 | d__Bacteria; p__Firmicutes; c__Bacilli; o__Staphylococcales; f__Gemellaceae; g__Gemella | 0.0708 | 0 |
| ASV113 | d__Bacteria; p__Firmicutes; c__Clostridia; o__Oscillospirales; f__Oscillospiraceae; g__NK4A214_group | 0.0069 | 0 |
| ASV14 | d__Bacteria; p__Fusobacteriota; c__Fusobacteriia; o__Fusobacteriales; f__Leptotrichiaceae; g__Leptotrichia | 0.0987 | 0 |
| ASV108 | d__Bacteria; p__Firmicutes; c__Clostridia; o__Oscillospirales; f__Oscillospiraceae; g__NK4A214_group | 0.0080 | 0 |
| ASV45 | d__Bacteria; p__Actinobacteriota; c__Coriobacteriia; o__Coriobacteriales; f__Atopobiaceae; g__uncultured | 0.0317 | 0 |
| ASV178 | d__Bacteria; p__Firmicutes; c__Clostridia; o__Lachnospirales; f__Lachnospiraceae; g__Blautia | 0.0019 | 0 |
| ASV180 | d__Bacteria; p__Firmicutes; c__Clostridia; o__Oscillospirales; f__Ruminococcaceae; g__Incertae_Sedis | 0.0016 | 0 |
| ASV395 | d__Bacteria; p__Firmicutes; c__Clostridia; o__Peptococcales; f__Peptococcaceae; g__Peptococcus | 0.0250 | 0 |
| ASV253 | d__Bacteria; p__Firmicutes; c__Clostridia; o__Lachnospirales; f__Lachnospiraceae | 0.0114 | 0 |
| ASV417 | d__Bacteria; p__Firmicutes; c__Bacilli; o__Staphylococcales; f__Gemellaceae; g__Gemella | 0.0016 | 0 |
| ASV241 | d__Bacteria; p__Firmicutes; c__Bacilli; o__Lactobacillales; f__Streptococcaceae; g__Streptococcus | 0.0847 | 0 |
| ASV27 | d__Bacteria; p__Firmicutes; c__Clostridia; o__Oscillospirales; f__Oscillospiraceae; g__UCG-005 | 0.0384 | 0 |
| ASV397 | d__Bacteria; p__Actinobacteriota; c__Actinobacteria; o__Micrococcales; f__Microbacteriaceae | 0.0199 | 0 |
| ASV114 | d__Bacteria; p__Firmicutes; c__Bacilli; o__Lactobacillales; f__Streptococcaceae; g__Streptococcus | 0.0344 | 0 |
| ASV502 | d__Bacteria; p__Firmicutes; c__Clostridia; o__Peptostreptococcales-Tissierellales; f__Anaerovoracaceae; g__[Eubacterium]_brachy_group | 0.0021 | 0 |
| ASV490 | d__Bacteria; p__Firmicutes; c__Bacilli; o__Lactobacillales; f__Streptococcaceae; g__Streptococcus | 0.0128 | 0 |
| ASV143 | d__Bacteria; p__Firmicutes; c__Clostridia; o__Oscillospirales; f__Ruminococcaceae; g__uncultured | 0.0015 | 0 |
| ASV295 | d__Bacteria; p__Firmicutes; c__Clostridia; o__Lachnospirales; f__Lachnospiraceae; g__Lachnospiraceae_FCS020_group | 0.0018 | 0 |
| ASV492 | d__Bacteria; p__Firmicutes; c__Clostridia; o__Lachnospirales; f__Lachnospiraceae; g__Blautia | 0.0014 | 0 |
| ASV210 | d__Bacteria; p__Firmicutes; c__Clostridia; o__Lachnospirales; f__Lachnospiraceae | 0.0037 | 0 |
| ASV201 | d__Bacteria; p__Actinobacteriota; c__Actinobacteria; o__Propionibacteriales; f__Propionibacteriaceae; g__Tessaracoccus | 0.0350 | 0 |
| ASV165 | d__Bacteria; p__Firmicutes; c__Clostridia; o__Oscillospirales; f__Oscillospiraceae; g__UCG-002 | 0.0026 | 0 |
| ASV95 | d__Bacteria; p__Firmicutes; c__Clostridia; o__Oscillospirales; f__Oscillospiraceae; g__uncultured | 0.0077 | 0 |
| ASV62 | d__Bacteria; p__Firmicutes; c__Bacilli; o__Lactobacillales; f__Streptococcaceae; g__Streptococcus | 0.8943 | 0 |
| ASV409 | d__Bacteria; p__Firmicutes; c__Clostridia; o__Lachnospirales; f__Lachnospiraceae | 0.0043 | 0 |
| ASV100 | d__Bacteria; p__Firmicutes; c__Bacilli; o__Erysipelotrichales; f__Erysipelotrichaceae; g__Solobacterium | 0.0108 | 0 |
| ASV463 | d__Bacteria; p__Firmicutes; c__Bacilli; o__Lactobacillales; f__Streptococcaceae; g__Streptococcus | 0.0096 | 0 |
| ASV173 | d__Bacteria; p__Actinobacteriota; c__Coriobacteriia; o__Coriobacteriales; f__Atopobiaceae; g__Olsenella | 0.0029 | 0 |
| ASV234 | d__Bacteria; p__Proteobacteria; c__Alphaproteobacteria; o__Sphingomonadales; f__Sphingomonadaceae; g__Sphingomonas | 0.0019 | 0 |
| ASV274 | d__Bacteria; p__Actinobacteriota; c__Actinobacteria; o__Micrococcales; f__Brevibacteriaceae; g__Brevibacterium | 0.0004 | 0 |
| ASV271 | d__Bacteria; p__Proteobacteria; c__Gammaproteobacteria; o__Pasteurellales; f__Pasteurellaceae; g__Actinobacillus | 0.0012 | 0 |
| ASV218 | d__Bacteria; p__Actinobacteriota; c__Actinobacteria; o__Actinomycetales; f__Actinomycetaceae; g__Actinomyces | 0.0014 | 0 |
| ASV103 | d__Bacteria; p__Firmicutes; c__Bacilli; o__Erysipelotrichales; f__Erysipelotrichaceae; g__uncultured | 0.0226 | 0 |
| ASV175 | d__Bacteria; p__Proteobacteria; c__Gammaproteobacteria; o__Pasteurellales; f__Pasteurellaceae; g__Actinobacillus | 0.0785 | 0 |
| ASV406 | d__Bacteria; p__Firmicutes; c__Clostridia; o__Peptostreptococcales-Tissierellales; f__Anaerovoracaceae; g__[Eubacterium]_brachy_group | 0.0071 | 0 |
| ASV354 | d__Bacteria; p__Proteobacteria; c__Alphaproteobacteria; o__Sphingomonadales; f__Sphingomonadaceae; g__Novosphingobium | 0.0493 | 0 |
| ASV99 | d__Bacteria; p__Firmicutes; c__Clostridia; o__Peptostreptococcales-Tissierellales; f__Peptostreptococcaceae; g__Peptostreptococcus | 0.4748 | 0 |
| ASV338 | d__Bacteria; p__Firmicutes; c__Clostridia; o__Peptostreptococcales-Tissierellales; f__Peptostreptococcales-Tissierellales; g__Helcococcus | 0.0038 | 0 |
| ASV287 | d__Bacteria; p__Firmicutes; c__Bacilli; o__Staphylococcales; f__Staphylococcaceae; g__Staphylococcus | 0.0007 | 0 |
| ASV158 | d__Bacteria; p__Firmicutes; c__Clostridia; o__Christensenellales; f__Christensenellaceae; g__Christensenellaceae_R-7_group | 0.0029 | 0 |
| ASV360 | d__Bacteria; p__Bacteroidota; c__Bacteroidia; o__Flavobacteriales; f__Weeksellaceae; g__Chryseobacterium | 0.0037 | 0 |
| ASV125 | d__Bacteria; p__Firmicutes; c__Clostridia; o__Lachnospirales; f__Lachnospiraceae; g__Oribacterium | 0.0059 | 0 |
| ASV156 | d__Bacteria; p__Firmicutes; c__Clostridia; o__Lachnospirales; f__Lachnospiraceae; g__Lachnospiraceae_ND3007_group | 0.0033 | 0 |
| ASV377 | d__Bacteria; p__Firmicutes; c__Bacilli; o__Staphylococcales; f__Gemellaceae; g__Gemella | 0.0815 | 0 |
| ASV361 | d__Bacteria; p__Firmicutes; c__Clostridia; o__Lachnospirales; f__Lachnospiraceae; g__[Eubacterium]_hallii_group | 0.0014 | 0 |
| ASV301 | d__Bacteria; p__Proteobacteria; c__Gammaproteobacteria; o__Enterobacterales; f__Enterobacteriaceae | 0.0000 | 0 |
| ASV471 | d__Bacteria; p__Proteobacteria; c__Gammaproteobacteria; o__Pasteurellales; f__Pasteurellaceae; g__Actinobacillus | 0.0016 | 0 |
| ASV299 | d__Bacteria; p__Actinobacteriota; c__Actinobacteria; o__Corynebacteriales; f__Corynebacteriaceae; g__Corynebacterium | 0.0001 | 0 |
| ASV63 | d__Bacteria; p__Firmicutes; c__Bacilli; o__Lactobacillales; f__Streptococcaceae; g__Streptococcus | 0.1932 | 0 |
| ASV421 | d__Bacteria; p__Firmicutes; c__Clostridia; o__Lachnospirales; f__Lachnospiraceae | 0.0048 | 0 |
| ASV195 | d__Bacteria; p__Firmicutes; c__Negativicutes; o__Acidaminococcales; f__Acidaminococcaceae; g__Phascolarctobacterium | 0.0058 | 0 |
| ASV68 | d__Bacteria; p__Proteobacteria; c__Gammaproteobacteria; o__Pasteurellales; f__Pasteurellaceae; g__Actinobacillus | 0.1467 | 0 |
| ASV277 | d__Bacteria; p__Proteobacteria; c__Gammaproteobacteria; o__Pasteurellales; f__Pasteurellaceae; g__Actinobacillus | 0.1477 | 0 |
| ASV73 | d__Bacteria; p__Firmicutes; c__Clostridia; o__Peptostreptococcales-Tissierellales; f__Peptostreptococcales-Tissierellales; g__Anaerococcus | 0.0417 | 0 |
| ASV424 | d__Bacteria; p__Actinobacteriota; c__Actinobacteria; o__Actinomycetales; f__Actinomycetaceae; g__Actinomyces | 0.0027 | 0 |
| ASV378 | d__Bacteria; p__Firmicutes; c__Clostridia; o__Lachnospirales; f__Lachnospiraceae | 0.0214 | 0 |
| ASV431 | d__Bacteria; p__Firmicutes; c__Bacilli; o__Lactobacillales; f__Streptococcaceae; g__Streptococcus | 0.0093 | 0 |
| ASV229 | d__Bacteria; p__Actinobacteriota; c__Actinobacteria; o__Actinomycetales; f__Actinomycetaceae; g__Actinomyces | 0.0041 | 0 |
| ASV445 | d__Bacteria; p__Firmicutes; c__Clostridia; o__Peptostreptococcales-Tissierellales; f__Peptostreptococcales-Tissierellales; g__Parvimonas | 0.0016 | 0 |
| ASV455 | d__Bacteria; p__Patescibacteria; c__Saccharimonadia; o__Saccharimonadales; f__Saccharimonadaceae; g__Saccharimonadaceae | 0.0103 | 0 |
| ASV55 | d__Bacteria; p__Firmicutes; c__Clostridia; o__Oscillospirales; f__Oscillospiraceae; g__UCG-002 | 0.0535 | 0 |
| ASV266 | d__Bacteria; p__Proteobacteria; c__Gammaproteobacteria; o__Enterobacterales; f__Enterobacteriaceae; g__Klebsiella | 0.0084 | 0 |
| ASV134 | d__Bacteria; p__Firmicutes; c__Clostridia; o__Oscillospirales; f__Oscillospiraceae | 0.0032 | 0 |
| ASV251 | d__Bacteria; p__Firmicutes; c__Clostridia; o__Oscillospirales; f__Ruminococcaceae | 0.0043 | 0 |
| ASV126 | d__Bacteria; p__Firmicutes; c__Clostridia; o__Lachnospirales; f__Lachnospiraceae; g__Marvinbryantia | 0.0038 | 0 |
| ASV383 | d__Bacteria; p__Firmicutes; c__Bacilli; o__Lactobacillales; f__Streptococcaceae; g__Streptococcus | 0.0056 | 0 |
| ASV24 | d__Bacteria; p__Firmicutes; c__Clostridia; o__Peptococcales; f__Peptococcaceae; g__Peptococcus | 0.0803 | 0 |
| ASV224 | d__Bacteria; p__Actinobacteriota; c__Actinobacteria; o__Corynebacteriales; f__Corynebacteriaceae; g__Corynebacterium | 0.0003 | 0 |
| ASV160 | d__Bacteria; p__Firmicutes; c__Clostridia; o__Lachnospirales; f__Lachnospiraceae; g__Blautia | 0.0014 | 0 |
| ASV462 | d__Bacteria; p__Firmicutes; c__Negativicutes; o__Acidaminococcales; f__Acidaminococcaceae; g__Phascolarctobacterium | 0.0018 | 0 |
| ASV396 | d__Bacteria; p__Actinobacteriota; c__Actinobacteria; o__Actinomycetales; f__Actinomycetaceae; g__Actinomyces | 0.0236 | 0 |
| ASV119 | d__Bacteria; p__Firmicutes; c__Clostridia; o__Lachnospirales; f__Lachnospiraceae | 0.0081 | 0 |
| ASV441 | d__Bacteria; p__Firmicutes; c__Clostridia; o__Clostridia_UCG-014; f__Clostridia_UCG-014; g__Clostridia_UCG-014 | 0.0026 | 0 |
| ASV294 | d__Bacteria; p__Firmicutes; c__Bacilli; o__Lactobacillales; f__Streptococcaceae; g__Streptococcus | 0.0021 | 0 |
| ASV458 | d__Bacteria; p__Firmicutes; c__Bacilli; o__Lactobacillales; f__Streptococcaceae; g__Streptococcus | 0.0235 | 0 |
| ASV296 | d__Bacteria; p__Actinobacteriota; c__Actinobacteria; o__Corynebacteriales; f__Corynebacteriaceae; g__Corynebacterium | 0.0005 | 0 |
| ASV171 | d__Bacteria; p__Actinobacteriota; c__Coriobacteriia; o__Coriobacteriales; f__Coriobacteriaceae; g__Collinsella | 0.0644 | 0 |
| ASV46 | d__Bacteria; p__Proteobacteria; c__Gammaproteobacteria; o__Pasteurellales; f__Pasteurellaceae; g__Actinobacillus | 0.1007 | 0 |
